# Supplementary material for: Integrating multidimensional data analytics for precision diagnosis of chronic low back pain
Source: Sci Rep. 2025 Mar 20;15:9675. doi: 10.1038/s41598-025-93106-1 (PMC11926347; doi:10.1038/s41598-025-93106-1)
Supplement: Supplementary file 1 — Supplementary Information. [file 41598_2025_93106_MOESM1_ESM.pdf]

Integrating Multidimensional Data Analytics for Precision Diagnosis of Chronic Low Back Pain

Sam Vickery[1]*, Frederick Junker[1], Rebekka Döding[1], Daniel L Belavy[1], Maia Angelova[2,3], Chandan Karmakar[3], Luis Becker[4,5], Nima Taheri[4,5], Matthias Pumberger[4], Sandra Reitmaier[5], Hendrik Schmidt[5]

Supplementary

Table of Contents

Supplementary Data 1: STROBE & Tripod Checklist 5
Supplementary Table S1: STROBE Checklist. 5
Supplementary Table S2: TRIPOD Checklist 6
Supplementary Data 2: Study variables, preprocessing, and cleaning 8
Supplementary Table S3. Exclusion criteria and amount of participants excluded after arriving for testing. 8
Supplementary Table S4. Demographic data of cleaned questionnaire dataset 8
Supplementary Table S5. Demographic data of cleaned clinical assessment dataset 8
Supplementary Table S6. Demographic data of cleaned back shape and function dataset ... 9
Supplementary Table S7. Demographic data of cleaned MRI dataset 9
Supplementary Table S8. Demographic data of cleaned questionnaire + clinic dataset 10
Supplementary Table S9. Demographic data of cleaned questionnaire + back shape and function dataset 10
Supplementary Table S10. Demographic data of cleaned questionnaire + MRI dataset 10
Supplementary Table S11. Demographic data of cleaned clinic + back shape and function dataset 11
Supplementary Table S12. Demographic data of cleaned clinic + MRI dataset 11
Supplementary Table S13. Demographic data of cleaned back shape and function + MRI dataset 11
Supplementary Table S14. Demographic data of cleaned questionnaire + clinic + back shape and function dataset 12
Supplementary Table S15. Demographic data of cleaned questionnaire + clinic + MRI dataset 12
Supplementary Table S16. Demographic data of cleaned questionnaire + back shape and function + MRI dataset 12

Supplementary Table S17. Demographic data of cleaned clinic + back shape and function + MRI dataset 13
Supplementary Table S18. Demographic data of cleaned questoinmaire + clinic + back shape and function + MRI dataset 13
Supplementary Table S19. List of all variables in the Berlin Back Study. 13
Supplementary Table S20. List of all variables removed during preprocessing and the reason. 45
Supplementary Table S21: List of all variables remaing after preprocessing and cleaning for modelling 51
Supplementary Figure S1. Spearman correlation matrix of questionnaire dataset before removal of highly correlated variables 55
Supplementary Figure S2. Spearman correlation matrix of questionnaire dataset after removal of highly correlated variables 56
Supplementary Figure S3. Spearman correlation matrix of clinical assessment dataset 57
Supplementary Figure S4. Spearman correlation matrix of back shape and function dataset before removal of highly correlated variables 58
Supplementary Figure S5. Spearman correlation matrix of back shape and function dataset after removal of highly correlated variables 59
Supplementary Figure S6. Spearman correlation matrix of MRI dataset 60
Supplementary Data 3. Classification performance and feature reduction 60
Supplementary Table S22. Random forest classification performance. 60
Supplementary Table S23. Random forest classification performance (Imputaion) 62
Supplementary Table S24. Boruta selected important features in the questionnaire modality dataset. 64
Supplementary Figure S7. Spearman correlation matrix of Boruta selected important features for questionnaire dataset 65
Supplementary Table S25. Boruta selected important features for clinical physical assessment dataset 66
Supplementary Figure S8. Spearman correlation matrix of Boruta selected important features for clinical assessment dataset 67
Supplementary Table S26. Boruta selected important features for superficial spine morphology modality dataset 67
Supplementary Figure S9. Spearman correlation matrix of Boruta selected important features for back shape and function dataset 69
Supplementary Table S27. Boruta selected important features for MRI modality dataset . 69
Supplementary Figure S10. Spearman correlation matrix of Boruta selected important features for MRI dataset 70
Supplementary Table S28. Boruta selected important features for questionnaire and clinical physical assessment dataset 70
Supplementary Figure S11. Spearman correlation matrix of Boruta selected important features for questionnaire + clinic dataset 72
Supplementary Table S29. Boruta selected important features for clinic physical assessment and back shape and function dataset 72
Supplementary Figure S12. Spearman correlation matrix of Boruta selected important features for clinic physical assessment and back shape and function dataset 74

Supplementary Table S30. Boruta selected important features for MRI and questionnaire dataset ... 74
Supplementary Figure S13. Spearman correlation matrix of Boruta selected important features for questionnaire + MRI dataset ... 76
Supplementary Table S31. Boruta selected important features for MRI and clinical assessment modality dataset ... 76
Supplementary Figure S14. Spearman correlation matrix of Boruta selected important features for clinical assessment + MRI dataset ... 78
Supplementary Table S32. Boruta selected important features for MRI and superficial spine morphology dataset ... 78
Supplementary Figure S15. Spearman correlation matrix of Boruta selected important features for back shape and function + MRI dataset ... 80
Supplementary Table S33. Boruta selected important features for questionnaire and superficial spine morphology dataset ... 80
Supplementary Figure S16. Spearman correlation matrix of Boruta selected important features for questionnaire + back shape and function dataset ... 82
Supplementary Table S34. Boruta selected important features for questionnaire clinical assessment, and MRI dataset ... 82
Supplementary Figure S17. Spearman correlation matrix of Boruta selected important features for questionnaire + clinical assessment + MRI dataset ... 84
Supplementary Table S35. Boruta selected important features for clinical assessment, MRI, and superficial spine morphology dataset ... 84
Supplementary Figure S18. Spearman correlation matrix of Boruta selected important features for MRI + clinical assessment + back shape and function dataset ... 86
Supplementary Table S36. Boruta selected important features for questionnaire clinical assessment, and superficial spine morphology dataset ... 86
Supplementary Figure S19. Spearman correlation matrix of Boruta selected important features for questionnaire + clinical assessment + back shape and function dataset ... 88
Supplementary Table S37. Boruta selected important features for questionnaire, superficial spine morphology, and MRI dataset ... 88
Supplementary Figure S20. Spearman correlation matrix of Boruta selected important features for MRI + questionnaire + back shape and function dataset ... 90
Supplementary Table S38. Boruta selected important features for clinical assessment, MRI, questionnaire, and superficial spine morphology dataset ... 90
Supplementary Figure S21. Spearman correlation matrix of Boruta selected important features for questionnaire + clinical assessment + back shape and function + MRI dataset ... 92
Supplementary Table S39. Overall feature importance and robustness across all modality datasets ... 92
Supplementary Data 4: Univariate statistics ... 95
Supplementary Table S40: Demographic Wilcoxon-Mann-Whitney test (continuous and ordinal) ... 95
Supplementary Table S41: Demographic Chi2 Test (nominal) ... 95
Supplementary Table S42: Questionnaire Wilcoxon-Mann-Whitney test (continuous and ordinal) ... 95
Supplementary Table S43: Questionnaire Chi2 Test (nominal) ... 96

Supplementary Table S44: Clinical Physcial Assessment Wilcoxon-Mann-Whitney test (continuous and ordinal) ... 96
Supplementary Table S45: Clinical Physcial Assessment Chi2 Test (nominal) ... 97
Supplementary Table S46: Superficial spine morphology Wilcoxon-Mann-Whitney test (continuous) ... 97
Supplementary Table S47: Spino-pelvic MRI Wilcoxon-Mann-Whitney test (continuous and ordinal) ... 99

Supplementary Data 1: STROBE & Tripod Checklist

Supplementary Table S1: STROBE Checklist.

	Item No	Recommendation	Section or page number
**Title and abstract**	1	(*a*) Indicate the study's design with a commonly used term in the title or the abstract	1
		(*b*) Provide in the abstract an informative and balanced summary of what was done and what was found	2
**Introduction**			
Background/rationale	2	Explain the scientific background and rationale for the investigation being reported	4
Objectives	3	State specific objectives, including any prespecified hypotheses	5
**Methods**			
Study design	4	Present key elements of study design early in the paper	6
Setting	5	Describe the setting, locations, and relevant dates, including periods of recruitment, exposure, follow-up, and data collection	6, 19
Participants	6	(*a*) Give the eligibility criteria, and the sources and methods of selection of participants	6-7
Variables	7	Clearly define all outcomes, exposures, predictors, potential confounders, and effect modifiers. Give diagnostic criteria, if applicable	6-7, 19
Data sources/ measurement	8*	For each variable of interest, give sources of data and details of methods of assessment (measurement). Describe comparability of assessment methods if there is more than one group	6-7, 19-22
Bias	9	Describe any efforts to address potential sources of bias	6-7, 24-25
Study size	10	Explain how the study size was arrived at	-
Quantitative variables	11	Explain how quantitative variables were handled in the analyses. If applicable, describe which groupings were chosen and why	6-7, 24-25
Statistical methods	12	(*a*) Describe all statistical methods, including those used to control for confounding	26
		(*b*) Describe any methods used to examine subgroups and interactions	6-7, 24-25
		(c) Explain how missing data were addressed	6-7, 24-25
		(*d*) If applicable, describe analytical methods taking account of sampling strategy	Not applicable
		(*e*) Describe any sensitivity analyses	Not applicable
**Results**			
Participants	13*	(a) Report numbers of individuals at each stage of study—eg numbers potentially eligible, examined for eligibility, confirmed eligible, included in the study, completing follow-up, and analysed	6.7
		(b) Give reasons for non-participation at each stage	6-7
		(c) Consider use of a flow diagram	-
Descriptive data	14*	(a) Give characteristics of study participants (eg demographic, clinical, social) and information on exposures and potential confounders	7

		(b) Indicate number of participants with missing data for each variable of interest	6-7
Outcome data	15*	Report numbers of outcome events or summary measures	6-7
Main results	16	(*a*) Give unadjusted estimates and, if applicable, confounder-adjusted estimates and their precision (eg, 95% confidence interval). Make clear which confounders were adjusted for and why they were included	7-11
		(*b*) Report category boundaries when continuous variables were categorized	Not applicable
		© If relevant, consider translating estimates of relative risk into absolute risk for a meaningful time period	Not applicable
Other analyses	17	Report other analyses done—eg analyses of subgroups and interactions, and sensitivity analyses	Not applicable
**Discussion**			
Key results	18	Summarise key results with reference to study objectives	14
Limitations	19	Discuss limitations of the study, taking into account sources of potential bias or imprecision. Discuss both direction and magnitude of any potential bias	14, 16-18
Interpretation	20	Give a cautious overall interpretation of results considering objectives, limitations, multiplicity of analyses, results from similar studies, and other relevant evidence	14-16
Generalisability	21	Discuss the generalisability (external validity) of the study results	17-18
**Other information**			
Funding	22	Give the source of funding and the role of the funders for the present study and, if applicable, for the original study on which the present article is based	29

Supplementary Table S2: TRIPOD Checklist

Section/Topic	Item	Checklist Item	Page
**Title and abstract**			
Title	1	Identify the study as developing and/or validating a multivariable prediction model, the target population, and the outcome to be predicted.	1
Abstract	2	Provide a summary of objectives, study design, setting, participants, sample size, predictors, outcome, statistical analysis, results, and conclusions.	2
**Introduction**			
Background and objectives	3a	Explain the medical context (including whether diagnostic or prognostic) and rationale for developing or validating the multivariable prediction model, including references to existing models.	4
	3b	Specify the objectives, including whether the study describes the development or validation of the model or both.	5
**Methods**			
Source of data	4a	Describe the study design or source of data (e.g., randomized trial, cohort, or registry data), separately for the development and validation data sets, if applicable.	6, 19
	4b	Specify the key study dates, including start of accrual; end of accrual; and, if applicable, end of follow-up.	6, 19
Participants	5a	Specify key elements of the study setting (e.g., primary care, secondary care, general population) including number and location of centres.	6, 19
	5b	Describe eligibility criteria for participants.	6-7
	5c	Give details of treatments received, if relevant.	-
Outcome	6a	Clearly define the outcome that is predicted by the prediction model, including how and when assessed.	19, 24
	6b	Report any actions to blind assessment of the outcome to be predicted.	-
	7a	Clearly define all predictors used in developing or validating the multivariable	

Predictors		prediction model, including how and when they were measured.	
	7b	Report any actions to blind assessment of predictors for the outcome and other predictors.	-
Sample size	8	Explain how the study size was arrived at.	-
Missing data	9	Describe how missing data were handled (e.g., complete-case analysis, single imputation, multiple imputation) with details of any imputation method.	6-7, 24-25
Statistical analysis methods	10a	Describe how predictors were handled in the analyses.	19-22
	10b	Specify type of model, all model-building procedures (including any predictor selection), and method for internal validation.	26-28
	10d	Specify all measures used to assess model performance and, if relevant, to compare multiple models.	27-28
Risk groups	11	Provide details on how risk groups were created, if done.	-
**Results**			
Participants	13a	Describe the flow of participants through the study, including the number of participants with and without the outcome and, if applicable, a summary of the follow-up time. A diagram may be helpful.	6-7
	13b	Describe the characteristics of the participants (basic demographics, clinical features, available predictors), including the number of participants with missing data for predictors and outcome.	6-7
Model development	14a	Specify the number of participants and outcome events in each analysis.	6-7
	14b	If done, report the unadjusted association between each candidate predictor and outcome.	-
Model specification	15a	Present the full prediction model to allow predictions for individuals (i.e., all regression coefficients, and model intercept or baseline survival at a given time point).	7-11
	15b	Explain how to the use the prediction model.	-
Model performance	16	Report performance measures (with CIs) for the prediction model.	7-11
**Discussion**			
Limitations	18	Discuss any limitations of the study (such as nonrepresentative sample, few events per predictor, missing data).	14, 16-18
Interpretation	19b	Give an overall interpretation of the results, considering objectives, limitations, and results from similar studies, and other relevant evidence.	14-16
Implications	20	Discuss the potential clinical use of the model and implications for future research.	17-18
**Other information**			
Supplementary information	21	Provide information about the availability of supplementary resources, such as study protocol, Web calculator, and data sets.	Supplementary Data
Funding	22	Give the source of funding and the role of the funders for the present study.	29

Supplementary Data 2: Study variables, preprocessing, and cleaning

Supplementary Table S3. Exclusion criteria and amount of participants excluded after arriving for testing.

Exclusion Critera	Number Excluded
Professional atheletes	0
Substance abuse	0
Pregnant	0
BMI >28 kg/m^2	0
Central or peripheral neurologic impairments (e.g., spinal cord injury, radicular symptoms, sensory deficits)	4
Irritated, inflamed, and infected tissues in the measuring areas of the back	2
spinal fractures, osteoporosis, tumor diseases and bone metastases, and previous spinal surgery	21
strong drug therapy (opioids, muscle relaxants, antiepileptics), intake of long-acting antihistamines and/or systemic glucocorticoids, and/or immunosuppressive drugs	0
Rheumatic diseases and/or active systemic diseases (e.g., tuberculosis, collagenosis, multiple sclerosis, autoimmune diseases, acquired immune deficiency syndrome)	0
internal diseases that pose a potential risk to the study participants during the measurements (e.g., coronary heart diseases, chronic obstructive pulmonary disease) and/or influence the findings	0
heart insufficiency and/or malignant hypertension,	0
malpositions or anomalies of the lower extremities (e.g., knee or hip arthroplasty, arthrodesis)	2

Supplementary Table S4. Demographic data of cleaned questionnaire dataset

	Asymptomatic	Chronic low back pain
Sample size	555	431
Females	299	253
Age (years) mean [sd]	40.45 [12.36]	43.25 [11.54]
Body mass index mean [sd]	23.56 [2.74]	23.53 [2.83]
Pain duration (weeks) mean [sd]		563.67 [544.82]
Pain Intensity (VAS) mean [sd]		2.88 [1.85]

Supplementary Table S5. Demographic data of cleaned clinical assessment dataset

	Asymptomatic	Chronic low back pain

Sample size	596	484
Females	319	286
Age (years)<br>mean [sd]	40.5 [12.61]	43.58 [11.67]
Body mass index<br>mean [sd]	23.53 [2.74]	23.57 [2.82]
Pain duration (weeks)<br>mean [sd]		557.49 [532.35]
Pain Intensity (VAS)<br>mean [sd]		2.87 [1.84]

Supplementary Table S6. Demographic data of cleaned back shape and function dataset

	**Asymptomatic**	**Chronic low back pain**
Sample size	624	489
Females	337	299
Age (years)<br>mean [sd]	40.75 [12.59]	43.68 [11.71]
Body mass index<br>mean [sd]	23.53 [2.76]	23.55 [2.83]
Pain duration (weeks)<br>mean [sd]		548.87 [527.87]
Pain Intensity (VAS)<br>mean [sd]		2.9 [1.86]

Supplementary Table S7. Demographic data of cleaned MRI dataset

	**Asymptomatic**	**Chronic low back pain**
Sample size	456	343
Females	254	206
Age (years)<br>mean [sd]	41.46 [12.51]	44.4 [11.51]
Body mass index<br>mean [sd]	23.5 [2.77]	23.59 [2.72]
Pain duration (weeks)<br>mean [sd]		570.01 [551.03]
Pain Intensity (VAS)<br>mean [sd]		2.87 [1.86]

Supplementary Table S8. Demographic data of cleaned questionnaire + clinic dataset

	**Asymptomatic**	**Chronic low back pain**
Sample size	522	413
Females	278	240
Age (years) mean [sd]	40.3 [12.42]	43.42 [11.5]
Body mass index mean [sd]	23.56 [2.73]	23.53 [2.82]
Pain duration (weeks) mean [sd]		562.59 [543.63]
Pain Intensity (VAS) mean [sd]		2.86 [1.84]

Supplementary Table S9. Demographic data of cleaned questionnaire + back shape and function dataset

	**Asymptomatic**	**Chronic low back pain**
Sample size	530	409
Females	283	246
Age (years) mean [sd]	40.45 [12.36]	43.25 [11.54]
Body mass index mean [sd]	23.54 [2.74]	23.54 [2.82]
Pain duration (weeks) mean [sd]		563.61 [538.73]
Pain Intensity (VAS) mean [sd]		2.9 [1.86]

Supplementary Table S10. Demographic data of cleaned questionnaire + MRI dataset

	**Asymptomatic**	**Chronic low back pain**
Sample size	383	278
Females	212	162
Age (years) mean [sd]	41.35 [12.22]	44.19 [11.34]
Body mass index mean [sd]	23.5 [2.77]	23.6 [2.7]
Pain duration (weeks) mean [sd]		583.07 [569.01]

Pain Intensity (VAS) mean [sd]		2.87 [1.84]

Supplementary Table S11. Demographic data of cleaned clinic + back shape and function dataset

	**Asymptomatic**	**Chronic low back pain**
Sample size	573	463
Females	305	280
Age (years) mean [sd]	40.57 [12.59]	43.74 [11.74]
Body mass index mean [sd]	23.51 [2.74]	23.59 [2.84]
Pain duration (weeks) mean [sd]		555.8 [526.21]
Pain Intensity (VAS) mean [sd]		2.89 [1.85]

Supplementary Table S12. Demographic data of cleaned clinic + MRI dataset

	**Asymptomatic**	**Chronic low back pain**
Sample size	413	325
Females	228	194
Age (years) mean [sd]	41.26 [12.54]	44.5 [11.48]
Body mass index mean [sd]	23.44 [2.75]	23.61 [2.72]
Pain duration (weeks) mean [sd]		577.98 [551.55]
Pain Intensity (VAS) mean [sd]		2.87 [1.84]

Supplementary Table S13. Demographic data of cleaned back shape and function + MRI dataset

	**Asymptomatic**	**Chronic low back pain**
Sample size	448	334
Females	247	202
Age (years) mean [sd]	41.53 [12.54]	44.46 [11.5]
Body mass index mean [sd]	23.5 [2.78]	23.59 [2.74]
Pain duration (weeks) mean [sd]		560.32 [537.8]

Pain Intensity (VAS) mean [sd]		2.87 [1.87]

Supplementary Table S14. Demographic data of cleaned questionnaire + clinic + back shape and function dataset

	Asymptomatic	Chronic low back pain
Sample size	499	393
Females	264	234
Age (years) mean [sd]	40.38 [12.4]	43.56 [11.59]
Body mass index mean [sd]	23.54 [2.73]	23.56 [2.84]
Pain duration (weeks) mean [sd]		561.02 [536.44]
Pain Intensity (VAS) mean [sd]		2.88 [1.85]

Supplementary Table S15. Demographic data of cleaned questionnaire + clinic + MRI dataset

	Asymptomatic	Chronic low back pain
Sample size	356	267
Females	196	155
Age (years) mean [sd]	41.53 [12.54]	44.46 [11.5]
Body mass index mean [sd]	23.47 [2.76]	23.6 [2.71]
Pain duration (weeks) mean [sd]		581.41 [568.3]
Pain Intensity (VAS) mean [sd]		2.86 [1.84]

Supplementary Table S16. Demographic data of cleaned questionnaire + back shape and function + MRI dataset

	Asymptomatic	Chronic low back pain
Sample size	375	269
Females	205	158
Age (years) mean [sd]	41.42 [12.25]	44.27 [11.3]

Body mass index mean [sd]	23.5 [2.78]	23.62 [2.73]
Pain duration (weeks) mean [sd]		571.48 [533.96]
Pain Intensity (VAS) mean [sd]		2.87 [1.86]

Supplementary Table S17. Demographic data of cleaned clinic + back shape and function + MRI dataset

	Asymptomatic	Chronic low back pain
Sample size	406	317
Females	222	190
Age (years) mean [sd]	41.32 [12.56]	44.51 [11.52]
Body mass index mean [sd]	23.45 [2.76]	23.62 [2.74]
Pain duration (weeks) mean [sd]		566.27 [537.69]
Pain Intensity (VAS) mean [sd]		2.87 [1.85]

Supplementary Table S18. Demographic data of cleaned questoinmaire + clinic + back shape and function + MRI dataset

	Asymptomatic	Chronic low back pain
Sample size	349	259
Females	190	151
Age (years) mean [sd]	41.31 [12.36]	44.38 [11.32]
Body mass index mean [sd]	23.48 [2.77]	23.61 [2.74]
Pain duration (weeks) mean [sd]		567.21 [552.41]
Pain Intensity (VAS) mean [sd]		2.85 [1.85]

Supplementary Table S19. List of all variables in the Berlin Back Study.

Modality	Variable	Explanation	Units	Missing Values
Demographic	age	age of the participant at testing	years	17
Demographic	sex	sex of the participant at testing	Male, female	17

Demographic	clinic chronic LBP [Target]	classification of participant as having LBP, not having LBP, or having LBP in the past as diagnosed by a clinician	Yes, No, in past	23
Demographic	question chronic LBP	Participant answer of question do they suffer from chronic LBP	Yes, No	45
Demographic	LBP past pain duration	In past pain duration	days	1192
Demographic	LBP pain duration	pain duration	weeks	643
Demographic	height	height of participant as testing	cm	51
Demographic	weight	weigh tof participant at testing	Kg	52
Demographic	BMI	Body mass index	Kg/m2	52
Demographic	waist	diameter of waist	cm	108
Demographic	hip	diameter of hip	cm	107
Demographic	waist - hip ratio	ratio between hip and waist		108
Demographic	leg - left	length of left leg	cm	32
Demographic	leg - right	length of right leg	cm	32
Demographic	right- left leg ratio	ratio between leg length		32
Demographic	pain primary location	Loaction of primary pain site		40
Demographic	pain secondary location	Loaction of secondary pain site		224
Demographic	current pain	Visual Analoug Scale (VAS) 0-10		68
Questionnaire	pain begin	Participant answer of question how would they rate their pain at the moment?	1) sudden onset after exertion, 2) slowly increasing, 3) after an incorrect movement, 4) Progressive after previous low back pain, 5) after an accident	51
Questionnaire	pain coughing sneezing	Participant answer of question is their pain intensified when coughing or sneezing?	Yes, No	59
Questionnaire	pain standing	Participant answer of question is their pain intensified when standing?	Yes, No	59
Questionnaire	pain walking	Participant answer of question is their pain intensified when walking?	Yes, No	61
Questionnaire	pain sitting	Participant answer of question is their pain intensified when sitting?	Yes, No	62
Questionnaire	pain resting	Participant answer of question is their pain independent of exertion?	Yes, No	64

Questionnaire	pain day diff	Participant answer of question do they experience differences in pain intensity depending on the time of day?	Yes, No	67
Questionnaire	pain when strongest	Participant answer of question when is their pain most severe?	1) only during the day<br>2) only at night<br>3) always equally strong<br>4) Days > Nights<br>5) Nights > Days	79
Questionnaire	pain arm	Accompanying symptoms: pain arm	Yes, No	536
Questionnaire	pain leg	Accompanying symptoms: pain leg	Yes, No	528
Questionnaire	prior general disease	Symptoms such as fever or a general feeling of illness?	Yes, No	528
Questionnaire	pain walking distance	Participant answer of question which distance they can walk unrestricted.	unrestricted<br>1000 - 2000 m<br>500 - 1000 m<br>100 - 500 m<br>< 100 m	388
Questionnaire	pain movment limit	Participant answer of question is their movement restricted.	Yes, No	491
Questionnaire	pain sensitivity	Participant answer of question whether they have sensory disturbances/feeling disorders?	Yes, No	496
Questionnaire	pain paralysis	Participant answer of question whether they have sensory disturbances/feeling disorders?	Yes, No	513
Questionnaire	pain paralysis location	Participant answer of question for pain paralysis location	C1, C2, C3, C4, C5, C6, C7, C8, T1, T2, T3, T4, T5, T6, T7, T8, T9, T10, T11, T12, L1, L2, L3, L4, L5, S1, S2, S3, S4, S5, RH Anesthesia, Multisegmental, Arm, Multisegmental leg	1282
Questionnaire	sensory symptom	Participant answer of question whether they have sensory symptoms and if yes, where?	Bladder and rectal disorders<br>Genital area<br>Thigh<br>Lower leg<br>Outer edge of foot<br>sole of the foot<br>Instep<br>Toes<br>Hand	826

Questionnaire	sensory deficit	Participant answer of question whether they have sensory deficits.	Yes, No	540
Questionnaire	sensory location	…if yes, where?	C1, C2, C3, C4, C5, C6, C7, C8, T1, T2, T3, T4, T5, T6, T7, T8, T9, T10, T11, T12, L1, L2, L3, L4, L5, S1, S2, S3, S4, S5, RH Anesthesia, Multisegmental, Arm, Multisegmental leg	1216
Questionnaire	prior diagnosis	Participant answer of question whether they have prior diagnosis.	Yes, No	40
Questionnaire	prior diagnosis which	…if yes, which?	Intervertebral discs – protrusion, Intervertebral discs – prolapse, Intervertebral discs – sequestration, Spondylolisthesis, Spondylarthrosis, Osteochrondrosis, Spinal canal stenosis, Vertebral body fracture, Osteoporosis, Hip-left, Hip-right, Hip on both sides, Scoliosis, Multiple diagnosis spine	854
Questionnaire	prior diagnosis back level	…when prior back diagnosis which level?	L1, L2, L3, L4, L5, L1-L2, L2-L3, L3-L4, L4-L5, L5-S1, T1-T2, T2-T3, T3-T4, T4-T5, T5-T6, T6-T7, T7-T8, T8-T9, T9-T10, T10-T11, T11-T12, Cervical	1081
Questionnaire	prior surgeries	Participant answer of question whether they have prior surgeries.	Yes, No	96

Questionnaire	prior surgeries which	…if yes, which?	Spondylodesis, decompression surgery, disc surgery, vertebroplasty, kyphoplasty, vertebral body stenting, hip-left, hip-right, hip-bilateral, knee-left, knee-right, knee-bilateral, ankle-left, ankle-right, ankle-bilateral, foot-left, foot-right, foot-bilateral, Multiple pre-ops	1161
Questionnaire	prior surgeries spine level	…when prior spine surgeries which level?	L1, L2, L3, L4, L5, L1-L2, L2-L3, L3-L4, L4-L5, L5-S1, T1-T2, T2-T3, T3-T4, T4-T5, T5-T6, T6-T7, T7-T8, T8-T9, T9-T10, T10-T11, T11-T12	1293
Questionnaire	physically active for at least 150 min / week	Participant answer of question whether they are physically active for at least 150 min / week	1) No, and I don't plan to<br>2) No, but I am thinking about becoming more active<br>3) No, but I am determined to do so<br>4) Yes, but I find it difficult<br>5) Yes, and I find it very easy	38
Questionnaire	covid19 activity	Participant answer of question whether covid 19 affected their everyday functionality	substantial, moderate, hardly, not	24
Questionnaire	alcohol frequency	Participant answer of question how often do they drink alcohol.	never, less than once a month, two to four times a month, two to three times a week, four or more times a week	23
Questionnaire	alcohol number glasses	… if yes, how many glasses do they drink in a day?	1-2, 3-4, 5-6, 7-9, 10 or more	26
Questionnaire	alcohol restriction	Participants' response to the question of how often their alcohol consumption has prevented them from doing what was expected of them in the last 12 months.	never, less than once a month, once a month, once a week, daily or almost daily	28
Questionnaire	smoking	Participants' response to the question do they currently smoke?	Yes, No	24

Questionnaire	smoking pack years	…if yes, how many pack years	Cigarette packs per year	417
Questionnaire	medication 1 registration	Participants' response to the question what medication they take regularly	No medication, NSAIDs: Acetylsalicylic acid, NSAIDs: Ibuprofen, NSAIDs: Diclofenac, NSAIDs: Indomethacin, NSAIDs: Aspirin, NSAIDs: Coxibe, NSAIDs: Novaminsulfon, Analgesics: Paracetamol, Corticosteroids: hydrocortisone, Corticosteroids: prednisolone, Local anesthetics, Local anesthetics plus corticosteroids, Muscle relaxants: Tizanidine, muscle relaxants: Methocarbamol, Flupirtine, Opioids: morphine, Opioids: fentanyl, Opioids: fentanyl patches, Opioids: tramadol, Opioids: tilidine, Opioids: capsaicin patches and creams, Opioids: oxycondon, Opioids: tapentadol, Antidepressants: amitriptyline, Antidepressants: doxepin, Antidepressants: clomipramine, Antidepressants: escitalopram, Antidepressants: citalopram, Antidepressants: sertraline, Antidepressants: mirtazapine, Antidepressants: not named, Neur analgesics: Gabapentin, Neur analgesics: Pregabalin, Benzodiazepine,	130

			sleeping pills, Combination preparations: keltican, intigrin antagonist: α4β7 integrin antagonist, herbal remedy: devil's claw, herbal remedy: green-lipped mussel	
Questionnaire	medication 1 dosis mg	…if yes what dose?		1255
Questionnaire	medication 1 morning	…if yes what dose?		1259
Questionnaire	medication 1 midday	…if yes what dose?		1271
Questionnaire	medication 1 noon	…if yes what dose?		1265

Questionnaire	medication 2 registration	Participants' response to the question what second medication they take regulary	No medication, NSAIDs: Acetylsalicylic acid, NSAIDs: Ibuprofen, NSAIDs: Diclofenac, NSAIDs: Indomethacin, NSAIDs: Aspirin, NSAIDs: Coxibe, NSAIDs: Novaminsulfon, Analgesics: Paracetamol, Corticosteroids: hydrocortisone, Corticosteroids: prednisolone, Local anesthetics, Local anesthetics plus corticosteroids, Muscle relaxants: Tizanidine, muscle relaxants: Methocarbamol, Flupirtine, Opioids: morphine, Opioids: fentanyl, Opioids: fentanyl patches, Opioids: tramadol, Opioids: tilidine, Opioids: capsaicin patches and creams, Opioids: oxycondon, Opioids: tapentadol, Antidepressants: amitriptyline, Antidepressants: doxepin, Antidepressants: clomipramine, Antidepressants: escitalopram, Antidepressants: citalopram, Antidepressants: sertraline, Antidepressants: mirtazapine, Antidepressants: not named, Neur analgesics: Gabapentin, Neur analgesics: Pregabalin, Benzodiazepine, sleeping pills, Combination	310

			preparations: keltican, intigrin antagonist: α4β7 integrin antagonist, herbal remedy: devil's claw, herbal remedy: green-lipped mussel	
Questionnaire	medication 2 dosis mg	…if yes what dose?		1287
Questionnaire	medication 2 morning	…if yes what dose?		1288
Questionnaire	medication 2 midday	…if yes what dose?		1290
Questionnaire	medication 2 noon	…if yes what dose?		1289

Questionnaire	medication 1 demand	Participants' response to the question what medication they take on request	No medication, NSAIDs: Acetylsalicylic acid, NSAIDs: Ibuprofen, NSAIDs: Diclofenac, NSAIDs: Indomethacin, NSAIDs: Aspirin, NSAIDs: Coxibe, NSAIDs: Novaminsulfon, Analgesics: Paracetamol, Corticosteroids: hydrocortisone, Corticosteroids: prednisolone, Local anesthetics, Local anesthetics plus corticosteroids, Muscle relaxants: Tizanidine, muscle relaxants: Methocarbamol, Flupirtine, Opioids: morphine, Opioids: fentanyl, Opioids: fentanyl patches, Opioids: tramadol, Opioids: tilidine, Opioids: capsaicin patches and creams, Opioids: oxycondon, Opioids: tapentadol, Antidepressants: amitriptyline, Antidepressants: doxepin, Antidepressants: clomipramine, Antidepressants: escitalopram, Antidepressants: citalopram, Antidepressants: sertraline, Antidepressants: mirtazapine, Antidepressants: not named, Neur analgesics: Gabapentin, Neur analgesics: Pregabalin, Benzodiazepine, sleeping pills, Combination	130

			preparations: keltican, intigrin antagonist: α4β7 integrin antagonist, herbal remedy: devil's claw, herbal remedy: green-lipped mussel	
Questionnaire	medication 1 demand dosis mg	…if yes what dose?		624
Questionnaire	medication 1 demand month	…if yes what dose?		814

Questionnaire	medication 2 demand	Participants' response to the question what medication they take on request	No medication, NSAIDs: Acetylsalicylic acid, NSAIDs: Ibuprofen, NSAIDs: Diclofenac, NSAIDs: Indomethacin, NSAIDs: Aspirin, NSAIDs: Coxibe, NSAIDs: Novaminsulfon, Analgesics: Paracetamol, Corticosteroids: hydrocortisone, Corticosteroids: prednisolone, Local anesthetics, Local anesthetics plus corticosteroids, Muscle relaxants: Tizanidine, muscle relaxants: Methocarbamol, Flupirtine, Opioids: morphine, Opioids: fentanyl, Opioids: fentanyl patches, Opioids: tramadol, Opioids: tilidine, Opioids: capsaicin patches and creams, Opioids: oxycondon, Opioids: tapentadol, Antidepressants: amitriptyline, Antidepressants: doxepin, Antidepressants: clomipramine, Antidepressants: escitalopram, Antidepressants: citalopram, Antidepressants: sertraline, Antidepressants: mirtazapine, Antidepressants: not named, Neur analgesics: Gabapentin, Neur analgesics: Pregabalin, Benzodiazepine, sleeping pills, Combination	413

			preparations: keltican, intigrin antagonist: α4β7 integrin antagonist, herbal remedy: devil's claw, herbal remedy: green-lipped mussel	
Questionnaire	medication 2 demand dosis mg	…if a second medication is taken what dose?		1195
Questionnaire	medication 2 demand month	…if a second medication is taken which duration?	months	1222
Questionnaire	family back pain	Participants' response to the question is there back pain in their family?	Yes, No	51

Questionnaire	job field	Participants' response to the question in which professional field do they work?	Construction, architecture, surveying<br>Services<br>Electrical<br>Healthcare<br>IT, computers<br>art, culture, design<br>Agriculture, nature, environment<br>media,<br>metal, mechanical engineering<br>Natural sciences<br>Production, manufacturing<br>Social affairs, education<br>Technology, technology fields<br>Transportation, logistics<br>Economy, administration<br>Unemployed<br>Disabled/retired<br>Parental leave<br>Student<br>Pupil	22
Questionnaire	job posture	Participants' response to the question what is the primary posture that characterizes the professional field.	sitting, standing, sitting + standing, standing + walking, unemployed	25
Questionnaire	job posture duration	Participants' response to the question how the duration of the primary posture is.	Hours per day	321
Questionnaire	job load 1	Participants' response to the question of how they would characterize the primary physical workload in this area.	low load, moderate load, heavy load, vibrations, vibrations + heavy load, unemployed	34
Questionnaire	job load 1 duration	Participants' response to the question of how long the workload lasts.	Hours per day	62
Questionnaire	job load 2	Participants' response to the question of how they would characterize the secondary physical workload in this area.	low load, moderate load, heavy load, vibrations, vibrations + heavy load, unemployed	1019
Questionnaire	job load 2 duration	Participants' response to the question of how long the workload lasts.	Hours per day	1027

Questionnaire	job duration years	Participants' response to the question of how long they have been working in this area.	Years	59
Questionnaire	pyschological stress	Participants' response to the question of whether there are signs of psychosocial stress or strain due to their family situation.	Yes, No	23
Questionnaire	living situation	Participants' response to the question of whether they live alone or in a larger community.	alone, family, shared flat, home	23
Questionnaire	family conflicts	Participants' response to the question of whether family conflicts currently exist.	Yes, No	23
Questionnaire	self assessment	Participants' response to the question about their back functionality on a scale from 1 (very good) to 10 (severely limited).	1 to 10	23
Clinical	cervical inclination	The participants are asked to bend their cervical spine forward.	Degree	22
Clinical	cervical reclination	The participants are asked to bend their cervical spine backward.	Degree	22
Clinical	chin sternum distance		mm	25
Clinical	cervical lateral bend - left	The participants are asked to bend their cervical spine to the left.	Degree	23
Clinical	cervical lateral bend - right	The participants are asked to bend their cervical spine to the right.	Degree	23
Clinical	cervical axial rotate - left	The participants are asked to rotate their cervical spine to the left.	Degree	23
Clinical	cervical axial rotate - right	The participants are asked to rotate their cervical spine to the left.	Degree	24
Clinical	thoracic lumbar inclination	The participants are asked to perform maximum flexion.	Degree	22
Clinical	thoracic lumbar reclination	The participants are asked to perform maximum extension.	Degree	26
Clinical	finger floor distance	The participants are asked to perform maximum flexion.	mm	22
Clinical	hip flexion	The participants are asked to perform maximum flexion.	Degree	300

Clinical	thoracic lumbar lateral bending - left	The participants are asked to perform maximum lateral bending to the left.	Degree	24
Clinical	thoracic lumbar lateral bending - right	The participants are asked to perform maximum lateral bending to the right.	Degree	23
Clinical	thoracic lumbar axial rotation - left	The participants are asked to perform maximum axial rotation to the left.	Degree	28
Clinical	thoracic lumbar axial rotation - right	The participants are asked to perform maximum axial rotation to the right.	Degree	28
Clinical	motion OTT	The participants are asked to perform maximum flexion.	mm	24
Clinical	shober	The participants are asked to perform maximum flexion.	mm	23
Clinical	square shoulders	Participants are asked to stand upright.	Straight line, Right > Left, Left > Right	23
Clinical	plump line	Non-specific parameter with possible indication of scoliosis (spinal curvature), postural asymmetry, etc. Participants are asked to stand upright.	Yes, No	22
Clinical	rib hump	The ribs on one side of the spine are pushed backwards. Participants are asked to stand upright.	No, Right, Left	22
Clinical	lumbar bulge	When the upper body is bent forward, the back should look the same on the right and left side. The participants are asked to perform maximum flexion.	No, Right, Left	22
Clinical	asym waist triangle	Non-specific parameter with possible indication of scoliosis (spinal curvature), postural asymmetry, etc. Participants are asked to stand upright.	Yes, No	22

Clinical	back form	Normal findings: harmonious lumbar and thoracic spine curvature, flat back: reduced lumbar and thoracic spine curvature, gibbus: short-arched vertebral hump, hyperlordosis: increased lumbar curvature, hyperkyphosis: increased curvature of the thoracic spine, scoliosis: lateral deviation of the spine from the longitudinal axis with rotation (twisting) of the vertebrae around the longitudinal axis and torsion of the vertebral bodies. Participants are asked to stand upright.	Normal findings, Flat back, Gibbus, Hyperlordosis, Hyperkyphosis, Scoliosis,	22
Clinical	roussoly type	Lateral assessment of the spinal profile according to Roussouly. Participants are asked to stand upright.	1, 2, 3, 4	24
Clinical	sagittal balance	Lateral assessment of the spinal profile to determine whether the perpendicular of the center of the 7th cervical vertebra is approximately in line with the upper edge of the sacrum (part of the posterior pelvic ring). Participants are asked to stand upright.	Yes, No	22
Clinical	coronal balance	Assessment of the spinal profile from the front to determine whether the perpendicular of the center of the 7th cervical vertebra represents the center of the body (the head and cervical spine are in a central position). Participants are asked to stand upright.	Yes, No	25
Clinical	back head wall distance	Remaining distance between the back of the head and the wall when the heels/buttocks/shoulders are in contact with the wall as an indication of restricted spinal mobility, standard value: 0 cm. Participants are asked to stand upright at a wall.	cm	39

Clinical	Muscular hard tension	Hardened/tense actual muscle parts	No,<br>Cervical,<br>Thoracic,<br>Lumbar,<br>Several back sections	26
Clinical	pain pressure	Pressure pain over the spinous processes of the vertebral bodies as an indication of functional or structural changes or tension.	No,<br>Cervical,<br>Thoracic,<br>Lumbar,<br>Several back sections	22
Clinical	pain knocking	Tapping pain over the spinous processes of the vertebral bodies as an indication of functional or structural changes or tensio	No,<br>Cervical,<br>Thoracic,<br>Lumbar,<br>Several back sections	22
Clinical	pain spinous process shake	Pain in the spinous processes of the vertebral bodies as an indication of functional or structural changes or tension.	No,<br>Cervical,<br>Thoracic,<br>Lumbar,<br>Several back sections	22
Clinical	pain mennell sign	Indication of functional or structural change of the ilio-sacral joint	Negative<br>Right Positive<br>Left positive<br>Positive on both sides	25
Clinical	pain jumping	Test to examine the mobility of the vertebrae in relation to each other	Yes, No	38
Clinical	General mobility	Rating of general mobility into three categories	Normal mobility,<br>Hypomobile,<br>Hypermobile,	37
Clinical	toe stand	Orienting test to examine for reduced strength or signs of paralysis of the calf muscles	Yes, No	22
Clinical	heel stand	Orientation test to examine for reduced strength or signs of paralysis of the foot lifter muscles	Yes, No	22
Clinical	pain heel drop	Pain provocation due to compression of the axial skeleton as an indication of functional or structural changes or inflammation	Yes, No	23
Clinical	trendelenburg sign	Sinking of the opposite hip when standing on one leg as a sign of insufficiency of the gluteal muscles (gluteus medius/minimus)	Negative<br>Right Positive<br>Left positive<br>Positive on both sides	23

Clinical	straight leg raise	Pain provocation in the back leg in the range of 20-60° hip flexion with the leg extended due to nerve stretching/stretching of the nerve roots as an indication of nerve (root) compression	Negative<br>Right Positive<br>Left positive<br>Positive on both sides	24
Clinical	pseudo lasegue - left	Pain provocation in the back leg in the range of 20-60° hip flexion with the leg extended due to nerve stretching/stretching of the nerve roots as an indication of nerve (root) compression	Degree	310
Clinical	pseudo lasegue - right	Pain provocation in the back leg in the range of 20-60° hip flexion with the leg extended due to nerve stretching/stretching of the nerve roots as an indication of nerve (root) compression	Degree	311
Clinical	gait pattern	Fluent gait: normal findings, Limping gait: non-specific gait asymmetry, Gait ataxia: unsteady gait, Stepper gait: altered gait due to foot drop palsy, Wernicke-Mann gait: altered gait after a central disorder (e.g. stroke), Duchenne limp: gait alteration due to muscular insufficiency of the small gluteal muscles	luid gait<br>Limping gait<br>gait ataxia<br>stepping gait<br>Wernicke-Mann gait pattern<br>Duchenne limp	24
Clinical	reflex quadricep - left	Quadriceps tendon reflex: Tensing of the anterior thigh muscle after striking the patellar tendon with a reflex hammer	Areflexia<br>Hyporeflexia<br>Normoreflexia<br>hyperreflexia	23
Clinical	reflex quadricep - right	Quadriceps tendon reflex: Tensing of the anterior thigh muscle after striking the patellar tendon with a reflex hammer	Areflexia<br>Hyporeflexia<br>Normoreflexia<br>hyperreflexia	24
Clinical	reflex achilles - left	Achilles tendon reflex: Tensing of the calf muscles after striking the Achilles tendon with a reflex hammer	Areflexia<br>Hyporeflexia<br>Normoreflexia<br>hyperreflexia	24
Clinical	reflex achilles - right	Achilles tendon reflex: Tensing of the calf muscles after striking the Achilles tendon with a reflex hammer	Areflexia<br>Hyporeflexia<br>Normoreflexia<br>hyperreflexia	27
Questionnaire	pain hip	Pain at the hip	No<br>Left<br>Right<br>Both sides	41

Clinical	hip flexion left	Flexion: hip flexion - left	Degree	36
Clinical	hip flexion - right	Flexion: hip flexion - right	Degree	36
Clinical	hip extension - left	Extension: hip extension - left	Degree	39
Clinical	hip extension - right	Extension: hip extension - right	Degree	41
Clinical	hip external rotate - left	External rotation: outward rotation of the hip joint - left	Degree	37
Clinical	hip external rotate - right	External rotation: outward rotation of the hip joint - right	Degree	36
Clinical	hip internal rotate - left	Internal rotation: inward rotation of the hip joint - left	Degree	37
Clinical	hip internal rotate - right	Internal rotation: inward rotation of the hip joint - right	Degree	36
Clinical	hip abduction - left	movement of the leg away from the midline of the body	Degree	36
Clinical	hip abduction - right	movement of the leg away from the midline of the body	Degree	36
Clinical	hip adduction - left	Abduction: spreading apart in the hip joint	Degree	37
Clinical	hip adduction - right	Adduction: spreading in the hip joint	Degree	37
Clinical	thomas handle - left	Thomas grip: indicative test for shortening of the hip flexor muscles	Positive, Negative	37
Clinical	thomas handle - right	Thomas grip: indicative test for shortening of the hip flexor muscles	Positive, Negative	39
Clinical	pelvic tilt frontal	Frontal pelvic position: Indication of leg length difference/postural asymmetry	Straight position<br>Left > Right<br>Right > Left	42
Clinical	pelvic tilt sagittal	Sagittal pelvic position: Different standard variants of the "pelvic tilt" viewed from the side	Straight position<br>Pool forward tilt<br>Pool back tilt	39
Clinical	sit to stand 30sec	The participant is instructed to complete as many full stands as possible within the 30 seconds	Number of complete stands	55
Clinical	heart rate		/ min	67
Clinical	breath rate		/ min	172
Clinical	heart systolic pressure		mmHg	46
Clinical	heart diastolic pressure		mmHg	48
Questionnaire	sf36 physical function	Heatlth questionnaire. Transformed into a 0-100 scale. The lower the score the more disability. The higher the score the less disability	0-100	41

Questionnaire	sf36 physical role function	Heatlth questionnaire. Transformed into a 0-100 scale. The lower the score the more disability. The higher the score the less disability	0-100	39
Questionnaire	sf36 physical pain	Heatlth questionnaire. Transformed into a 0-100 scale. The lower the score the more disability. The higher the score the less disability	0-100	36
Questionnaire	sf36 health perception	Heatlth questionnaire. Transformed into a 0-100 scale. The lower the score the more disability. The higher the score the less disability	0-100	34
Questionnaire	sf36 vitality	Heatlth questionnaire. Transformed into a 0-100 scale. The lower the score the more disability. The higher the score the less disability	0-100	36
Questionnaire	sf36 social function	Heatlth questionnaire. Transformed into a 0-100 scale. The lower the score the more disability. The higher the score the less disability	0-100	42
Questionnaire	sf36 emotional role function	Heatlth questionnaire. Transformed into a 0-100 scale. The lower the score the more disability. The higher the score the less disability	0-100	36
Questionnaire	sf36 psychological well being	Heatlth questionnaire. Transformed into a 0-100 scale. The lower the score the more disability. The higher the score the less disability	0-100	36
Questionnaire	therapy 1	Have you received pain treatment so far?	Yes, No	77
Questionnaire	therapy 2	Medications	Yes, No	105
Questionnaire	therapy 2 - 1	Effectiveness	Yes, partially, no	483
Questionnaire	therapy 3	Infusion	Yes, No	119
Questionnaire	therapy 3 - 1	Efectiveness	Yes, partially, no	852
Questionnaire	therapy 4	Injections into the area of pain, nerve blocks	Yes, No	109
Questionnaire	therapy 4 - 1	Efectiveness	Yes, partially, no	660
Questionnaire	therapy 5	Injections in the spinal cord (e.g. epidural)	Yes, No	119
Questionnaire	therapy 5 - 1	Efectiveness	Yes, partially, no	852
Questionnaire	therapy 6	Spinal cord probe (SCS) or pump systems	Yes, No	121

Questionnaire	therapy 6 - 1	Efectiveness	Yes, partially, no	891
Questionnaire	therapy 7	Physical therapy	Yes, No	93
Questionnaire	therapy 7 - 1	Efectiveness	Yes, partially, no	348
Questionnaire	therapy 8	Massages, baths, cold/heat therapy	Yes, No	101
Questionnaire	therapy 8 - 1	Efectiveness	Yes, partially, no	403
Questionnaire	therapy 9	Electrical nerve stimulation (TENS)	Yes, No	113
Questionnaire	therapy 9 - 1	Efectiveness	Yes, partially, no	729
Questionnaire	therapy 10	Acupuncture	Yes, No	108
Questionnaire	therapy 10 - 1	Efectiveness	Yes, partially, no	704
Questionnaire	therapy 11	Chiropratic	Yes, No	109
Questionnaire	therapy 11 - 1	Efectiveness	Yes, partially, no	685
Questionnaire	therapy 12	Psychotherapy	Yes, No	115
Questionnaire	therapy 12 - 1	Efectiveness	Yes, partially, no	784
Questionnaire	therapy 13	Relaxation techniques, hypnosis, biofeedback	Yes, No	116
Questionnaire	therapy 13 - 1	Efectiveness	Yes, partially, no	809
Questionnaire	therapy 14	Medication withdrawal	Yes, No	119
Questionnaire	therapy 14 - 1	Efectiveness	Yes, partially, no	896
Questionnaire	therapy 15	Spa/rehab treatment	Yes, No	113
Questionnaire	therapy 15 - 1	Efectiveness	Yes, partially, no	782
Questionnaire	employment currently	Are you currently working? (also applies if you are currently unable to work)	Yes, No	62
Questionnaire	employment unable work	Are you currently unable to work?	Yes, No	61
Questionnaire	employment unable work could return	If unable to work, do you think you will be able to return to your old job? Able to?	Yes, No	766
Questionnaire	employment unable work last 3 months	How many days have you been unable to work in the last 3 months?	0-92	151
Questionnaire	employment pension application	Do you intend to submit a pension application or an application to change your pension?	Yes, No	83
Questionnaire	employment pension application not decided	Have you submitted a pension application / application for a pension change that has not yet been decided?	Yes, No	86
Questionnaire	employment pension application rejected	Has a pension application already been rejected?	Yes, No	105
Questionnaire	employment pension application opposition	Is a pension application currently in the objection procedure?	Yes, No	104

Questionnaire	employment pension	Do you currently recieve a pension?	Yes, No	83
Questionnaire	employment pension for time	If Yes, for a fixed time?	Yes, No	780
Questionnaire	employment pension final	If Yes, for good?	Yes, No	786
Questionnaire	employment pension type	If so, what type of retirement?	Early retirement pension<br>Partial reduction in earning capacity<br>Full reduction in earning capacity<br>Occupational disability<br>Disability<br>Accident pension<br>Reaching the age limit	898
Questionnaire	employment disability	Do you have a recognized degree of disability (e.g. by the Office for Pension Matters)?	Yes, No	119
Questionnaire	employment disability grade	If so, how high is the disability grade	1-…	859
Questionnaire	chron 1	Which of the statements best applies to your pain in the last 12 weeks?	Constant pain with slight fluctuations<br>Continuous pain with strong fluctuations<br>Pain attacks in between pain-free<br>Pain attacks also pain in between<br>Had no pain in the last 12 weeks	236
Questionnaire	chron 2	If you suffer from pain attacks, please answer the following questions: 1) How often do these attacks occur on average?	several times a day<br>once a day<br>several times a week<br>once a week<br>several times a month<br>once a month	593
Questionnaire	chron 3	2) How long do these attacks last on average?	Seconds<br>minutes<br>hours<br>up to 3 days<br>longer than 3 days	589
Questionnaire	ipaq met vigorous	MET minutes of vigorous activity in a day	MET minutes	218
Questionnaire	ipaq met moderate	MET minutes of moderate activity in a day	MET minutes	197
Questionnaire	ipaq met walking	MET minutes of walking activity in a day	MET minutes	136
Questionnaire	ipaq met sitting	MET minutes of sitting activity in a day	MET minutes	67

Questionnaire	ipaq met sum	sum total of MET minutes in a day	MET minutes	37
Questionnaire	kinesiophobia	Total kinesiophobia value by summing the answers to 11 Tampa Scale for Kinesiophobia questions with values of 1-4 for do not agree at all, more or less disagree, more or less agree, completely agree	11 - 44 summation score	332
Questionnaire	srbai sum	Behavioural Automaticity summation score of 4 questionaire (1-6)	4 - 24 summation score	0
Questionnaire	brsq intrinsic motivation	summation score of BRSQ questions 1 & 3:<br>Q1 - I am happy and satisfied when I am physically active.<br>Q3 - I am physically active because I enjoy it.	1 - Does not apply at all<br>2 - Does not apply<br>3 - Does not really apply<br>4 - Rather true<br>5 - applies<br>6 - fully applies	33
Questionnaire	brsq integrated regulation	summation score of BRSQ questions 2 & 4:<br>Q2 - I consider physical activity to be an important part of me.<br>Q4 - I see physical activity as part of my identity.	1 - Does not apply at all<br>2 - Does not apply<br>3 - Does not really apply<br>4 - Rather true<br>5 - applies<br>6 - fully applies	34
Questionnaire	brsq external regulation	summation score of BRSQ questions 5 & 6:<br>Q5 - I consider physical activity to be an important part of me.<br>Q6 - I see physical activity as part of my identity.	1 - Does not apply at all<br>2 - Does not apply<br>3 - Does not really apply<br>4 - Rather true<br>5 - applies<br>6 - fully applies	34
Questionnaire	stress days	Summation of stress day questions 1 - 4:<br>Q1 - Little interest or pleasure in your activities<br>Q2 - Depression, melancholy or hopelessness<br>Q3 - Nervousness, anxiety or tension<br>Q4 - Not being able to stop or control worries	1 - not at all<br>2 - on individual days<br>3 - on more than half of the days<br>4 - almost every day	330

Questionnaire	stress score	Summation of stress day questions 5 - 8:<br>Q5 -How often in the last month have you had the feeling that you couldn't influence important things in your life?<br>Q6 - How often in the last month did you feel confident in dealing with personal tasks and problems?<br>Q7 - How often in the last month have you had the feeling that things are going your way?<br>Q8 - How often in the last month have you had the feeling that your problems have piled up to such an extent that you can no longer cope with them?	1 - never<br>2 - rarely<br>3 - sometimes<br>4 - often<br>5 - very often		330
Questionnaire	korff pain intensity	Average rating (1-10) from 3 Korff questionnaire questions:<br>Q2 - How would you classify your pain as it is at this moment?<br>Q3 - If you think about the days you have been in pain in the last three months, how would you rate your worst pain?<br>Q4 - If you think about the days you have had pain in the last three months, how would you rate the average intensity of the pain?		1:10	307

Questionnaire	korff disability	Average rating (1-10) from 4 Korff questionnaire questions:<br>Q5 - To what extent has your pain affected your daily activities in the last three months?<br>Q6 - To what extent has the pain affected your ability to participate in family or leisure activities in the last three months?<br>Q7 - To what extent has the pain affected your ability to do your work/homework in the last three months?<br>Q8 - To what extent has the pain affected your physical activities in your leisure time in the last three months?		1:10	327
Questionnaire	korff disability grading	Korff disability grading:<br>< 3 -> 0<br>3 - 5 -> 1<br>5-7 -> 2<br>> 7 -> 3	0-3		327
Questionnaire	korff days grading	Grading of days not able to carry out regular activities in the past 3 months:<br>< 4 -> 0<br>4 - 8 -> 1<br>8 - 16 -> 2<br>> 16 -> 3	0-3		306
Questionnaire	korff disability score	Summation score Korff disability grading and Korff days garding	0-6		335
Questionnaire	korff grading	Grading based on combination of Korff pain intensity (PI) and disability score (DS):<br>PI < 50 & DS < 3 -> 1<br>PI > 50 & DS < 3 -> 2<br>PI > 50 & DS 3 - 5 -> 3<br>PI > 50 & DS > 5 -> 4	1-4		335
Questionnaire	RM disability	Rolland Morris questionnaire summation score across 26 questions:<br>1 - True<br>0 - false	0-26		305
MRI	intervertebral disc oberservers	Intravertabral disc Pfirrmann classification rater code	1-3		408

MRI	intervertebral disc degeneration L1 - L2	Intravertabral disc L1-L2 Pfirrmann classification score	1-5	431
MRI	intervertebral disc degeneration L2 - L3	Intravertabral disc L2-L3 Pfirrmann classification score	1-5	431
MRI	intervertebral disc degeneration L3 - L4	Intravertabral disc L3-L4 Pfirrmann classification score	1-5	431
MRI	intervertebral disc degeneration L4 - L5	Intravertabral disc L4-L5 Pfirrmann classification score	1-5	431
MRI	intervertebral disc degeneration L5 - S1	Intravertabral disc L5-S1 Pfirrmann classification score	1-5	431
MRI	intervertebral disc degeneration L6 - S1	Intravertabral disc L6-S1 Pfirrmann classification score	1-5	1287
MRI	intervertebral disc herniation oberservers	intervertebral disc Kramer classification rater code	1-3	408
MRI	intervertebral disc herniation L1 - L2	intervertebral disc L1-L2 Kramer classification score	1-5	431
MRI	intervertebral disc herniation L2 - L3	intervertebral disc L2-L3 Kramer classification score	1-5	431
MRI	intervertebral disc herniation L3 - L4	intervertebral disc L3-L4 Kramer classification score	1-5	431
MRI	intervertebral disc herniation L4 - L5	intervertebral disc L4-L5 Kramer classification score	1-5	431
MRI	intervertebral disc herniation L5 - S1	intervertebral disc L5-S1 Kramer classification score	1-5	431
MRI	intervertebral disc herniation L6 - S1	intervertebral disc L6-S1 Kramer classification score	1-5	1288
MRI	facet joint oberservers	Facet joint arthosis with Fujiwara classification rate code	1-3	408
MRI	facet joint L1 - L2 left	Facet joint L1-L2 (left) arthosis Fujiwara classification score	1-4	432
MRI	facet joint L1 - L2 right	Facet joint L1-L2 (right) arthosis Fujiwara classification score	1-4	432
MRI	facet joint L2 - L3 left	Facet joint L2-L3 (left) arthosis Fujiwara classification score	1-4	432
MRI	facet joint L2 - L3 right	Facet joint L2-L3 (right) arthosis Fujiwara classification score	1-4	432
MRI	facet joint L3 - L4 left	Facet joint L3-L4 (left) arthosis Fujiwara classification score	1-4	431
MRI	facet joint L3 - L4 right	Facet joint L3-L4 (right) arthosis Fujiwara classification score	1-4	431

MRI	facet joint L4 - L5 left	Facet joint L4-L5 (left) arthosis Fujiwara classification score	1-4	431
MRI	facet joint L4 - L5 right	Facet joint L4-L5 (right) arthosis Fujiwara classification score	1-4	431
MRI	facet joint L5/S1 - L5/L6 left	Facet joint L5/S1-L5/L6 (left) arthosis Fujiwara classification score	1-4	431
MRI	facet joint L5/S1 - L5/L6 right	Facet joint L5/S1-L5/L6 (right) arthosis Fujiwara classification score	1-4	431
MRI	facet joint L6 - S1 left	Facet joint L6-S1 (left) arthosis Fujiwara classification score	1-4	1287
MRI	facet joint L6 - S1 right	Facet joint L6-S1 (right) arthosis Fujiwara classification score	1-4	1287
MRI	osteochondrosis intervertebralis oberservers	osteochondrosis intervertebralis rater	1-3	408
MRI	osteochondrosis intervertebralis L1 - L2	osteochondrosis intervertebralis L1-L2 score	0-2	431
MRI	osteochondrosis intervertebralis L2 - L3	osteochondrosis intervertebralis L2 L3 score	0-2	431
MRI	osteochondrosis intervertebralis L3 - L4	osteochondrosis intervertebralis L3-L4 score	0-2	431
MRI	osteochondrosis intervertebralis L4 - L5	osteochondrosis intervertebralis L4-L5 score	0-2	431
MRI	osteochondrosis intervertebralis L5 - S1	osteochondrosis intervertebralis L5-S1 score	0-2	431
MRI	osteochondrosis intervertebralis L6 - S1	osteochondrosis intervertebralis L6-S1 score	0-2	1295
MRI	spinal canal stenosis oberservers	spinal canal stenosis rater	1-3	408
MRI	spinal canal stenosis L1	spinal canal stenosis diameter	10-27mm	431
MRI	spinal canal stenosis L1	spinal canal stenosis diameter	10-27mm	431
MRI	spinal canal stenosis L2	spinal canal stenosis diameter	10-27mm	431
MRI	spinal canal stenosis L3	spinal canal stenosis diameter	10-27mm	431
MRI	spinal canal stenosis L4	spinal canal stenosis diameter	10-27mm	431
MRI	spinal canal stenosis L5	spinal canal stenosis diameter	10-27mm	1289
MRI	schizas oberservers	schizas spinal canal rater	1-3	408
MRI	schizas L1	schizas spinal canal L1	1-3	1004

MRI	schizas L2	schizas spinal canal L2	1-3	1004
MRI	schizas L3	schizas spinal canal L3	1-3	1004
MRI	schizas L4	schizas spinal canal L4	1-3	1004
MRI	schizas L5	schizas spinal canal L5	1-3	1004
MRI	schizas L6	schizas spinal canal L6	1-3	1296
MRI	lordosis oberservers	Lordosis grading rater	1-3	408
MRI	lordosis L1 - S1	Lordosis L1-S1	degrees	529
MRI	lordosis L1 - L2	Lordosis L1-L2	degrees	529
MRI	lordosis L2 - L3	Lordosis L2-L3	degrees	529
MRI	lordosis L3 - L4	Lordosis L3-L4	degrees	529
MRI	lordosis L4 - L5	Lordosis L4-L5	degrees	529
MRI	lordosis L5 - S1	Lordosis L5-L6	degrees	529
MRI	lordosis L6 - S1	Lordosis L6-S1	degrees	1290
Back shape & function	sacral frontal sit straight	sacral sipine segment angle during frontal sit straight motion	degrees	86
Back shape & function	thoracic value frontal sit straight	thoracic spine segment angle during frontal sit straight motion	degrees	86
Back shape & function	lumbar value frontal sit straight	lumbar spine segment angle during frontal sit straight motion	degrees	86
Back shape & function	inclination value frontal sit straight	inlcination value during frontal sit straight motion	degrees	86
Back shape & function	spine length frontal sit straight	spine length measured by spine mouse during frontal sit straight motion	mm	86
Back shape & function	surface length frontal sit straight	spine surface area measured by spine mouse during frontal sit straight motion	mm2	86
Back shape & function	sacral frontal sit left	sacral sipine segment angle during sit left motion	degrees	86
Back shape & function	thoracic value frontal sit left	thoracic spine segment angle during frontal sit left motion	degrees	86
Back shape & function	lumbar value frontal sit left	lumbar spine segment angle during frontal sit left motion	degrees	86
Back shape & function	inclination value frontal sit left	inlcination value during frontal sit left motion	degrees	86
Back shape & function	spine length frontal sit left	spine length measured by spine mouse during frontal sit left motion	mm	86
Back shape & function	surface length frontal sit left	spine surface area measured by spine mouse during frontal sit left motion	mm2	86
Back shape & function	sacral frontal sit right	sacral sipine segment angle during sit right motion	degrees	86
Back shape & function	thoracic value frontal sit right	thoracic spine segment angle during frontal sit right motion	degrees	86
Back shape & function	lumbar value frontal sit right	lumbar spine segment angle during frontal sit right motion	degrees	86

Back shape & function	inclination value frontal sit right	inlcination value during frontal sit right motion	degrees	86
Back shape & function	spine length frontal sit right	spine length measured by spine mouse during frontal sit right motion	mm	86
Back shape & function	surface length frontal sit right	spine surface area measured by spine mouse during frontal sit right motion	mm2	86
Back shape & function	sacral frontal stand straight	sacral sipine segment angle during stand straight motion	degrees	86
Back shape & function	thoracic value frontal stand straight	thoracic spine segment angle during frontal stand straight motion	degrees	86
Back shape & function	lumbar value frontal stand straight	lumbar spine segment angle during frontal stand straight motion	degrees	86
Back shape & function	inclination value frontal stand straight	inlcination value during frontal stand straight motion	degrees	86
Back shape & function	spine length frontal stand straight	spine length measured by spine mouse during frontal stand straight motion	mm	86
Back shape & function	surface length frontal stand straight	spine surface area measured by spine mouse during frontal stand straight motion	mm2	86
Back shape & function	sacral frontal stand left	sacral sipine segment angle during stand left motion	degrees	86
Back shape & function	thoracic value frontal stand left	thoracic spine segment angle during frontal stand left motion	degrees	86
Back shape & function	lumbar value frontal stand left	lumbar spine segment angle during frontal stand left motion	degrees	86
Back shape & function	inclination value frontal stand left	inlcination value during frontal stand left motion	degrees	86
Back shape & function	spine length frontal stand left	spine length measured by spine mouse during frontal stand left motion	mm	86
Back shape & function	surface length frontal stand left	spine surface area measured by spine mouse during frontal stand left motion	mm2	86
Back shape & function	sacral frontal stand right	sacral sipine segment angle during stand right motion	degrees	86
Back shape & function	thoracic value frontal stand right	thoracic spine segment angle during frontal stand right motion	degrees	86
Back shape & function	lumbar value frontal stand right	lumbar spine segment angle during frontal stand right motion	degrees	86
Back shape & function	inclination value frontal stand right	inlcination value during frontal stand right motion	degrees	86

Back shape & function	spine length frontal stand right	spine length measured by spine mouse during frontal stand right motion	mm	86
Back shape & function	surface length frontal stand right	spine surface area measured by spine mouse during frontal stand right motion	mm2	86
Back shape & function	sacral sagittal sit straight	sacral sipine segment angle during sagittal sit straight motion	degrees	86
Back shape & function	thoracic value sagittal sit straight	thoracic spine segment angle during sagittal sit straight motion	degrees	86
Back shape & function	lumbar value sagittal sit straight	lumbar spine segment angle during sagittal sit straight motion	degrees	86
Back shape & function	inclination value sagittal sit straight	inlcination value during sagittal sit straight motion	degrees	86
Back shape & function	spine length sagittal sit straight	spine length measured by spine mouse during frontal sit straight motion	mm	86
Back shape & function	surface length sagittal sit straight	spine surface area measured by spine mouse during frontal sit straight motion	mm2	86
Back shape & function	sacral sagittal sit extension	sacral sipine segment angle during sagittal sit extension motion	degrees	86
Back shape & function	thoracic value sagittal sit extension	thoracic spine segment angle during sagittal sit extension motion	degrees	86
Back shape & function	lumbar value sagittal sit extension	lumbar spine segment angle during sagittal sit extension motion	degrees	86
Back shape & function	inclination value sagittal sit extension	inlcination value during sagittal sit extension motion	degrees	86
Back shape & function	spine length sagittal sit extension	spine length measured by spine mouse during frontal sit extension motion	mm	86
Back shape & function	surface length sagittal sit extension	spine surface area measured by spine mouse during frontal sit extension motion	mm2	86
Back shape & function	sacral sagittal sit flexion	sacral sipine segment angle during sagittal sit flexion motion	degrees	86
Back shape & function	thoracic value sagittal sit flexion	thoracic spine segment angle during sagittal sit flexion motion	degrees	86
Back shape & function	lumbar value sagittal sit flexion	lumbar spine segment angle during sagittal sit flexion motion	degrees	86

Back shape & function	inclination value sagittal sit flexion	inlcination value during sagittal sit flexion motion	degrees	86
Back shape & function	spine length sagittal sit flexion	spine length measured by spine mouse during frontal sit flexion motion	mm	86
Back shape & function	surface length sagittal sit flexion	spine surface area measured by spine mouse during frontal sit flexion motion	mm2	86
Back shape & function	sacral sagittal stand straight	sacral sipine segment angle during sagittal stand straight motion	degrees	86
Back shape & function	thoracic value sagittal stand straight	thoracic spine segment angle during sagittal stand straight motion	degrees	86
Back shape & function	lumbar value sagittal stand straight	lumbar spine segment angle during sagittal stand straight motion	degrees	86
Back shape & function	inclination value sagittal stand straight	inlcination value during sagittal stand straight motion	degrees	86
Back shape & function	spine length sagittal stand straight	spine length measured by spine mouse during sagittal stand straight motion	mm	86
Back shape & function	surface length sagittal stand straight	spine surface area measured by spine mouse during sagittal stand straight motion	mm2	86
Back shape & function	sacral sagittal stand extension	sacral sipine segment angle during sagittal stand extension motion	degrees	86
Back shape & function	thoracic value sagittal stand extension	thoracic spine segment angle during sagittal stand extension motion	degrees	86
Back shape & function	lumbar value sagittal stand extension	lumbar spine segment angle during sagittal stand extension motion	degrees	86
Back shape & function	inclination value sagittal stand extension	inlcination value during sagittal stand extension motion	degrees	86
Back shape & function	spine length sagittal stand extension	spine length measured by spine mouse during sagittal stand extension motion	mm	86
Back shape & function	surface length sagittal stand extension	spine surface area measured by spine mouse during sagittal stand extension motion	mm2	86
Back shape & function	sacral sagittal stand flexion	sacral sipine segment angle during sagittal stand flexion motion	degrees	86

Back shape & function	thoracic value sagittal stand flexion	thoracic spine segment angle during sagittal stand flexion motion	degrees	86
Back shape & function	lumbar value sagittal stand flexion	lumbar spine segment angle during sagittal stand flexion motion	degrees	86
Back shape & function	inclination value sagittal stand flexion	inlcination value during sagittal stand flexion motion	degrees	86
Back shape & function	spine length sagittal stand flexion	spine length measured by spine mouse during sagittal stand flexion motion	mm	86
Back shape & function	surface length sagittal stand flexion	spine surface area measured by spine mouse during sagittal stand flexion motion	mm2	86

Supplementary Table S20. List of all variables removed during preprocessing and the reason.

**Modality**	**Variable**	**Reason**
Demographic	height	BMI was used instead of this related variable
Demographic	hip	BMI was used instead of this related variable
Demographic	LBP pain duration	Question only asked to LBP patients
Demographic	LBP past pain duration	Question only asked to LBP patients
Demographic	leg left	Not relevant to LBP classification as used for movement analyses
Demographic	leg right	Not relevant to LBP classification as used for movement analyses
Demographic	question chronic LBP	Clinical diagnosis was used as target
Demographic	right left leg ratio	Not relevant to LBP classification as used for movement analyses
Demographic	waist	BMI was used instead of this related variable
Demographic	weight	BMI was used instead of this related variable
Demographic	waist hip ratio	BMI was used instead of this related variable
Questionnaire	medication 1 demand	Medication clinical questions heavily biased towards LBP patients
Questionnaire	medication 1 demand dosis mg	Medication clinical questions heavily biased towards LBP patients
Questionnaire	medication 1 demand month	Medication clinical questions heavily biased towards LBP patients
Questionnaire	medication 1 dosis mg	Medication clinical questions heavily biased towards LBP patients
Questionnaire	medication 1 midday	Medication clinical questions heavily biased towards LBP patients
Questionnaire	medication 1 morning	Medication clinical questions heavily biased towards LBP patients
Questionnaire	medication 1 noon	Medication clinical questions heavily biased towards LBP patients
Questionnaire	medication 1 registration	Medication clinical questions heavily biased towards LBP patients

Questionnaire	medication 2 demand	Medication clinical questions heavily biased towards LBP patients
Questionnaire	medication 2 demand dosis mg	Medication clinical questions heavily biased towards LBP patients
Questionnaire	medication 2 demand month	Medication clinical questions heavily biased towards LBP patients
Questionnaire	medication 2 dosis mg	Medication clinical questions heavily biased towards LBP patients
Questionnaire	medication 2 midday	Medication clinical questions heavily biased towards LBP patients
Questionnaire	medication 2 morning	Medication clinical questions heavily biased towards LBP patients
Questionnaire	medication 2 noon	Medication clinical questions heavily biased towards LBP patients
Questionnaire	medication 2 registration	Medication clinical questions heavily biased towards LBP patients
Questionnaire	pain arm	Pain questions and tests on back heavily biased towards LBP pateints
Questionnaire	pain begin	Pain questions and tests on back heavily biased towards LBP pateints
Questionnaire	pain coughing sneezing	Pain questions and tests on back heavily biased towards LBP pateints
Questionnaire	pain day diff	Pain questions and tests on back heavily biased towards LBP pateints
Questionnaire	pain heel drop	Pain questions and tests on back heavily biased towards LBP pateints
Questionnaire	pain jumping	Pain questions and tests on back heavily biased towards LBP pateints
Questionnaire	pain knocking	Pain questions and tests on back heavily biased towards LBP pateints
Questionnaire	pain leg	Pain questions and tests on back heavily biased towards LBP pateints
Questionnaire	pain mennell sign	Pain questions and tests on back heavily biased towards LBP pateints
Questionnaire	pain movment limit	Pain questions and tests on back heavily biased towards LBP pateints
Questionnaire	pain paralysis	Pain questions and tests on back heavily biased towards LBP pateints
Questionnaire	pain paralysis location	Pain questions and tests on back heavily biased towards LBP pateints
Questionnaire	pain pressure	Pain questions and tests on back heavily biased towards LBP pateints
Questionnaire	pain primary location	Pain questions and tests on back heavily biased towards LBP pateints
Questionnaire	pain resting	Pain questions and tests on back heavily biased towards LBP pateints
Questionnaire	pain secondary location	Pain questions and tests on back heavily biased towards LBP pateints
Questionnaire	pain sensitivity	Pain questions and tests on back heavily biased towards LBP pateints
Questionnaire	pain sitting	Pain questions and tests on back heavily biased towards LBP pateints
Questionnaire	pain spinous process shake	Pain questions and tests on back heavily biased towards LBP pateints

Questionnaire	pain standing	Pain questions and tests on back heavily biased towards LBP pateints
Questionnaire	pain walking	Pain questions and tests on back heavily biased towards LBP pateints
Questionnaire	pain walking distance	Pain questions and tests on back heavily biased towards LBP pateints
Questionnaire	pain when strongest	Pain questions and tests on back heavily biased towards LBP pateints
Questionnaire	prior diagnosis	Prior diagnoses and surgery clincal questions bias towards LBP pateints
Questionnaire	prior diagnosis back level	Prior diagnoses and surgery clincal questions bias towards LBP pateints
Questionnaire	prior diagnosis which	Prior diagnoses and surgery clincal questions bias towards LBP pateints
Questionnaire	prior general disease	Prior diagnoses and surgery clincal questions bias towards LBP pateints
Questionnaire	prior surgeries spine level	Prior diagnoses and surgery clincal questions bias towards LBP pateints
Questionnaire	prior surgeries which	Very high correlation (> 0.9)
Questionnaire	alcohol restrict	Very low variance (0.48)
Questionnaire	body self assessment	Questions biased towards LBP patients
Questionnaire	job duration years	Job duration has high correlation with age
Questionnaire	job load 2	Questions with large amount of missing values
Questionnaire	job load 2 duration	Questions with large amount of missing values
Questionnaire	job posture duration	Questions with large amount of missing values
Questionnaire	smoking	Very high correlation (> 0.9)
Questionnaire	chron 1	Questions only asked of LBP patients
Questionnaire	chron 2	Questions only asked of LBP patients
Questionnaire	chron 3	Questions only asked of LBP patients
Questionnaire	employment currently	Questions only asked of LBP patients
Questionnaire	employment disability	Questions only asked of LBP patients
Questionnaire	employment disability grade	Questions only asked of LBP patients
Questionnaire	employment pension	Questions only asked of LBP patients
Questionnaire	employment pension application	Questions only asked of LBP patients
Questionnaire	employment pension application not decided	Questions only asked of LBP patients
Questionnaire	employment pension application opposition	Questions only asked of LBP patients
Questionnaire	employment pension application rejected	Questions only asked of LBP patients
Questionnaire	employment pension final	Questions only asked of LBP patients
Questionnaire	employment pension for time	Questions only asked of LBP patients
Questionnaire	employment pension type	Questions only asked of LBP patients
Questionnaire	employment unable work	Questions only asked of LBP patients
Questionnaire	employment unable work could return	Questions only asked of LBP patients
Questionnaire	employment unable work last 3 months	Questions only asked of LBP patients
Questionnaire	fabq physical activity	Questions only asked of LBP patients
Questionnaire	fabq workload	Questions only asked of LBP patients
Questionnaire	ipaq met moderate	Only used IPAQ sum score and not individual sub-scores

Questionnaire	ipaq met sitting	Only used IPAQ sum score and not individual sub-scores
Questionnaire	ipaq met vigorous	Only used IPAQ sum score and not individual sub-scores
Questionnaire	ipaq met walking	Only used IPAQ sum score and not individual sub-scores
Questionnaire	kinesiophobia	Questions only asked of LBP patients
Questionnaire	korff days grading	Questions only asked of LBP patients
Questionnaire	korff disability	Questions only asked of LBP patients
Questionnaire	korff disability grading	Questions only asked of LBP patients
Questionnaire	korff disability score	Questions only asked of LBP patients
Questionnaire	korff grading	Questions only asked of LBP patients
Questionnaire	korff pain intensity	Questions only asked of LBP patients
Questionnaire	RM disability	Questions only asked of LBP patients
Questionnaire	SF36 health perception	Questions biased towards LBP patients
Questionnaire	SF36 physical function	Questions biased towards LBP patients
Questionnaire	SF36 physical pain	Questions biased towards LBP patients
Questionnaire	SF36 physical role function	Questions biased towards LBP patients
Questionnaire	SF36 vitality	Questions biased towards LBP patients
Questionnaire	stress days	Questions only asked of LBP patients
Questionnaire	stress score	Questions only asked of LBP patients
Questionnaire	therapy 1	Questions biased towards LBP patients
Questionnaire	therapy 10	Questions biased towards LBP patients
Questionnaire	therapy 10 1	Questions biased towards LBP patients
Questionnaire	therapy 11	Questions biased towards LBP patients
Questionnaire	therapy 11 1	Questions biased towards LBP patients
Questionnaire	therapy 12	Questions biased towards LBP patients
Questionnaire	therapy 12 1	Questions biased towards LBP patients
Questionnaire	therapy 13	Questions biased towards LBP patients
Questionnaire	therapy 13 1	Questions biased towards LBP patients
Questionnaire	therapy 14	Questions biased towards LBP patients
Questionnaire	therapy 14 1	Questions biased towards LBP patients
Questionnaire	therapy 15	Questions biased towards LBP patients
Questionnaire	therapy 15 1	Questions biased towards LBP patients
Questionnaire	therapy 2	Questions biased towards LBP patients
Questionnaire	therapy 2 1	Questions biased towards LBP patients
Questionnaire	therapy 3	Questions biased towards LBP patients
Questionnaire	therapy 3 1	Questions biased towards LBP patients
Questionnaire	therapy 4	Questions biased towards LBP patients
Questionnaire	therapy 4 1	Questions biased towards LBP patients
Questionnaire	therapy 5	Questions biased towards LBP patients
Questionnaire	therapy 5 1	Questions biased towards LBP patients
Questionnaire	therapy 6	Questions biased towards LBP patients
Questionnaire	therapy 6 1	Questions biased towards LBP patients
Questionnaire	therapy 7	Questions biased towards LBP patients
Questionnaire	therapy 7 1	Questions biased towards LBP patients
Questionnaire	therapy 8	Questions biased towards LBP patients
Questionnaire	therapy 8 1	Questions biased towards LBP patients
Questionnaire	therapy 9	Questions biased towards LBP patients

Questionnaire	therapy 9 1	Questions biased towards LBP patients
Clinical	3 spine mouse measure	Conducting spine mouse 3x not related to LBP
Clinical	back head wall distance	Near zero variance (< 0.1)
Clinical	heel stand	Near zero variance (< 0.1)
Clinical	hip flexion	Large amount of missing values (> 250)
Clinical	toe stand	Near zero variance (< 0.1)
Clinical	breath rate	Heart rate, breath rate, and blood pressure not related to LBP
Clinical	heart diastolic pressure	Heart rate, breath rate, and blood pressure not related to LBP
Clinical	heart rate	Heart rate, breath rate, and blood pressure not related to LBP
Clinical	heart systolic pressure	Heart rate, breath rate, and blood pressure not related to LBP
Clinical	coronal balance	Near zero variance (< 0.1)
Clinical	gait pattern	Near zero variance (< 0.1)
Clinical	pseudo lasegue left	Large amount of missing values (> 250)
Clinical	pseudo lasegue right	Large amount of missing values (> 250)
Clinical	reflex achilles left	Near zero variance (< 0.1)
Clinical	reflex achilles right	Near zero variance (< 0.1)
Clinical	reflex quadricep left	Near zero variance (< 0.1)
Clinical	reflex quadricep right	Near zero variance (< 0.1)
Clinical	straight leg raise	Near zero variance (< 0.1)
Clinical	trendelenburg sign	Near zero variance (< 0.1)
Back shape & function	lumbar value sagittal stand flexion	High correlation (> 0.9)
Back shape & function	spine length frontal sit left	High correlation (> 0.9)
Back shape & function	spine length frontal sit right	High correlation (> 0.9)
Back shape & function	spine length frontal sit straight	High correlation (> 0.9)
Back shape & function	spine length frontal stand left	High correlation (> 0.9)
Back shape & function	spine length frontal stand right	High correlation (> 0.9)
Back shape & function	spine length frontal stand straight	High correlation (> 0.9)
Back shape & function	spine length sagittal sit extension	High correlation (> 0.9)
Back shape & function	spine length sagittal sit flexion	High correlation (> 0.9)
Back shape & function	spine length sagittal sit straight	High correlation (> 0.9)
Back shape & function	spine length sagittal stand flexion	High correlation (> 0.9)
Back shape & function	spine length sagittal stand straight	High correlation (> 0.9)
Back shape & function	surface length frontal sit left	High correlation (> 0.9)
Back shape & function	surface length frontal sit right	High correlation (> 0.9)

Back shape & function	surface length frontal sit straight	High correlation (> 0.9)
Back shape & function	surface length frontal stand left	High correlation (> 0.9)
Back shape & function	surface length frontal stand right	High correlation (> 0.9)
Back shape & function	surface length frontal stand straight	High correlation (> 0.9)
Back shape & function	surface length sagittal sit extension	High correlation (> 0.9)
Back shape & function	surface length sagittal sit flexion	High correlation (> 0.9)
Back shape & function	surface length sagittal sit straight	High correlation (> 0.9)
Back shape & function	surface length sagittal stand extension	High correlation (> 0.9)
Back shape & function	surface length sagittal stand flexion	High correlation (> 0.9)
Back shape & function	surface length sagittal stand straight	High correlation (> 0.9)
MRI	intervertebral disc herniation L1 - L2	Near zero variance (< 0.2)
MRI	intervertebral disc herniation L6 - S1	Very few (< 10) subjects had values for L6-S1
MRI	intervertebral disc herniation oberservers	radiologist rater info not relevant
MRI	facet joint L6 - S1 left	Very few (< 10) subjects had values for L6-S1
MRI	facet joint L6 - S1 right	Very few (< 10) subjects had values for L6-S1
MRI	facet joint oberservers	radiologist rater info not relevant
MRI	intervertebral disc degeneration L6 - S1	Very few (< 10) subjects had values for L6-S1
MRI	intervertebral disc oberservers	radiologist rater info not relevant
MRI	lordosis L1 - S1	Lordosis rating has > 100 missing values compared to other ratings
MRI	lordosis L1 - L2	Lordosis rating has > 100 missing values compared to other ratings
MRI	lordosis L2 - L3	Lordosis rating has > 100 missing values compared to other ratings
MRI	lordosis L3 - L4	Lordosis rating has > 100 missing values compared to other ratings
MRI	lordosis L4 - L5	Lordosis rating has > 100 missing values compared to other ratings
MRI	lordosis L5 - S1	Lordosis rating has > 100 missing values compared to other ratings
MRI	lordosis L6 - S1	Very few (< 10) subjects had values for L6-S1
MRI	lordosis oberservers	radiologist rater info not relevant
MRI	osteochondrosis intervertebralis L1 - L2	Near zero variance (< 0.2)
MRI	osteochondrosis intervertebralis L2 - L3	Near zero variance (< 0.2)
MRI	osteochondrosis intervertebralis L3 - L4	Near zero variance (< 0.2)
MRI	osteochondrosis intervertebralis L6 - S1	Very few (< 10) subjects had values for L6-S1

MRI	osteochondrosis intervertebralis oberservers	radiologist rater info not relevant
MRI	schizas L1	Schizas rating present in < 10 subjects
MRI	schizas L2	Schizas rating present in < 10 subjects
MRI	schizas L3	Schizas rating present in < 10 subjects
MRI	schizas L4	Schizas rating present in < 10 subjects
MRI	schizas L5	Schizas rating present in < 10 subjects
MRI	schizas L6	Very few (< 10) subjects had values for L6-S1
MRI	schizas oberservers	radiologist rater info not relevant
MRI	spinal canal stenosis L6	Very few (< 10) subjects had values for L6-S1
MRI	spinal canal stenosis oberservers	radiologist rater info not relevant

Supplementary Table S21: List of all variables remaing after preprocessing and cleaning for modelling

Modality	Variable	Data Type
Demographic	age	continuous
Demographic	sex	nominal
Demographic	clinic chronic LBP [Target]	nominal
Demographic	BMI	continuous
Questionnaire	prior surgeries	nominal
Questionnaire	physical activity	ordinal
Questionnaire	covid19 activity	ordinal
Questionnaire	alcohol freq	ordinal
Questionnaire	alcohol glasses	ordinal
Questionnaire	smoking pack years	continuous
Questionnaire	family back pain	nominal
Questionnaire	job field	nominal
Questionnaire	job posture	nominal
Questionnaire	job load 1	nominal
Questionnaire	job load 1 duration	continuous
Questionnaire	pyschological stress	nominal
Questionnaire	living situation	nominal
Questionnaire	family conflicts	nominal
Clinical	cervical inclination	continuous
Clinical	cervical reclination	continuous
Clinical	chin sternum distance	nominal
Clinical	cervical lat bend left	continuous
Clinical	cervical lat bend right	continuous
Clinical	cervical axial rotate left	continuous
Clinical	cervical axial rotate right	continuous
Clinical	thoracic lumbar inclination	continuous
Clinical	thoracic lumbar reclination	continuous
Clinical	finger floor distance	continuous
Clinical	thoracic lumbar lateral bend left	continuous
Clinical	thoracic lumbar lateral bend right	continuous
Clinical	thoracic lumbar axial rotation left	continuous
Clinical	thoracic lumbar axial rotation right	continuous

Clinical	OTT	continuous
Clinical	shober	continuous
Clinical	square shoulders	nominal
Clinical	plump line	nominal
Clinical	rib hump	nominal
Clinical	lumbar bulge	nominal
Clinical	asym waist triangle	nominal
Clinical	back form	nominal
Clinical	roussoly type	nominal
Clinical	sagittal balance	nominal
Clinical	rigid muscle	nominal
Clinical	general mobility	nominal
Questionnaire	hip pain	nominal
Clinical	hip flexion left	continuous
Clinical	hip flexion right	continuous
Clinical	hip extension left	continuous
Clinical	hip extension right	continuous
Clinical	hip external rotate left	continuous
Clinical	hip external rotate right	continuous
Clinical	hip internal rotate left	continuous
Clinical	hip internal rotate right	continuous
Clinical	hip abduction left	continuous
Clinical	hip abduction right	continuous
Clinical	hip adduction left	continuous
Clinical	hip adduction right	continuous
Clinical	thomas handle left	nominal
Clinical	thomas handle right	nominal
Clinical	pelvic tilt frontal	nominal
Clinical	pelvic tilt sagittal	nominal
Clinical	sit to stand 30sec	continuous
Questionnaire	SF36 social function	continuous
Questionnaire	SF36 emotional role function	continuous
Questionnaire	SF36 psychological well being	continuous
Questionnaire	IPAQ met sum	continuous
Questionnaire	SRBAI sum	continuous
Questionnaire	BRSQ intrinsic motivation	continuous
Questionnaire	BRSQ integrated regulation	continuous
Questionnaire	BRSQ external regulation	continuous
MRI	intervertebral disc degeneration L1 - L2	nominal
MRI	intervertebral disc degeneration L2 - L3	nominal
MRI	intervertebral disc degeneration L3 - L4	nominal
MRI	intervertebral disc degeneration L4 - L5	nominal
MRI	intervertebral disc degeneration L5 - S1	nominal
MRI	intervertebral disc herniation L2 - L3	nominal
MRI	intervertebral disc herniation L3 - L4	nominal
MRI	intervertebral disc herniation L4 - L5	nominal
MRI	intervertebral disc herniation L5 - S1	nominal
MRI	facet joint L1 - L2 left	nominal
MRI	facet joint L1 - L2 right	nominal

MRI	facet joint L2 - L3 left	nominal
MRI	facet joint L2 - L3 right	nominal
MRI	facet joint L3 - L4 left	nominal
MRI	facet joint L3 - L4 right	nominal
MRI	facet joint L4 - L5 left	nominal
MRI	facet joint L4 - L5 right	nominal
MRI	facet joint L5/S1 - L5/L6 left	nominal
MRI	facet joint L5/S1 - L5/L6 right	nominal
MRI	osteochondrosis intervertebralis L4 - L5	nominal
MRI	osteochondrosis intervertebralis L5 - S1	nominal
MRI	spinal canal stenosis L1	continuous
MRI	spinal canal stenosis L1	continuous
MRI	spinal canal stenosis L2	continuous
MRI	spinal canal stenosis L3	continuous
MRI	spinal canal stenosis L4	continuous
MRI	spinal canal stenosis L5	continuous
Back shape & function	thoracic value frontal sit straight	continuous
Back shape & function	lumbar value frontal sit straight	continuous
Back shape & function	inclination value frontal sit straight	continuous
Back shape & function	sacral frontal sit left	continuous
Back shape & function	thoracic value frontal sit left	continuous
Back shape & function	lumbar value frontal sit left	continuous
Back shape & function	inclination value frontal sit left	continuous
Back shape & function	sacral frontal sit right	continuous
Back shape & function	thoracic value frontal sit right	continuous
Back shape & function	lumbar value frontal sit right	continuous
Back shape & function	inclination value frontal sit right	continuous
Back shape & function	sacral frontal stand straight	continuous
Back shape & function	thoracic value frontal stand straight	continuous
Back shape & function	lumbar value frontal stand straight	continuous
Back shape & function	inclination value frontal stand straight	continuous
Back shape & function	sacral frontal stand left	continuous
Back shape & function	thoracic value frontal stand left	continuous
Back shape & function	lumbar value frontal stand left	continuous
Back shape & function	inclination value frontal stand left	continuous
Back shape & function	sacral frontal stand right	continuous
Back shape & function	thoracic value frontal stand right	continuous
Back shape & function	lumbar value frontal stand right	continuous
Back shape & function	inclination value frontal stand right	continuous
Back shape & function	sacral sagittal sit straight	continuous
Back shape & function	thoracic value sagittal sit straight	continuous
Back shape & function	lumbar value sagittal sit straight	continuous
Back shape & function	inclination value sagittal sit straight	continuous
Back shape & function	sacral sagittal sit extension	continuous
Back shape & function	thoracic value sagittal sit extension	continuous
Back shape & function	lumbar value sagittal sit extension	continuous
Back shape & function	inclination value sagittal sit extension	continuous
Back shape & function	sacral sagittal sit flexion	continuous

Back shape & function	thoracic value sagittal sit flexion	continuous
Back shape & function	lumbar value sagittal sit flexion	continuous
Back shape & function	inclination value sagittal sit flexion	continuous
Back shape & function	sacral sagittal stand straight	continuous
Back shape & function	thoracic value sagittal stand straight	continuous
Back shape & function	lumbar value sagittal stand straight	continuous
Back shape & function	inclination value sagittal stand straight	continuous
Back shape & function	sacral sagittal stand extension	continuous
Back shape & function	thoracic value sagittal stand extension	continuous
Back shape & function	lumbar value sagittal stand extension	continuous
Back shape & function	inclination value sagittal stand extension	continuous
Back shape & function	spine length sagittal stand extension	continuous
Back shape & function	sacral sagittal stand flexion	continuous
Back shape & function	thoracic value sagittal stand flexion	continuous
Back shape & function	inclination value sagittal stand flexion	continuous

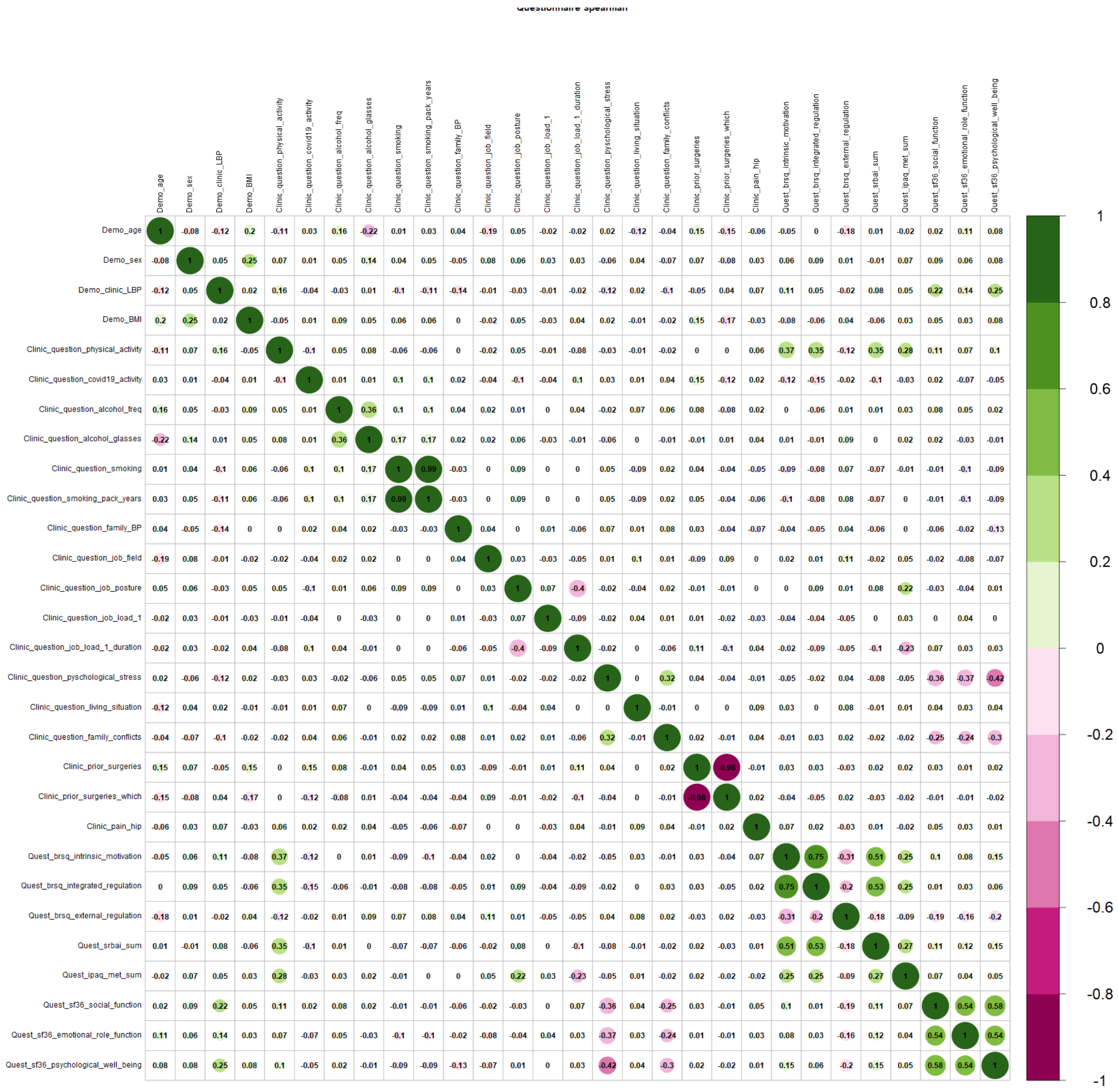

Supplementary Figure S1. Spearman correlation matrix of questionnaire dataset before removal of highly correlated variables

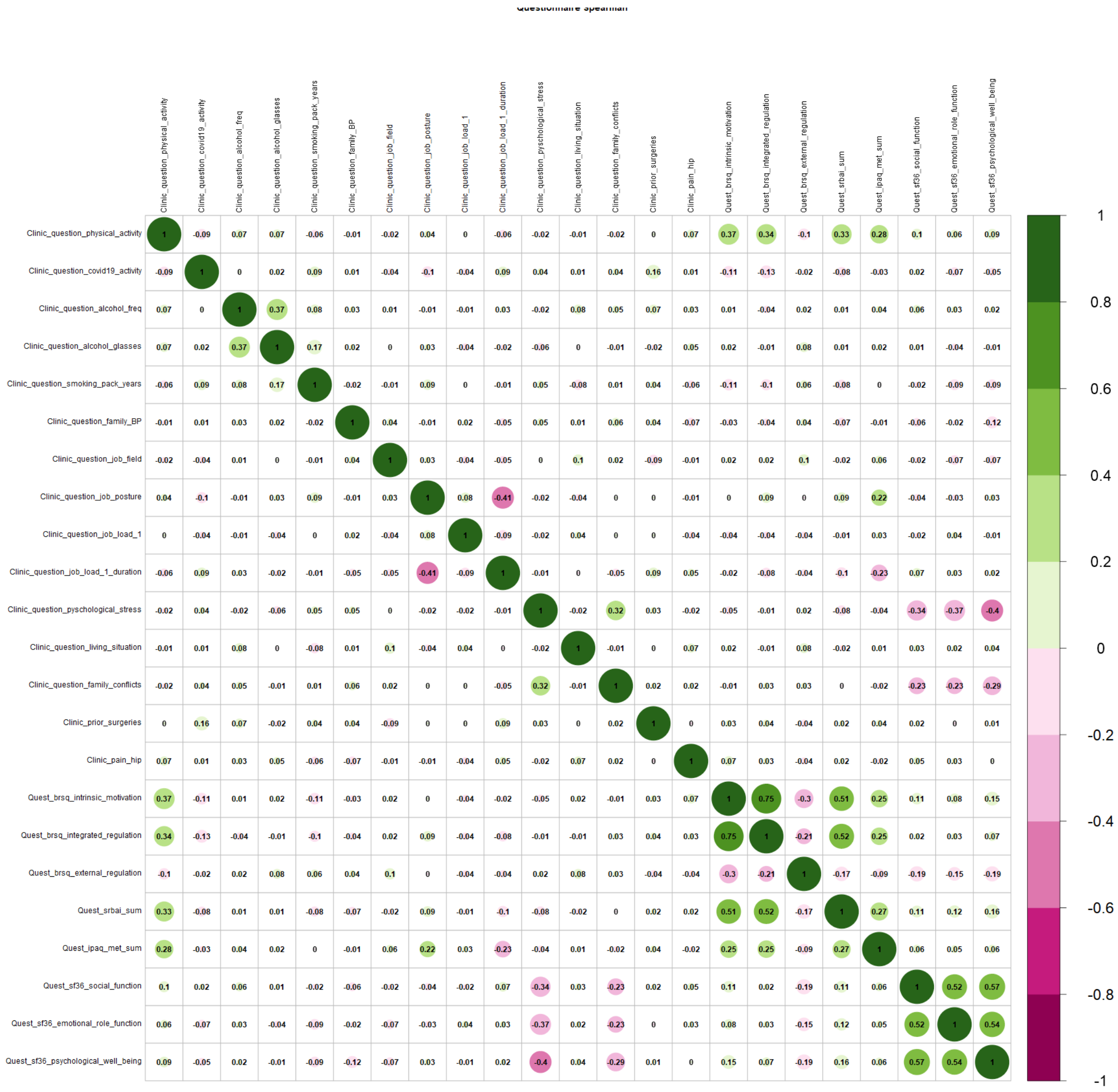

Supplementary Figure S2. Spearman correlation matrix of questionnaire dataset after removal of highly correlated variables

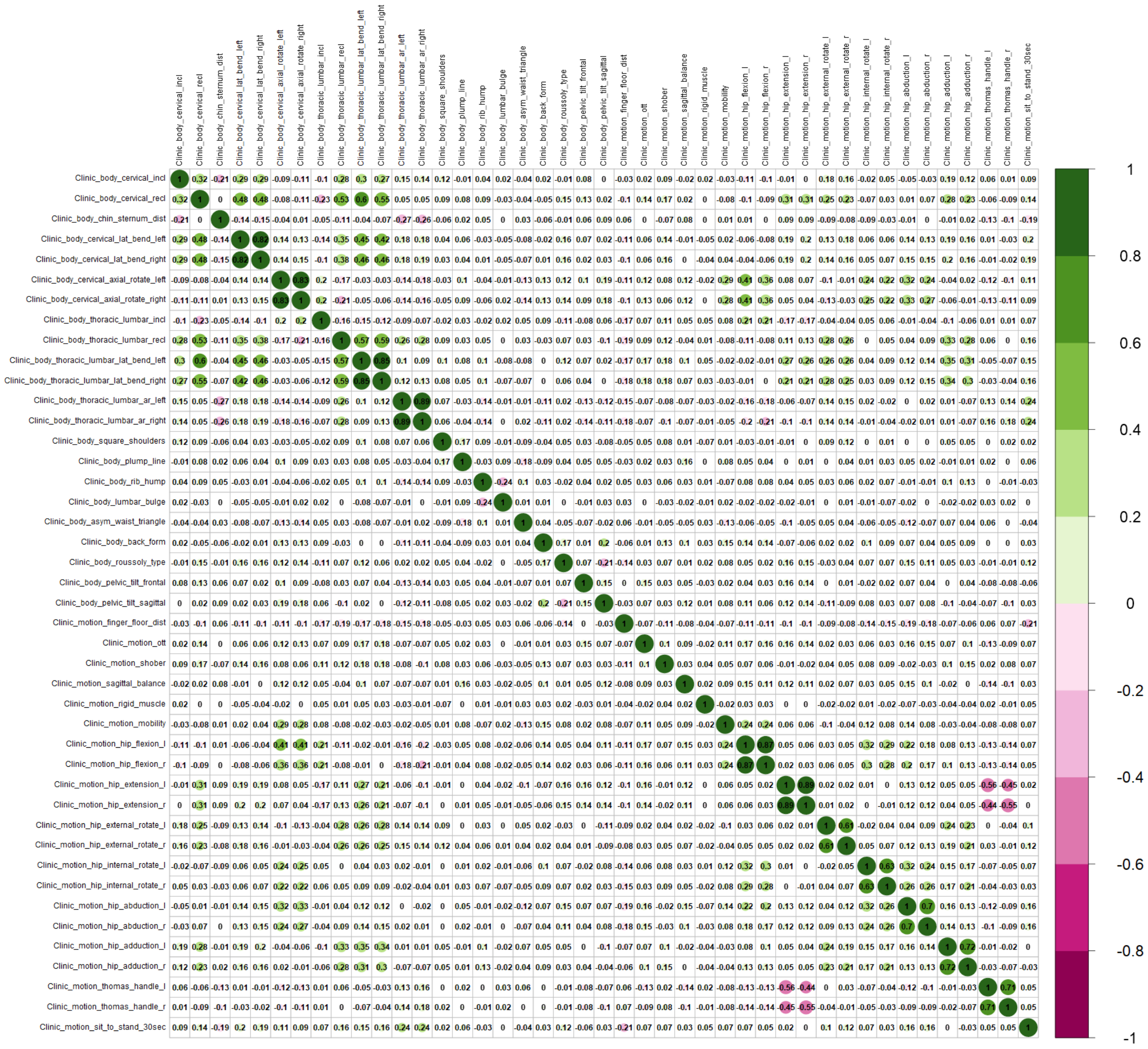

Supplementary Figure S3. Spearman correlation matrix of clinical assessment dataset

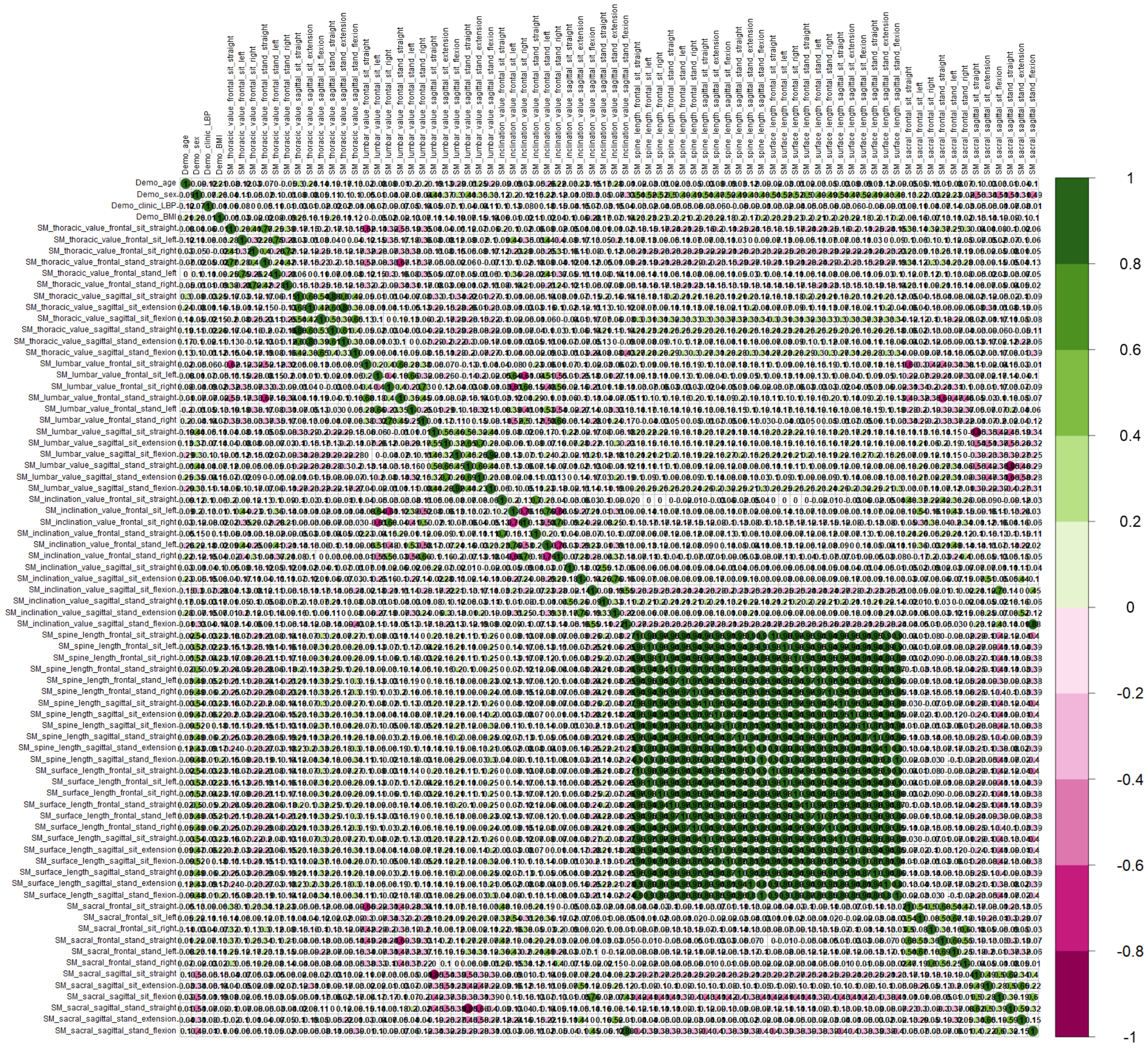

Supplementary Figure S4. Spearman correlation matrix of back shape and function dataset before removal of highly correlated variables

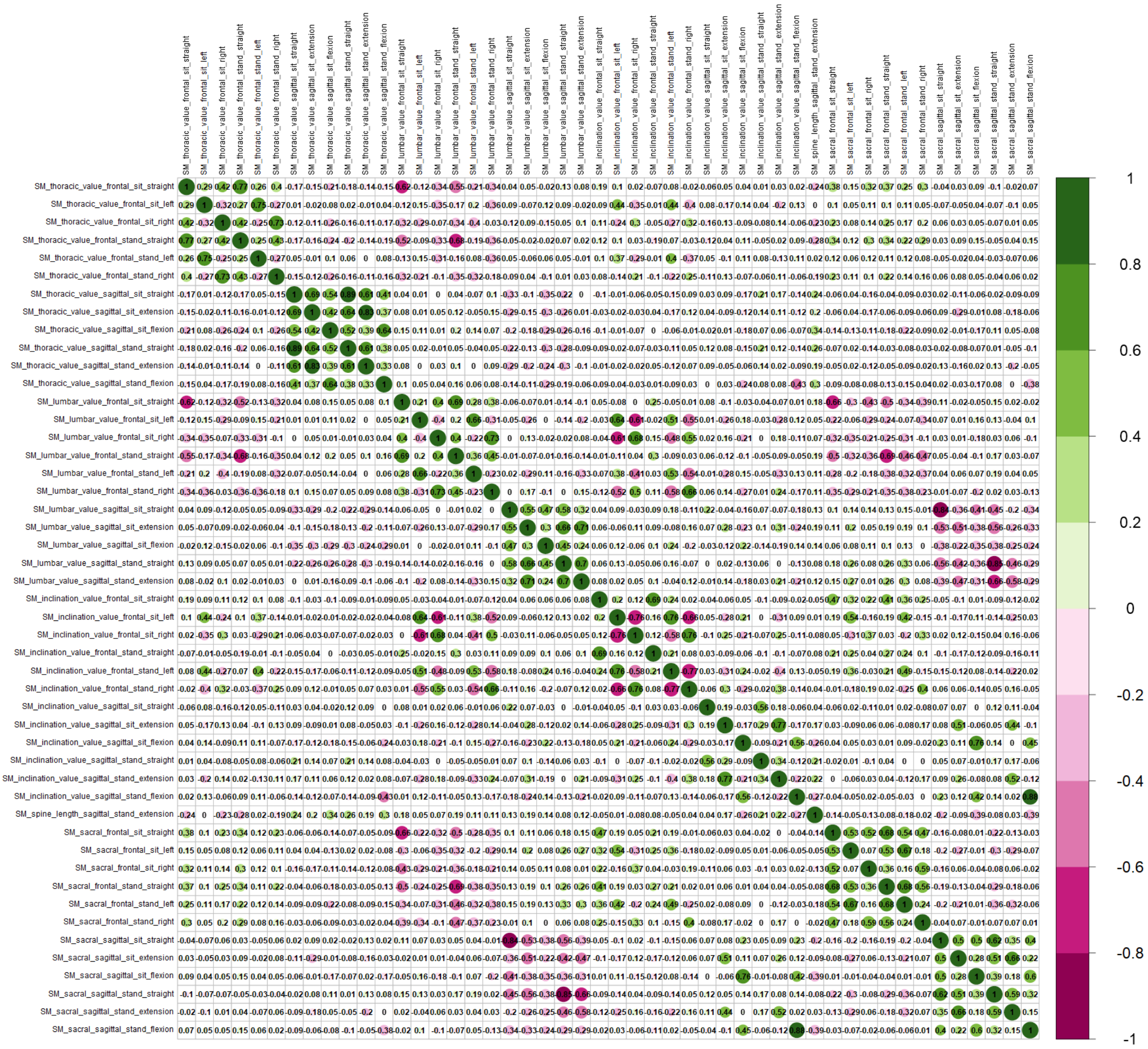

Supplementary Figure S5. Spearman correlation matrix of back shape and function dataset after removal of highly correlated variables

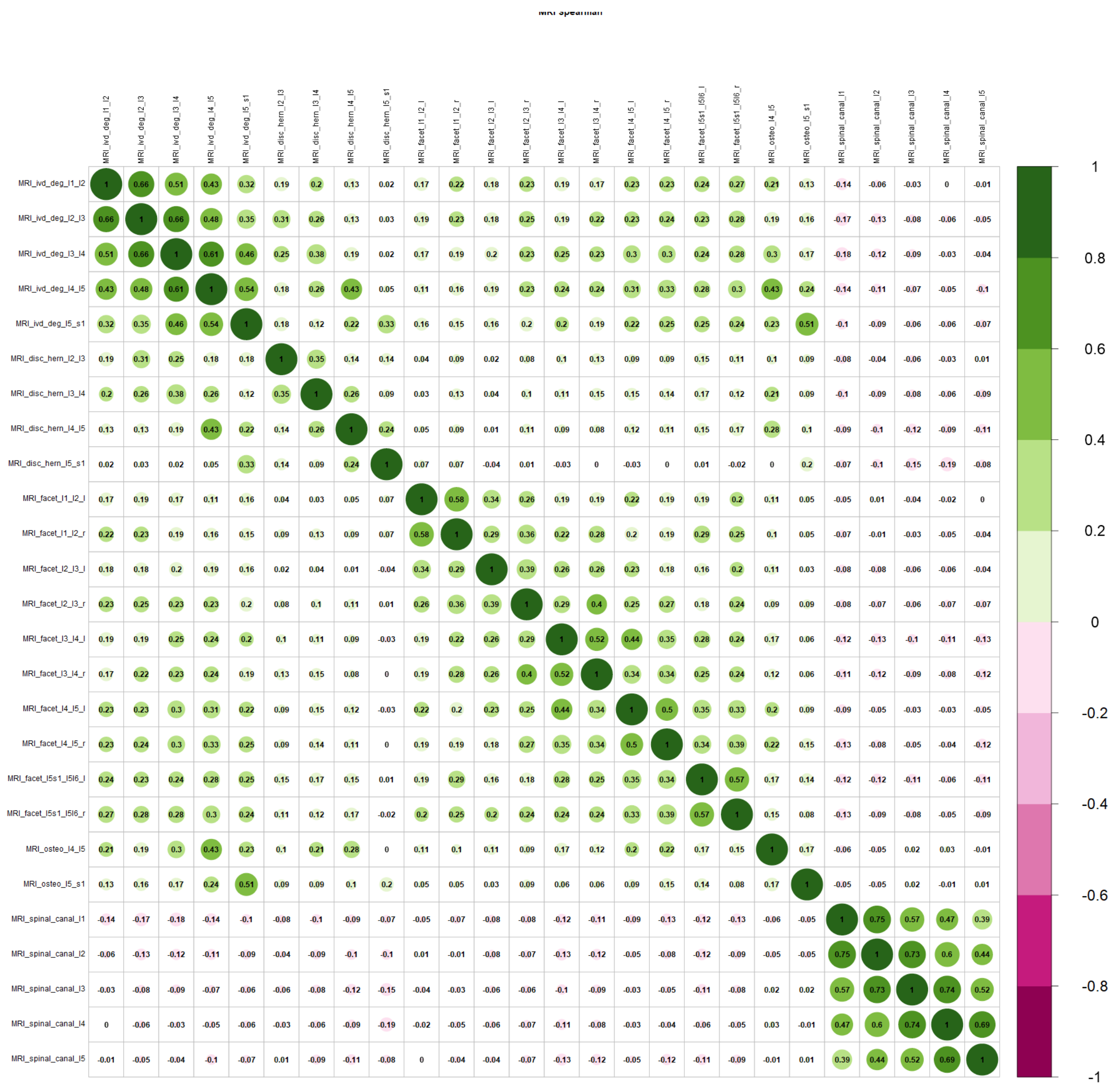

Supplementary Figure S6. Spearman correlation matrix of MRI dataset

Supplementary Data 3. Classification performance and feature reduction

Supplementary Table S22. Random forest classification performance.

Performance metrics based on classification using test sample across all 15 datasets using all features and boruta importance features. 95% Confidence interval (CI) is measured across 10 train test splits.

Modality	Method	Sample size	AUC [95% CI]	Accuracy [95% CI]	Sensitivity [95% CI]	Specificity [95% CI]	Feature Number [95% CI]
Q + C + M	boruta	507	0.699 [0.669 - 0.729]	0.709 [0.679 - 0.739]	0.622 [0.568 - 0.676]	0.778 [0.74 - 0.816]	23.8 [22.988 - 24.612]
C + M	boruta	622	0.679 [0.633 - 0.725]	0.683 [0.638 - 0.728]	0.593 [0.532 - 0.654]	0.762 [0.721 - 0.803]	24.9 [23.92 - 25.88]
Q + C + S + M	all	492	0.676 [0.628 - 0.724]	0.695 [0.65 - 0.74]	0.508 [0.417 - 0.599]	0.84 [0.799 - 0.881]	144 [144 - 144]
Q + M	boruta	545	0.674 [0.623 - 0.725]	0.689 [0.638 - 0.74]	0.585 [0.51 - 0.66]	0.766 [0.699 - 0.833]	15.8 [14.988 - 16.612]
Q + C	boruta	819	0.674 [0.639 - 0.709]	0.684 [0.65 - 0.718]	0.573 [0.511 - 0.635]	0.778 [0.743 - 0.813]	19 [18.416 - 19.584]
Q + C + M	all	507	0.672 [0.634 - 0.71]	0.686 [0.65 - 0.722]	0.555 [0.485 - 0.625]	0.791 [0.748 - 0.834]	96 [96 - 96]
Q + M	all	545	0.671 [0.623 - 0.719]	0.688 [0.638 - 0.738]	0.54 [0.486 - 0.594]	0.799 [0.736 - 0.862]	53 [53 - 53]
Q + C + S	all	776	0.671 [0.638 - 0.704]	0.684 [0.651 - 0.717]	0.531 [0.494 - 0.568]	0.809 [0.765 - 0.853]	118 [118 - 118]
C + M	all	622	0.67 [0.62 - 0.72]	0.678 [0.63 - 0.726]	0.568 [0.502 - 0.634]	0.769 [0.717 - 0.821]	73 [73 - 73]
Q + C + S	boruta	776	0.67 [0.633 - 0.707]	0.68 [0.64 - 0.72]	0.569 [0.526 - 0.612]	0.769 [0.72 - 0.818]	22.8 [21.547 - 24.053]
Q + C + S + M	boruta	492	0.667 [0.629 - 0.705]	0.675 [0.639 - 0.711]	0.585 [0.521 - 0.649]	0.749 [0.698 - 0.8]	24.3 [22.992 - 25.608]
C + S + M	all	607	0.665 [0.624 - 0.706]	0.677 [0.637 - 0.717]	0.552 [0.49 - 0.614]	0.783 [0.739 - 0.827]	121 [121 - 121]
Q + C	all	819	0.658 [0.637 - 0.679]	0.669 [0.648 - 0.69]	0.55 [0.503 - 0.597]	0.767 [0.716 - 0.818]	70 [70 - 70]
S + M	boruta	666	0.652 [0.628 - 0.676]	0.669 [0.645 - 0.693]	0.522 [0.471 - 0.573]	0.782 [0.743 - 0.821]	20.1 [19.01 - 21.19]
C + S + M	boruta	607	0.65 [0.605 - 0.695]	0.66 [0.617 - 0.703]	0.552 [0.472 - 0.632]	0.752 [0.697 - 0.807]	23.8 [21.304 - 26.296]
Q + S + M	all	528	0.648 [0.586 - 0.71]	0.674 [0.615 - 0.733]	0.474 [0.367 - 0.581]	0.824 [0.767 - 0.881]	101 [101 - 101]
Q + S + M	boruta	528	0.646 [0.585 - 0.707]	0.665 [0.607 - 0.723]	0.512 [0.431 - 0.593]	0.782 [0.72 - 0.844]	17.1 [14.877 - 19.323]

S + M	all	666	0.646 [0.619 - 0.673]	0.668 [0.641 - 0.695]	0.473 [0.407 - 0.539]	0.819 [0.766 - 0.872]	78 [78 - 78]
M	boruta	683	0.645 [0.618 - 0.672]	0.657 [0.636 - 0.678]	0.549 [0.468 - 0.63]	0.74 [0.701 - 0.779]	15.9 [14.863 - 16.937]
Q + S	all	823	0.645 [0.604 - 0.686]	0.662 [0.621 - 0.703]	0.493 [0.431 - 0.555]	0.798 [0.748 - 0.848]	75 [75 - 75]
C + S	all	920	0.644 [0.615 - 0.673]	0.658 [0.628 - 0.688]	0.493 [0.446 - 0.54]	0.796 [0.76 - 0.832]	95 [95 - 95]
M	all	683	0.637 [0.599 - 0.675]	0.65 [0.615 - 0.685]	0.533 [0.438 - 0.628]	0.741 [0.679 - 0.803]	30 [30 - 30]
Q	boruta	870	0.631 [0.61 - 0.652]	0.641 [0.617 - 0.665]	0.529 [0.481 - 0.577]	0.732 [0.69 - 0.774]	7.8 [7.061 - 8.539]
C + S	boruta	920	0.623 [0.594 - 0.652]	0.634 [0.607 - 0.661]	0.504 [0.45 - 0.558]	0.742 [0.712 - 0.772]	19 [17.348 - 20.652]
Q	all	870	0.623 [0.596 - 0.65]	0.638 [0.612 - 0.664]	0.489 [0.434 - 0.544]	0.758 [0.708 - 0.808]	27 [27 - 27]
C	all	964	0.618 [0.586 - 0.65]	0.628 [0.596 - 0.66]	0.498 [0.439 - 0.557]	0.737 [0.673 - 0.801]	47 [47 - 47]
Q + S	boruta	823	0.617 [0.577 - 0.657]	0.631 [0.592 - 0.67]	0.488 [0.423 - 0.553]	0.747 [0.697 - 0.797]	14.7 [13.871 - 15.529]
C	boruta	964	0.61 [0.577 - 0.643]	0.62 [0.588 - 0.652]	0.499 [0.437 - 0.561]	0.722 [0.675 - 0.769]	15.4 [13.674 - 17.126]
S	all	997	0.581 [0.549 - 0.613]	0.6 [0.569 - 0.631]	0.402 [0.345 - 0.459]	0.76 [0.726 - 0.794]	52 [52 - 52]
S	boruta	997	0.569 [0.538 - 0.6]	0.587 [0.557 - 0.617]	0.417 [0.373 - 0.461]	0.723 [0.69 - 0.756]	11.5 [9.909 - 13.091]

CI - Confidence interval, Q - Questionnaire, C - Clincal physical assessment, M - Spino-pelvic MRI, S - Back shape and function

Supplementary Table S23. Random forest classification performance (Imputaion).

Modality	Method	Sample Size	AUC [95% CI]	Accuracy [95% CI]	Sensitivity [95% CI]	Specificity [95% CI]	Feature Number [95% CI]
Q + C + M	boruta	1045	0.673 [0.654 - 0.692]	0.685 [0.664 - 0.706]	0.556 [0.526 - 0.586]	0.788 [0.742 - 0.834]	24.6 [23.471 - 25.729]

Q + C	boruta	1045	0.668 [0.635 - 0.701]	0.682 [0.65 - 0.714]	0.537 [0.47 - 0.604]	0.796 [0.748 - 0.844]	20.7 [19.228 - 22.172]
Q + C + S + M	all	1045	0.662 [0.625 - 0.699]	0.682 [0.645 - 0.719]	0.513 [0.46 - 0.566]	0.817 [0.77 - 0.864]	154 [154 - 154]
Q + C + S + M	boruta	1045	0.661 [0.627 - 0.695]	0.677 [0.643 - 0.711]	0.531 [0.483 - 0.579]	0.791 [0.745 - 0.837]	28 [26.455 - 29.545]
Q + C + M	all	1045	0.659 [0.629 - 0.689]	0.675 [0.645 - 0.705]	0.524 [0.464 - 0.584]	0.793 [0.741 - 0.845]	106 [106 - 106]
Q + C	all	1045	0.658 [0.629 - 0.687]	0.672 [0.64 - 0.704]	0.526 [0.475 - 0.577]	0.786 [0.73 - 0.842]	74 [74 - 74]
Q + C + S	all	1045	0.656 [0.624 - 0.688]	0.675 [0.64 - 0.71]	0.488 [0.442 - 0.534]	0.824 [0.762 - 0.886]	122 [122 - 122]
Q + C + S	boruta	1045	0.654 [0.623 - 0.685]	0.668 [0.637 - 0.699]	0.534 [0.479 - 0.589]	0.777 [0.725 - 0.829]	25 [23.216 - 26.784]
Q + S + M	all	1045	0.648 [0.617 - 0.679]	0.666 [0.636 - 0.696]	0.484 [0.446 - 0.522]	0.812 [0.779 - 0.845]	108 [108 - 108]
C + S + M	all	1045	0.643 [0.606 - 0.68]	0.662 [0.624 - 0.7]	0.478 [0.429 - 0.527]	0.808 [0.759 - 0.857]	130 [130 - 130]
C + S + M	boruta	1045	0.641 [0.602 - 0.68]	0.655 [0.617 - 0.693]	0.516 [0.452 - 0.58]	0.768 [0.719 - 0.817]	24.1 [23.063 - 25.137]
C + M	all	1045	0.638 [0.62 - 0.656]	0.657 [0.637 - 0.677]	0.476 [0.44 - 0.512]	0.8 [0.755 - 0.845]	82 [82 - 82]
Q + S + M	boruta	1045	0.638 [0.615 - 0.661]	0.656 [0.634 - 0.678]	0.484 [0.448 - 0.52]	0.791 [0.763 - 0.819]	21.9 [20.71 - 23.09]
C	boruta	1045	0.637 [0.598 - 0.676]	0.653 [0.613 - 0.693]	0.494 [0.434 - 0.554]	0.781 [0.724 - 0.838]	15 [14.416 - 15.584]
C + S	all	1045	0.635 [0.595 - 0.675]	0.655 [0.615 - 0.695]	0.452 [0.383 - 0.521]	0.816 [0.761 - 0.871]	98 [98 - 98]
C + S	boruta	1045	0.633 [0.591 - 0.675]	0.648 [0.606 - 0.69]	0.5 [0.433 - 0.567]	0.765 [0.701 - 0.829]	19.9 [18.375 - 21.425]
C	all	1045	0.631 [0.597 - 0.665]	0.65 [0.616 - 0.684]	0.469 [0.418 - 0.52]	0.794 [0.74 - 0.848]	50 [50 - 50]
Q + M	boruta	1045	0.631 [0.592 - 0.67]	0.646 [0.612 - 0.68]	0.494 [0.417 - 0.571]	0.77 [0.727 - 0.813]	16.5 [15.32 - 17.68]

Q + S	all	1045	0.631 [0.6 - 0.662]	0.651 [0.619 - 0.683]	0.447 [0.41 - 0.484]	0.811 [0.767 - 0.855]	76 [76 - 76]
C + M	boruta	1045	0.63 [0.592 - 0.668]	0.645 [0.609 - 0.681]	0.498 [0.436 - 0.56]	0.763 [0.719 - 0.807]	17.8 [16.299 - 19.301]
Q	boruta	1045	0.627 [0.588 - 0.666]	0.641 [0.604 - 0.678]	0.507 [0.459 - 0.555]	0.747 [0.693 - 0.801]	9.6 [8.695 - 10.505]
Q	all	1045	0.616 [0.591 - 0.641]	0.631 [0.607 - 0.655]	0.482 [0.429 - 0.535]	0.747 [0.715 - 0.779]	28 [28 - 28]
Q + S	boruta	1045	0.616 [0.589 - 0.643]	0.631 [0.604 - 0.658]	0.473 [0.426 - 0.52]	0.756 [0.717 - 0.795]	19.1 [17.818 - 20.382]
S + M	all	1045	0.613 [0.57 - 0.656]	0.636 [0.594 - 0.678]	0.418 [0.36 - 0.476]	0.808 [0.771 - 0.845]	84 [84 - 84]
Q + M	all	1045	0.612 [0.589 - 0.635]	0.631 [0.609 - 0.653]	0.453 [0.391 - 0.515]	0.771 [0.724 - 0.818]	60 [60 - 60]
S + M	boruta	1045	0.606 [0.579 - 0.633]	0.624 [0.598 - 0.65]	0.44 [0.396 - 0.484]	0.772 [0.732 - 0.812]	16.7 [15.349 - 18.051]
S	boruta	1045	0.596 [0.557 - 0.635]	0.614 [0.576 - 0.652]	0.437 [0.386 - 0.488]	0.754 [0.715 - 0.793]	14 [12.314 - 15.686]
S	all	1045	0.583 [0.55 - 0.616]	0.608 [0.575 - 0.641]	0.368 [0.318 - 0.418]	0.798 [0.751 - 0.845]	52 [52 - 52]
M	boruta	1045	0.58 [0.547 - 0.613]	0.61 [0.576 - 0.644]	0.346 [0.293 - 0.399]	0.817 [0.761 - 0.873]	18.1 [16.27 - 19.93]
M	all	1045	0.571 [0.543 - 0.599]	0.599 [0.569 - 0.629]	0.314 [0.266 - 0.362]	0.826 [0.775 - 0.877]	36 [36 - 36]

CI - Confidence interval, Q - Questionnaire, C - Clincal physical assessment, M - Spino-pelvic MRI, S - Back shape and function

Supplementary Table S24. Boruta selected important features in the questionnaire modality dataset.

Modality	Assessment	Selected (%)	Importance (mean)
questionnaire	SF-36 social function	100	21.42
questionnaire	SF-36 psychological well being	100	20.02
questionnaire	hip pain	100	15
questionnaire	smoking pack years	100	11.8
questionnaire	family back pain	100	7.27
demographic	age	80	6.15
questionnaire	physical activity 150min/week	70	6.04

questionnaire	job load	70	5.25
questionnaire	living situation	10	4.59
questionnaire	BRSQ intrinsic motivation	30	4.53
questionnaire	BRSQ integrated regulation	20	3.86

BRSQ - Behavioural Regulation in Sport Questionnaire, SF-36 - Short-form 36 Health Status Questionnaire.

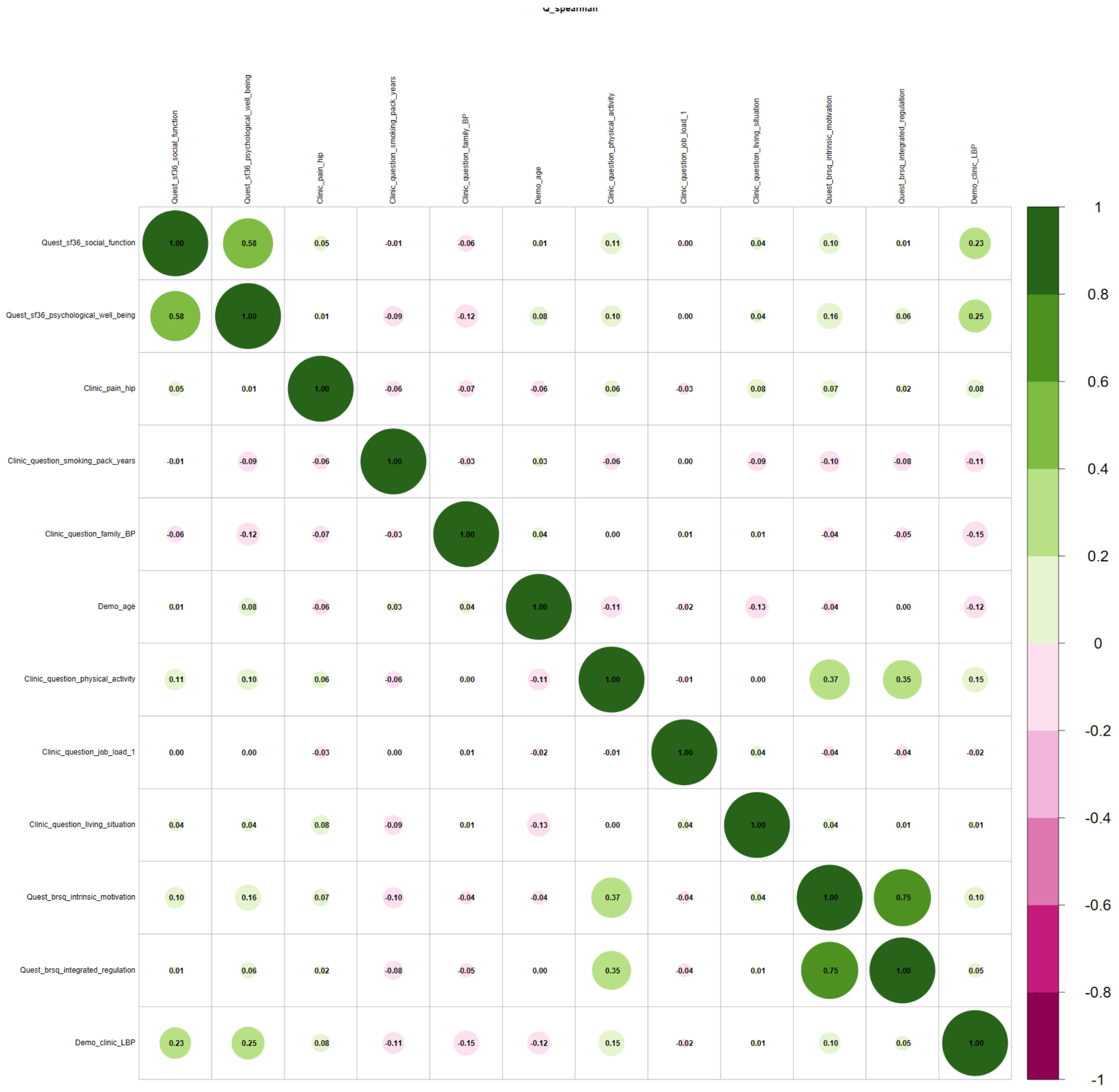

Supplementary Figure S7. Spearman correlation matrix of Boruta selected important features for questionnaire dataset

Supplementary Table S25. Boruta selected important features for clinical physical assessment dataset

Modality	Variable	Selected (%)	Importance (mean)
Clinical	cervical axial rotate left	100	14.19
Clinical	sit to stand 30sec	100	13.61
Clinical	general mobility	100	13.39
Clinical	rigid muscle	100	11.22
Clinical	cervical axial rotate right	100	7.82
Clinical	hip flexion left	100	6.29
Demographic	age	100	5.61
Clinical	hip flexion right	90	5.53
Clinical	hip abduction left	90	5.5
Clinical	hip internal rotate right	60	5.17
Clinical	shober	50	4.68
Clinical	chin sternum distance	80	4.58
Clinical	thoracic lumbar lateral bend right	70	4.42
Clinical	cervical lateral bend left	60	4.35
Clinical	cervical inclination	10	4.33
Clinical	hip internal rotate left	40	4.29
Clinical	back form	90	4.25
Clinical	hip extension right	10	4.16
Clinical	thoracic lumbar inclination	30	4.08
Clinical	thomas handle right	10	4.03
Clinical	hip abduction right	70	3.99
Clinical	lumbar bulge	10	3.81
Demographic	sex	10	3.76
Clinical	roussoly type	10	3.67
Clinical	cervical reclination	40	3.59
Clinical	asymmetrical waist triangle	10	3.42

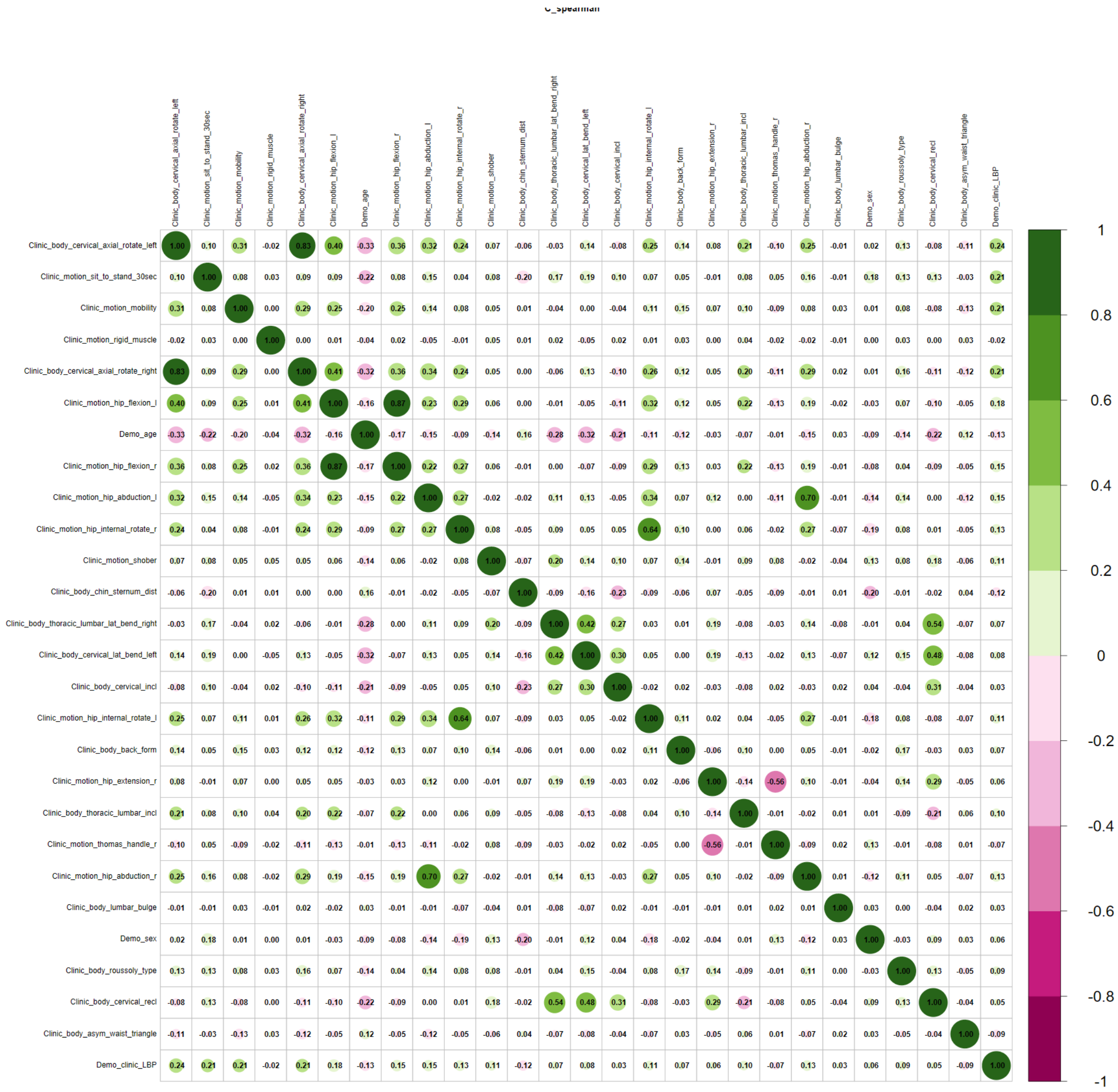

Supplementary Figure S8. Spearman correlation matrix of Boruta selected important features for clinical assessment dataset

Supplementary Table S26. Boruta selected important features for superficial spine morphology modality dataset

Modality	Variable	selected (%)	Importance (mean)
Demographic	age	100	7.8
Back shape & function	sacral frontal stand left	100	6.51
Back shape & function	inclination value frontal stand left	100	6.26
Back shape & function	inclination value sagittal sit extension	80	5.22
Back shape & function	thoracic value sagittal stand extension	90	4.96

Back shape & function	thoracic value frontal stand left	70	4.86
Back shape & function	lumbar value frontal stand right	100	4.81
Back shape & function	sacral frontal sit left	70	4.78
Back shape & function	inclination value sagittal stand extension	30	4.38
Back shape & function	lumbar value sagittal sit flexion	80	4.19
Back shape & function	spine length sagittal stand extension	70	4.15
Back shape & function	inclination value frontal stand right	70	3.92
Back shape & function	thoracic value frontal sit left	70	3.92
Back shape & function	inclination value frontal sit left	60	3.9
Back shape & function	sacral frontal stand straight	10	3.66
Back shape & function	lumbar value sagittal sit extension	40	3.48
Back shape & function	lumbar value sagittal stand extension	10	3.46

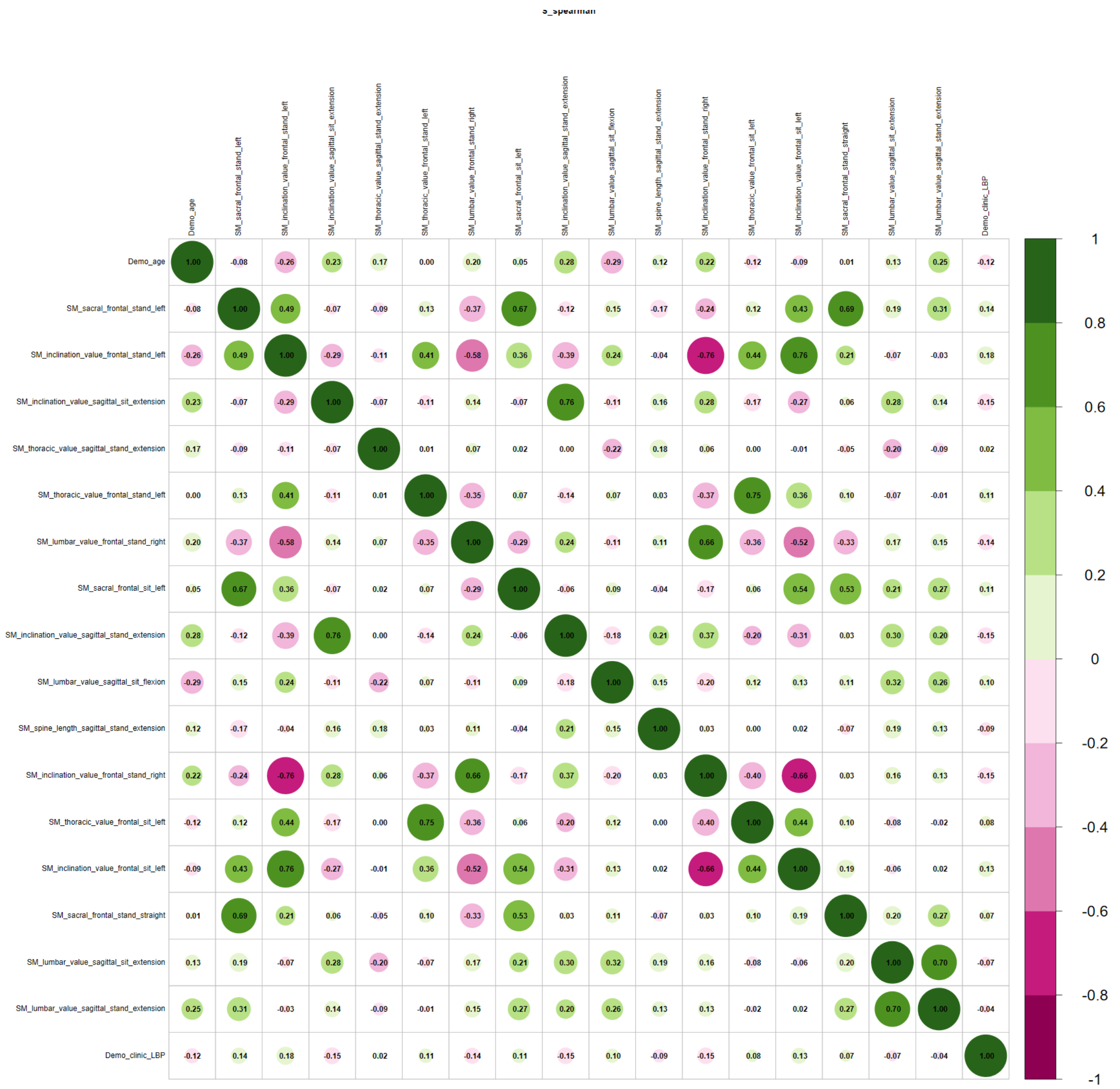

Supplementary Figure S9. Spearman correlation matrix of Boruta selected important features for back shape and function dataset

Supplementary Table S27. Boruta selected important features for MRI modality dataset

Modality	Assessment	Selected (%)	Importance (mean)
MRI	IVD herniation L4 - L5	100	19.49
MRI	spinal canal width L2	100	9.94
MRI	IVD degeneration L3 - L4	100	9.46
MRI	IVD degeneration L4 - L5	100	9.14
MRI	spinal canal width L1	100	8.13
MRI	osteochondrosis L4 - L5	100	8.12
MRI	IVD herniation L5 - S1	100	7.38
MRI	IVD degeneration L2 - L3	100	6.85
MRI	facet joint L3 - L4 right	100	6.81
MRI	facet joint L5/S1 - L5/L6 right	100	6.56
MRI	facet joint L3 - L4 left	90	5.53
Demographic	age	30	5.29
MRI	facet joint L1 - L2 right	30	5.12
MRI	IVD degeneration L5 - S1	80	4.97
MRI	osteochondrosis L5 - S1	60	4.66
MRI	IVD degeneration L1 - L2	100	4.64
MRI	facet joint L1 - L2 left	20	4.59
MRI	facet joint L5/S1 - L5/L6 left	80	4.34
MRI	IVD herniation L2 - L3	60	4.16
MRI	IVD herniation L3 - L4	30	4.08
MRI	spinal canal width L4	10	3.77

Boruta selected important variables in MRI modality dataset across ten-fold feature selection.
IVD – intervertebral disc

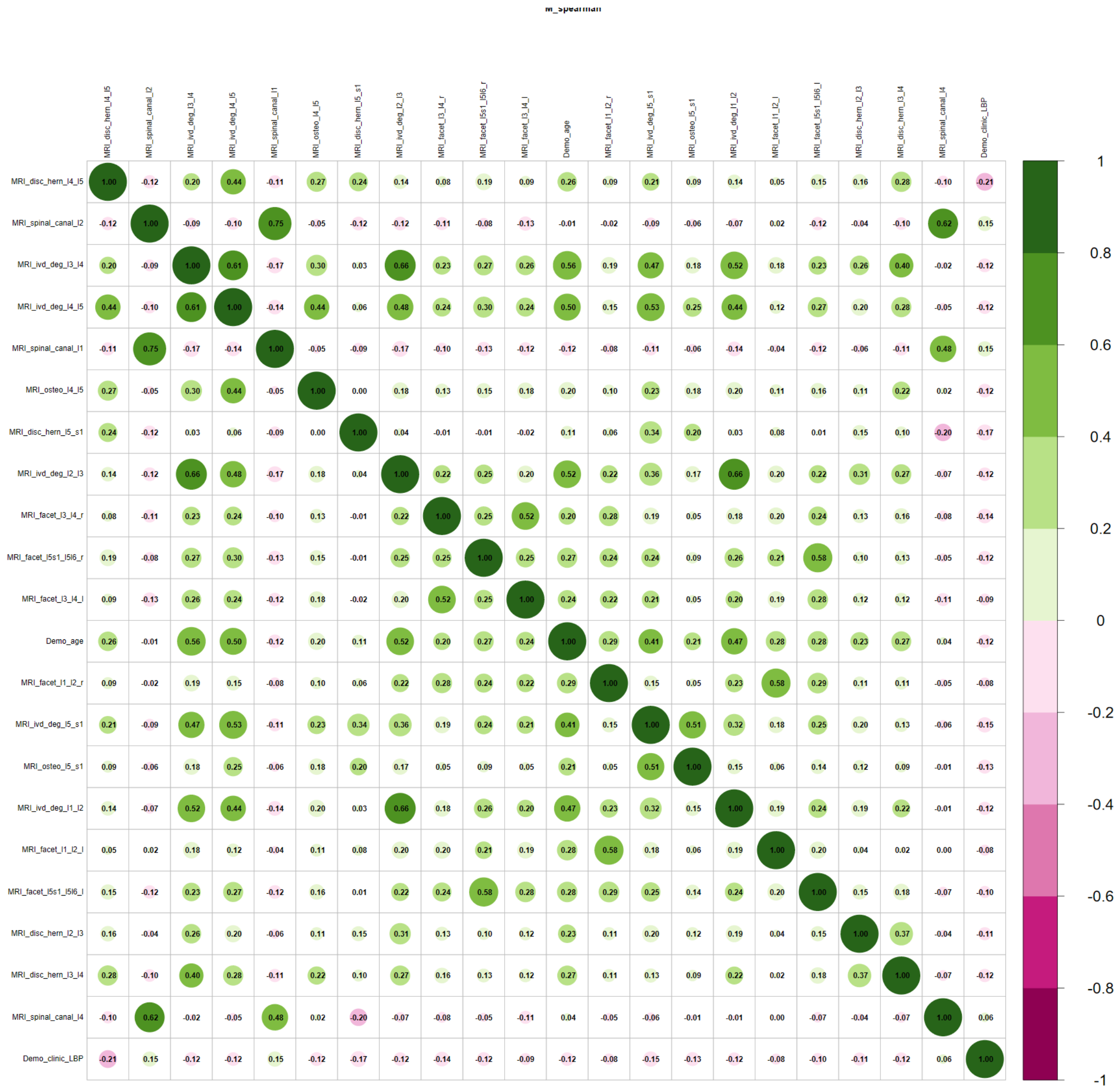

Supplementary Figure S10. Spearman correlation matrix of Boruta selected important features for MRI dataset

Supplementary Table S28. Boruta selected important features for questionnaire and clinical physical assessment dataset

Modality	Variable	selected (%)	Importance (mean)
Questionnaire	SF-36 psychological well being	100	17.04
Questionnaire	SF-36 social function	100	15.02
Clinical	Hip pain	100	12.39
Clinical	general mobility	100	11.39
Clinical	cervical axial rotate left	100	10.62

Clinical	sit to stand 30sec	100	9.78
Questionnaire	smoking pack years	100	9.62
Clinical	rigid muscle	100	8.52
Questionnaire	family back pain	100	6.53
Clinical	hip abduction left	100	6
Questionnaire	physical activity 150min/week	100	5.88
Demographic	age	100	5.45
Clinical	cervical axial rotate right	100	5.42
Clinical	hip abduction right	90	5.23
Clinical	hip internal rotate right	80	5.02
Clinical	thoracic lumbar lat bend right	60	4.72
Clinical	back form	100	4.34
Clinical	hip flexion right	100	4.27
Clinical	shober	10	4.2
Clinical	square shoulders	10	4.05
Clinical	cervical lat bend left	30	4.04
Clinical	hip flexion left	70	3.76
Questionnaire	BRSQ intrinsic motivation	20	3.63
Clinical	finger floor distance	10	3.59
Questionnaire	job load 1	10	3.48
Clinical	hip adduction right	10	3.34

BRSQ - Behavioural Regulation in Sport Questionnaire, SF-36 - Short-form 36 Health Status Questionnaire.

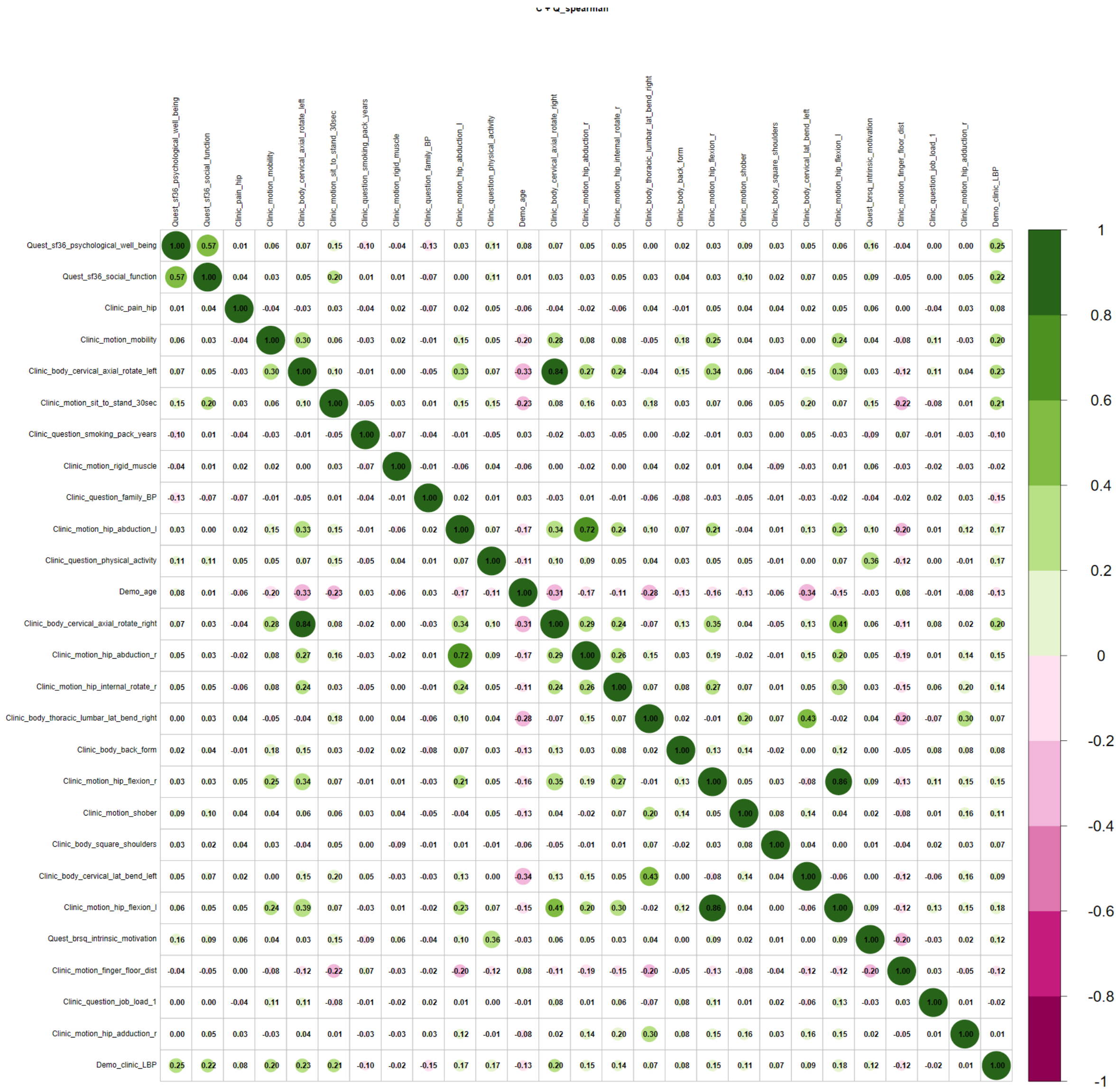

Supplementary Figure S11. Spearman correlation matrix of Boruta selected important features for questionnaire + clinic dataset

Supplementary Table S29. Boruta selected important features for clinic physical assessment and back shape and function dataset

Modality	Variable	selected (%)	Importance (mean)
Clinical	cervical axial rotate left	100	14.2
Clinical	general mobility	100	11.83
Clinical	sit to stand 30sec	100	11.53
Clinical	rigid muscle	100	9.97
Clinical	cervical axial rotate right	100	7
Clinical	hip flexion left	100	5.99
Demogrpahic	age	100	5.82

Back shape & function	inclination value frontal stand left	90	5.31
Back shape & function	sacral frontal stand left	90	5.12
Back shape & function	thoracic value sagittal stand extension	90	5.04
Clinical	hip abduction left	90	4.7
Clinical	hip flexion right	100	4.68
Clinical	cervical lateral bend left	20	4.54
Clinical	back form	70	4.49
Back shape & function	lumbar value frontal sit straight	10	4.26
Back shape & function	inclination value sagittal stand extension	10	4.23
Back shape & function	sacral sagittal sit straight	10	4.11
Back shape & function	lumbar value frontal sit right	20	4.05
Back shape & function	lumbar value sagittal stand extension	10	3.97
Back shape & function	inclination value frontal stand right	60	3.95
Back shape & function	inclination value frontal sit left	40	3.91
Back shape & function	lumbar value frontal stand right	70	3.81
Clinical	thoracic lumbar inclination	40	3.8
Back shape & function	lumbar value frontal stand straight	30	3.79
Clinical	shober	20	3.79
Back shape & function	inclination value sagittal sit extension	30	3.78
Clinical	hip abduction right	30	3.74
Clinical	thoracic lumbar lateral bend right	60	3.72
Back shape & function	sacral frontal sit left	50	3.71
Back shape & function	sacral frontal sit straight	50	3.59
Back shape & function	thoracic value sagittal sit extension	20	3.58
Back shape & function	thoracic value frontal stand right	10	3.46
Back shape & function	thoracic value frontal stand left	40	3.44
Back shape & function	sacral sagittal stand straight	20	3.29
Clinical	chin sternum distance	10	3.29
Back shape & function	inclination value frontal sit right	10	3.21

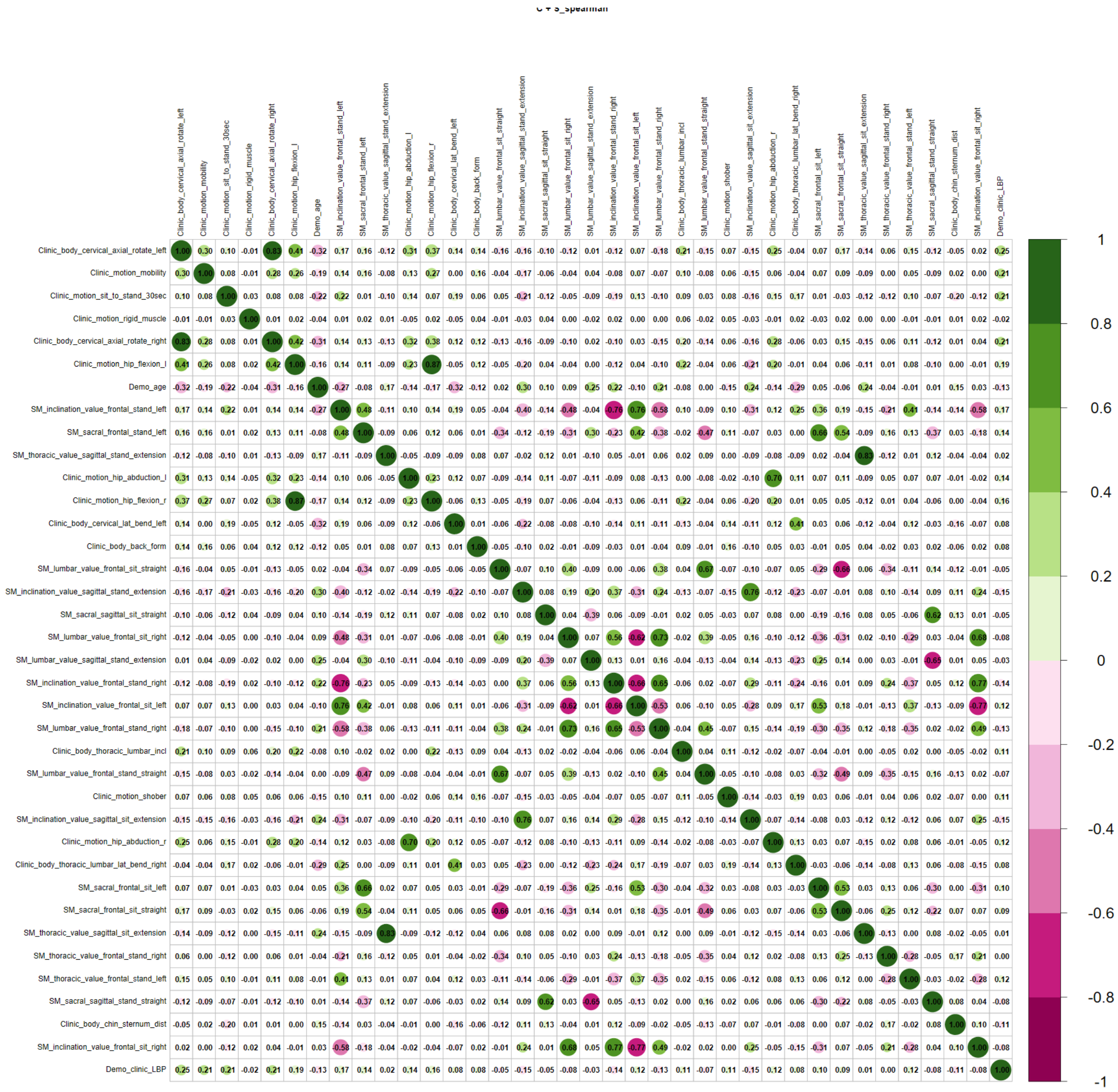

Supplementary Figure S12. Spearman correlation matrix of Boruta selected important features for clinic physical assessment and back shape and function dataset

Supplementary Table S30. Boruta selected important features for MRI and questionnaire dataset

Modality	Variable	selected (%)	Importance (mean)
Questionnaire	SF-36 social function	100	18.18
Questionnaire	SF-36 psychological well being	100	16.93
MRI	intervertebral disc herniation L4 - L5	100	14.05
MRI	spinal canal stenosis L2	100	11.4
MRI	intervertebral disc degeneration L2 - L3	100	9.11
Questionnaire	hip pain	100	8.53

MRI	spinal canal stenosis L1	100	8.05
MRI	intervertebral disc degeneration L4 - L5	100	7.75
MRI	intervertebral disc degeneration L5 - S1	100	6.86
MRI	osteochondrosis intervertebralis L4 - L5	20	6.16
MRI	intervertebral disc herniation L5 - S1	100	5.98
MRI	intervertebral disc degeneration L3 - L4	90	5.76
MRI	facet joint L3 - L4 right	100	5.37
MRI	facet joint L5/S1 - L5/L6 right	90	4.8
MRI	osteochondrosis intervertebralis L5 - S1	30	4.5
MRI	spinal canal stenosis L4	60	4.32
MRI	facet joint L4 - L5 right	20	4.16
MRI	intervertebral disc degeneration L1 - L2	30	4.08
Questionnaire	physical activity 150min/week	20	4.08
MRI	facet joint L3 - L4 left	50	4.06
Questionnaire	living situation	40	3.98
MRI	spinal canal stenosis L3	20	3.97
Questionnaire	SF-36 emotional role function	10	3.68

SF-36 - Short-form 36 Health Status Questionnaire

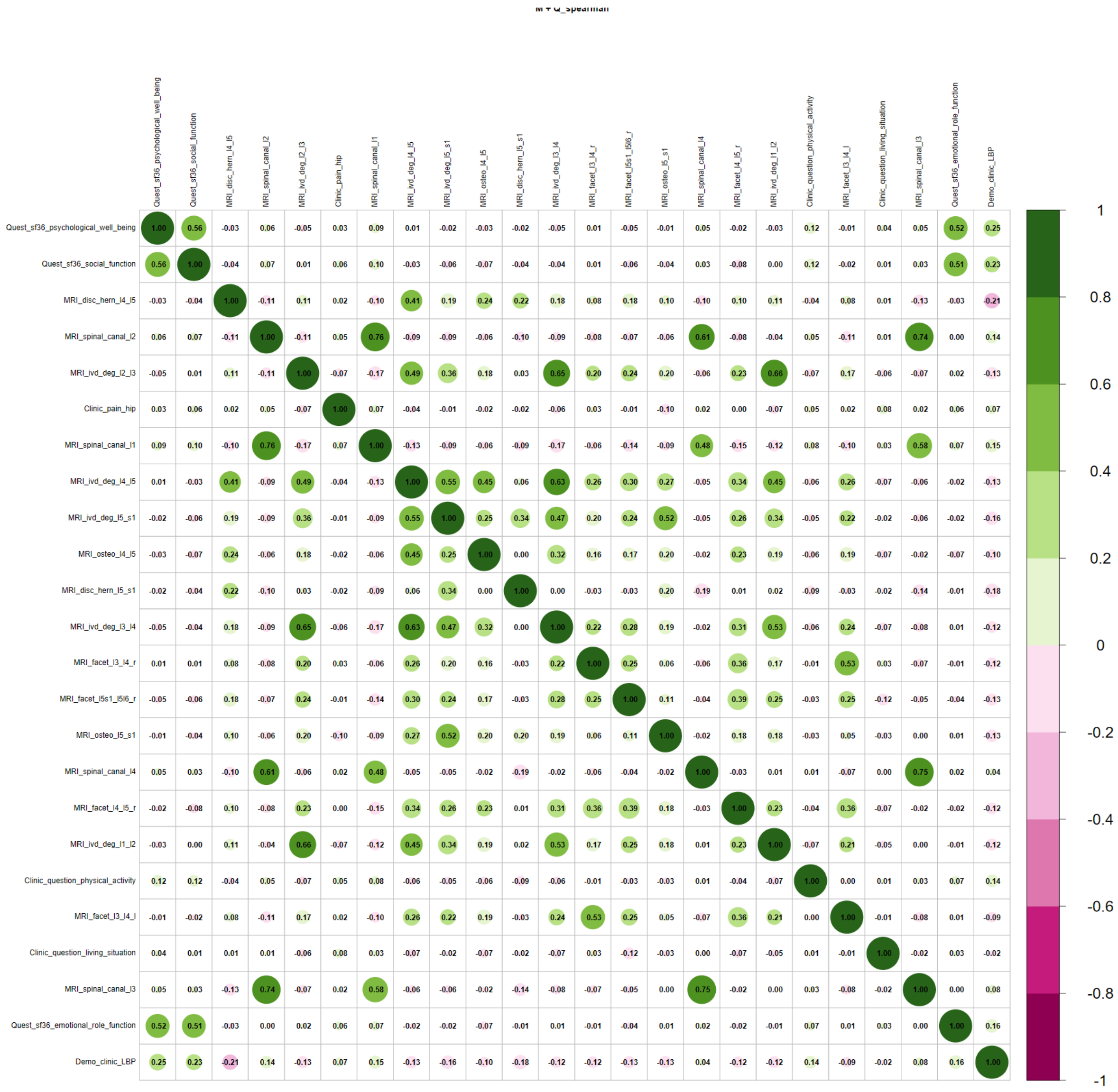

Supplementary Figure S13. Spearman correlation matrix of Boruta selected important features for questionnaire + MRI dataset

Supplementary Table S31. Boruta selected important features for MRI and clinical assessment modality dataset

**Modality**	**Assessment**	**Selected (%)**	**Importance (mean)**
MRI	IVD herniation L4 - L5	100	13.72
clinical examination	cervical axial rotate left	100	13.68
clinical examination	hip flexion left	100	11.01
clinical examination	general mobility	100	9.92
clinical examination	sit to stand 30sec	100	9.69

clinical examination	hip flexion right	100	9
MRI	spinal canal width L2	100	8.41
MRI	IVD degeneration L2 - L3	100	7.01
clinical examination	cervical axial rotate right	100	6.41
MRI	IVD degeneration L4 - L5	100	6.24
MRI	IVD degeneration L3 - L4	100	6.03
MRI	osteochondrosis L4 - L5	100	5.96
clinical examination	rigid muscle	100	5.66
MRI	IVD herniation L5 - S1	100	5.49
MRI	IVD degeneration L5 - S1	100	5.25
MRI	facet joint L5/S1 - L5/L6 right	90	5.05
clinical examination	chin sternum distance	90	4.99
clinical examination	hip internal rotate right	100	4.95
MRI	osteochondrosis L5 - S1	90	4.85
MRI	spinal canal width L1	100	4.72
clinical examination	hip internal rotate left	90	4.68
MRI	IVD degeneration L1 - L2	90	4.67
MRI	facet joint L5/S1 - L5/L6 left	70	4.47
clinical examination	hip abduction right	20	4.47
clinical examination	hip abduction left	30	4.3
MRI	facet joint L3 L4 right	60	4.11
Demographic	age	40	4.09
clinical examination	thoracic lumbar inclination	30	4.08
clinical examination	thoracic lumbar axial rotate left	10	3.87
clinical examination	asymmetrical waist triangle	40	3.72
MRI	IVD herniation L3 - L4	10	3.51
MRI	facet joint L3 - L4 left	20	3.48
clinical examination	back form	10	3.42

IVD – intervertebral disc

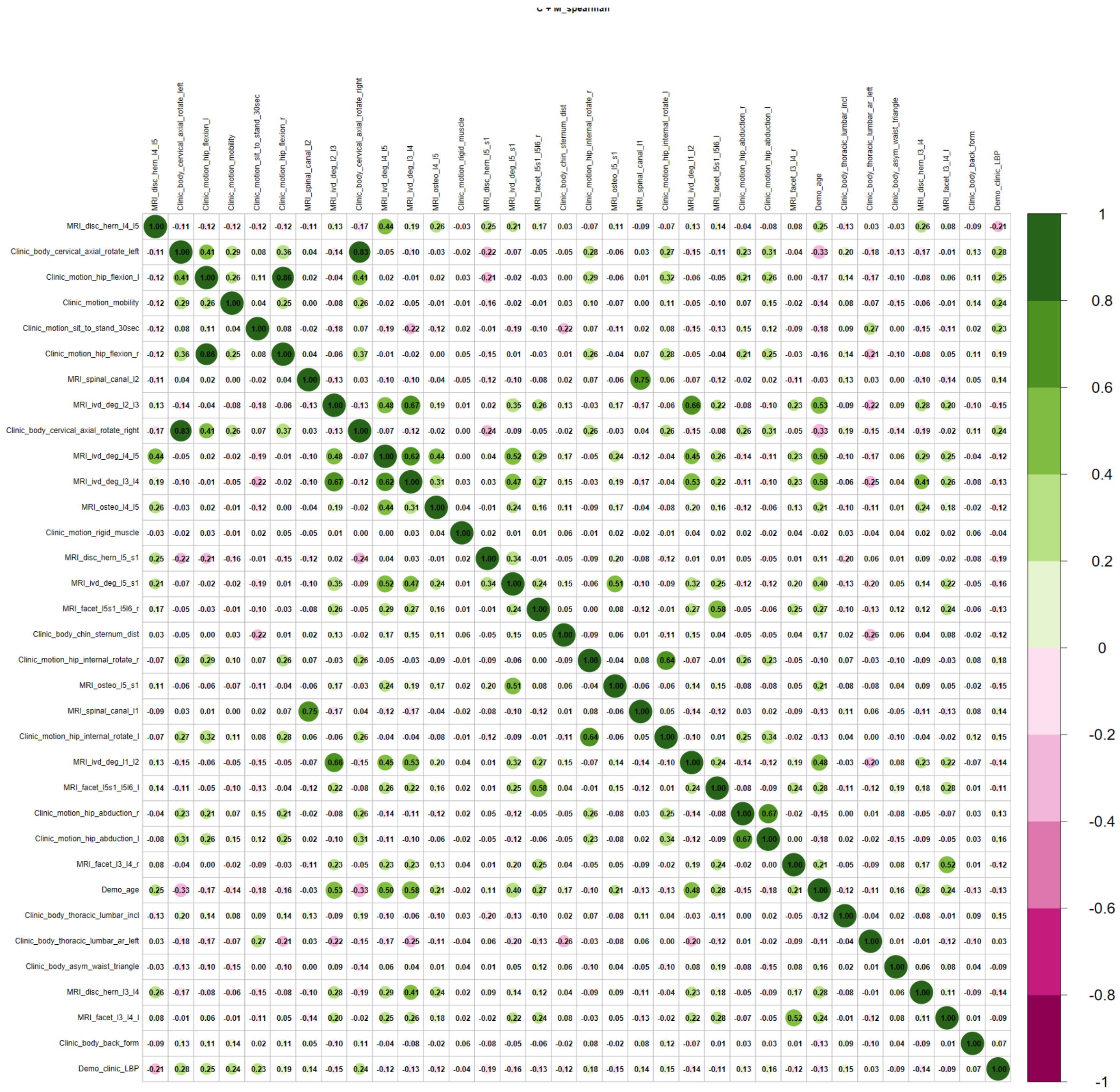

Supplementary Figure S14. Spearman correlation matrix of Boruta selected important features for clinical assessment + MRI dataset

Supplementary Table S32. Boruta selected important features for MRI and superficial spine morphology dataset

Modality	Variable	selected (%)	Importance (mean)
MRI	intervertebral disc herniation L4 - L5	100	16.13
MRI	spinal canal stenosis L2	100	9.18
MRI	intervertebral disc degeneration L4 - L5	100	8.14
MRI	intervertebral disc degeneration L3 - L4	100	7.25
MRI	osteochondrosis intervertebralis L4 - L5	100	7.09

MRI	spinal canal stenosis L1	100	6.45
MRI	intervertebral disc degeneration L2 - L3	100	6.08
Back shape & function	spine length sagittal stand extension	100	5.42
MRI	intervertebral disc herniation L5 - S1	100	5.25
Back shape & function	inclination value sagittal sit extension	90	5.03
Back shape & function	inclination value sagittal stand extension	80	4.78
MRI	intervertebral disc degeneration L5 - S1	80	4.76
Back shape & function	inclination value frontal stand right	90	4.65
MRI	facet joint L3 - L4 right	80	4.54
Back shape & function	inclination value sagittal sit flexion	10	4.47
Back shape & function	lumbar value frontal stand left	50	4.43
Demographic	age	70	4.39
Back shape & function	sacral frontal stand left	40	4.39
Back shape & function	inclination value frontal stand left	50	4.37
Back shape & function	thoracic value sagittal stand extension	40	4.3
Back shape & function	lumbar value sagittal sit extension	60	4.29
Back shape & function	lumbar value frontal stand right	60	4.23
MRI	osteochondrosis intervertebralis L5 - S1	70	4.18
Back shape & function	lumbar value frontal stand straight	40	4.13
MRI	intervertebral disc degeneration L1 - L2	20	4.06
Back shape & function	thoracic value frontal sit left	40	3.95
MRI	intervertebral disc herniation L2 - L3	60	3.85
MRI	facet joint L5/S1 - L5/L6 right	30	3.81
Back shape & function	lumbar value sagittal sit flexion	30	3.62
Back shape & function	sacral frontal sit left	10	3.36
MRI	facet joint L3 - L4 left	10	3.32

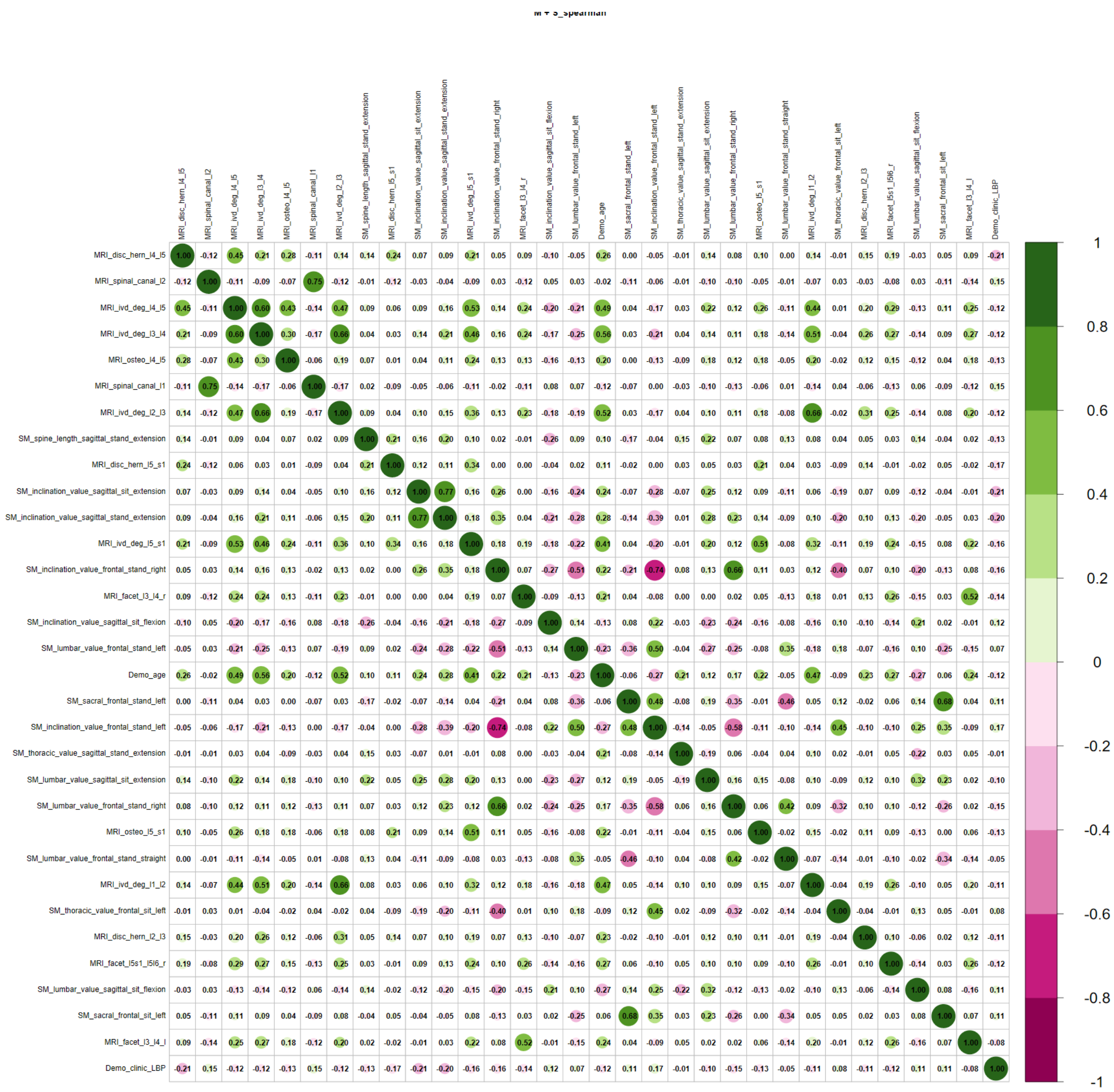

Supplementary Figure S15. Spearman correlation matrix of Boruta selected important features for back shape and function + MRI dataset

Supplementary Table S33. Boruta selected important features for questionnaire and superficial spine morphology dataset

Modality	Variable	selected (%)	Importance (mean)
Questionnaire	SF36 social function	100	19.85
Questionnaire	SF36 psychological well being	100	19.21
Questionnaire	hip pain	100	10.33
Questionnaire	smoking pack years	100	8.62
Back shape & function	inclination value frontal stand left	100	6.52

Demographic	age	80	5.34
Back shape & function	inclination value frontal sit left	90	5.2
Questionnaire	family back pain	100	5.17
Back shape & function	inclination value sagittal stand flexion	10	5.09
Questionnaire	SF36 emotional role function	20	4.97
Questionnaire	physical activity 150min/week	90	4.95
Back shape & function	thoracic value frontal stand left	50	4.93
Back shape & function	inclination value frontal sit right	10	4.83
Back shape & function	sacral frontal stand left	60	4.63
Back shape & function	thoracic value frontal sit left	80	4.61
Back shape & function	lumbar value sagittal sit flexion	60	4.58
Back shape & function	inclination value frontal stand right	20	4.45
Back shape & function	inclination value sagittal stand extension	60	4.24
Questionnaire	family conflicts	20	4.24
Back shape & function	lumbar value sagittal stand extension	10	4.01
Back shape & function	lumbar value frontal stand right	30	3.94
Back shape & function	spine length sagittal stand extension	30	3.91
Back shape & function	inclination value frontal sit straight	20	3.88
Back shape & function	lumbar value frontal sit right	30	3.75
Back shape & function	sacral sagittal stand extension	10	3.75
Back shape & function	sacral frontal sit straight	40	3.73
Back shape & function	inclination value sagittal sit extension	40	3.63
Back shape & function	sacral frontal sit left	10	3.5

SF36 - Short-form 36 Health Status Questionnaire

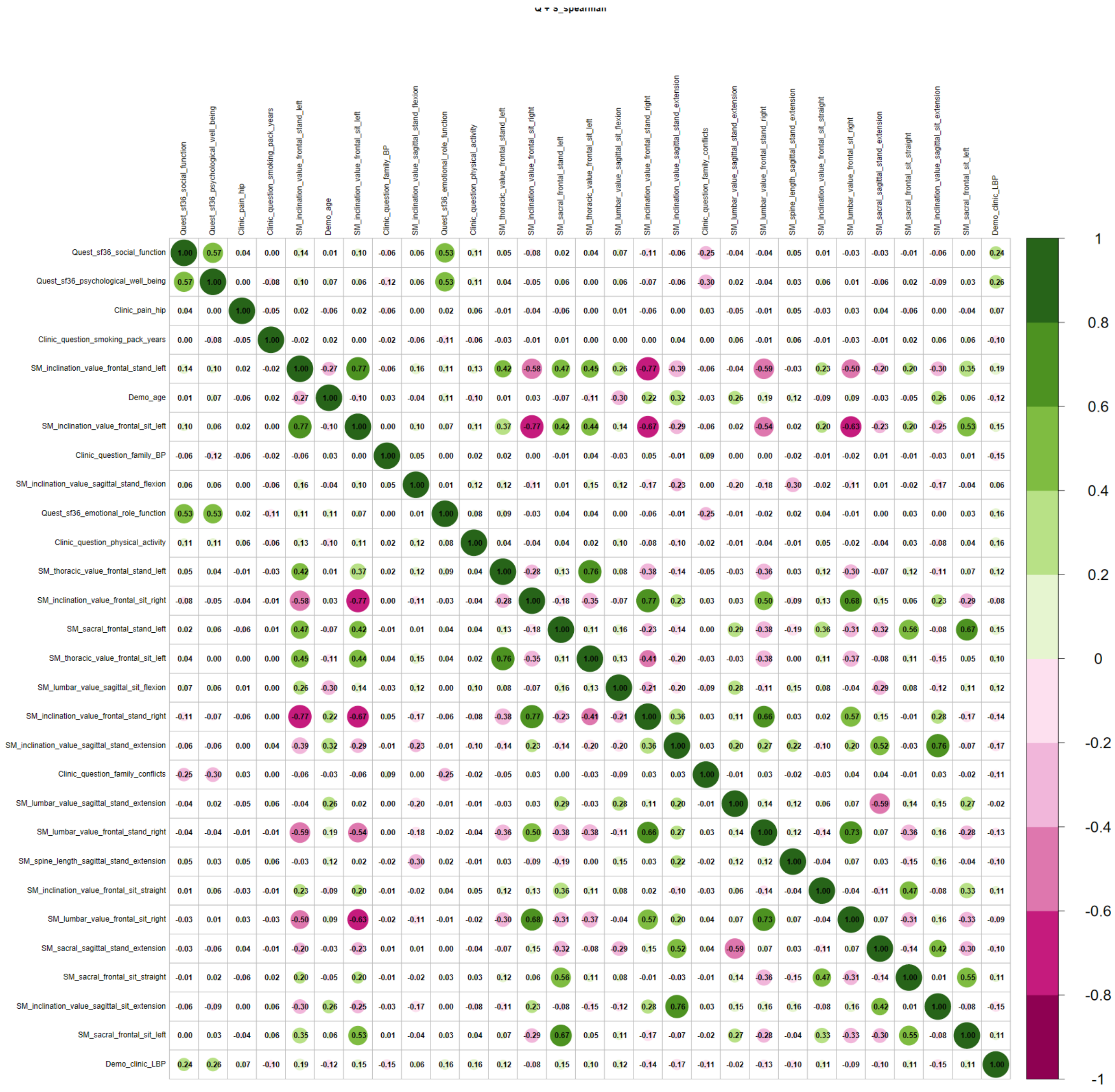

Supplementary Figure S16. Spearman correlation matrix of Boruta selected important features for questionnaire + back shape and function dataset

Supplementary Table S34. Boruta selected important features for questionnaire clinical assessment, and MRI dataset

Modality	Assessment	Selected (%)	Importance (mean)
questionnaire	SF-36 psychological well being	100	13.36
questionnaire	SF-36 social function	100	13.07
questionnaire	hip pain	100	9.71
clinical examination	cervical axial rotate left	100	9.47
MRI	IVD herniation L4 - L5	100	9.12

clinical examination	general mobility	100	7.96
MRI	IVD degeneration L2 - L3	100	7.6
clinical examination	hip flexion left	100	7.19
MRI	spinal canal width L2	100	6.72
clinical examination	sit to stand 30sec	100	6.4
MRI	IVD degeneration L4 - L5	100	6.32
clinical examination	hip internal rotate right	100	6.22
clinical examination	hip flexion right	100	6.09
MRI	IVD degeneration L5 - S1	100	6.04
clinical examination	hip abduction left	100	5.59
MRI	osteochondrosis L5 - S1	90	5.29
clinical examination	hip abduction right	50	5.05
MRI	IVD herniation L5 - S1	90	5
clinical examination	cervical axial rotate right	100	4.83
MRI	IVD degeneration L3 - L4	90	4.75
MRI	facet joint L5/S1 - L5/L6 right	40	4.7
clinical examination	rigid muscle	70	4.63
MRI	osteochondrosis L4 - L5	60	4.47
clinical examination	hip internal rotate left	70	4.18
questionnaire	physical activity 150min/week	20	4.15
MRI	facet joint L3 - L4 right	40	4.07
MRI	spinal canal width L1	40	3.98
questionnaire	living situation	10	3.94
Demographic	age	40	3.9
questionnaire	SF-36 emotional role function	10	3.83
clinical examination	square shoulders	20	3.75
MRI	IVD degeneration L1 - L2	20	3.65
questionnaire	family back pain	10	3.41
clinical examination	thoracic lumbar inclination	10	3.35

IVD – intervertebral disc, SF36 - Short-form 36 Health Status Questionnaire

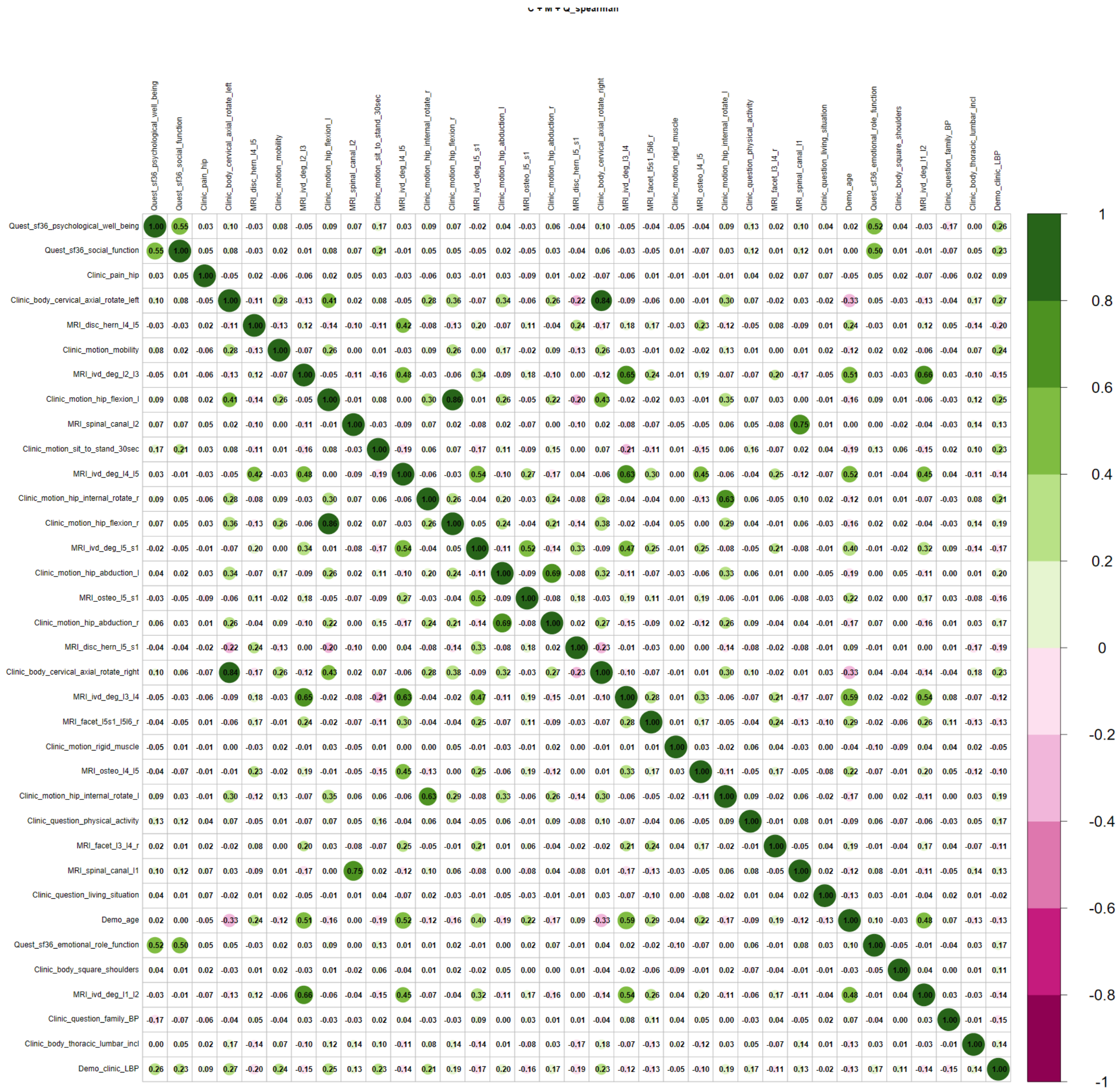

Supplementary Figure S17. Spearman correlation matrix of Boruta selected important features for questionnaire + clinical assessment + MRI dataset

Supplementary Table S35. Boruta selected important features for clinical assessment, MRI, and superficial spine morphology dataset

Modality	Variable	selected (%)	Importance (mean)
Clinical	cervical axial rotate left	100	13.13
MRI	intervertebral disc herniation L4 - L5	100	12.85
Clinical	hip flexion left	100	10.11
Clinical	sit to stand 30sec	100	8.61
Clinical	general mobility	100	8.54

Clinical	hip flexion right	100	8.24
MRI	spinal canal stenosis L2	100	7.24
MRI	intervertebral disc degeneration L2 - L3	100	6.58
MRI	intervertebral disc degeneration L4 - L5	100	6.39
MRI	osteochondrosis intervertebralis L4 - L5	100	6.38
MRI	intervertebral disc degeneration L3 - L4	100	6.07
Clinical	cervical axial rotate right	100	6.06
Clinical	rigid muscle	100	5.6
MRI	intervertebral disc degeneration L5 - S1	100	5.46
Clinical	thoracic lumbar inclination	10	5.4
MRI	osteochondrosis intervertebralis L5 - S1	90	5.3
MRI	facet joint L3 - L4 right	20	5.19
Clinical	hip internal rotate right	70	4.88
MRI	spinal canal stenosis L1	70	4.82
MRI	intervertebral disc herniation L5 - S1	90	4.66
Clinical	hip internal rotate left	40	4.52
Clinical	chin sternum distance	70	4.41
MRI	intervertebral disc degeneration L1 - L2	40	4.41
Back shape & function	inclination value sagittal sit flexion	20	4.34
Back shape & function	inclination value sagittal stand extension	40	4.27
Back shape & function	lumbar value sagittal sit extension	40	4.18
Back shape & function	lumbar value frontal stand right	20	4.18
Back shape & function	inclination value frontal stand right	20	4.05
	inclination value sagittal sit extension	60	4
MRI	facet joint L5/S1 - L5/L6 right	30	3.96
MRI	intervertebral disc herniation L3 - L4	30	3.95
Clinical	hip abduction left	30	3.91
Demographic	age	50	3.84
Clinical	asymmetrical waist triangle	10	3.78
Back shape & function	thoracic value frontal stand right	10	3.67
Back shape & function	inclination value frontal stand left	10	3.64
Clinical	hip abduction right	40	3.6
Back shape & function	spine length sagittal stand extension	20	3.51
Back shape & function	thoracic value sagittal stand extension	10	3.51
MRI	facet joint L5/S1 - L5/L6 left	40	3.5

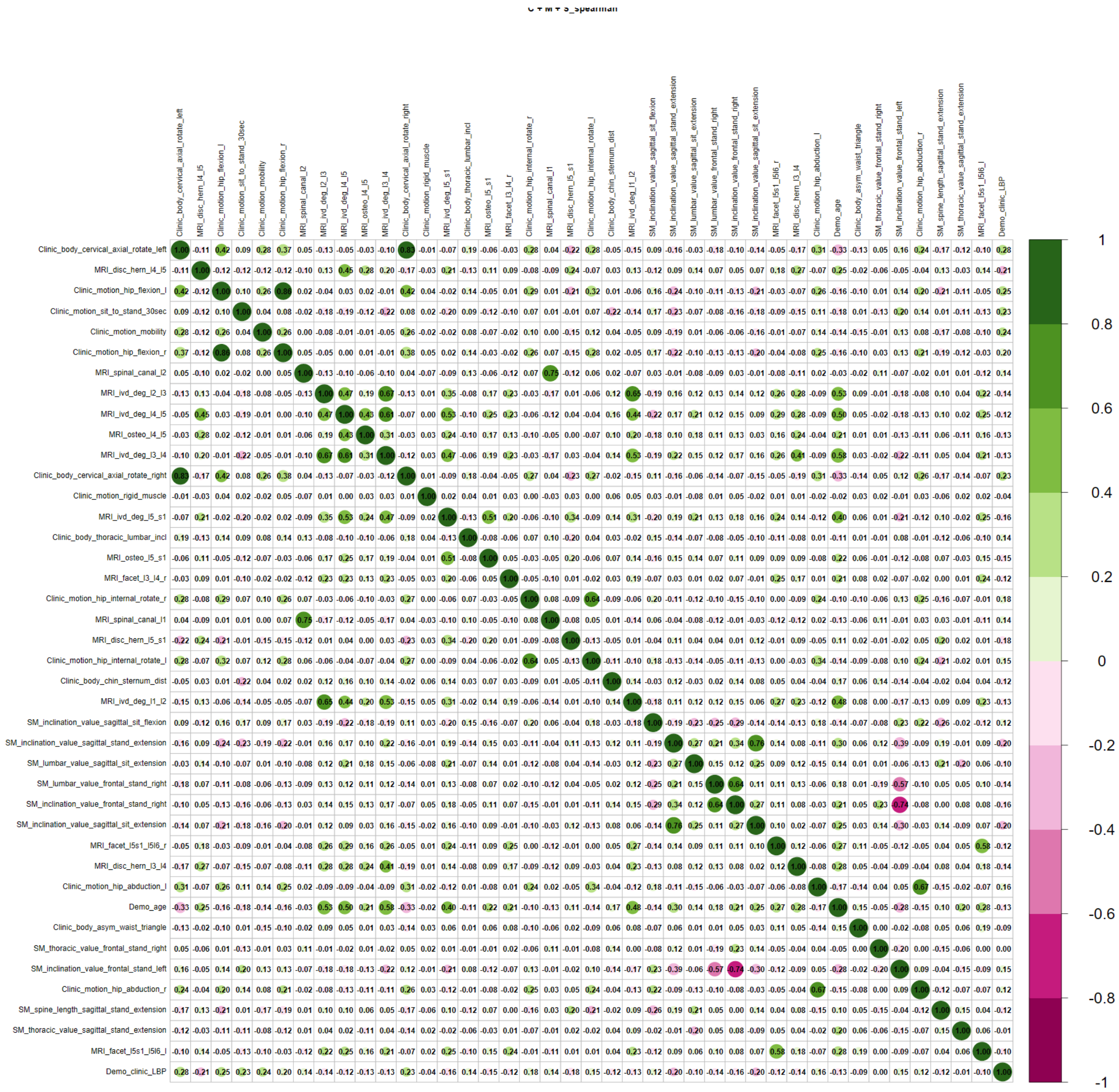

Supplementary Figure S18. Spearman correlation matrix of Boruta selected important features for MRI + clinical assessment + back shape and function dataset

Supplementary Table S36. Boruta selected important features for questionnaire clinical assessment, and superficial spine morphology dataset

Modality	Assessment	Selected (%)	Importance (mean)
Questionnaire	SF36 psychological well being	100	16.99
Questionnaire	SF36 social function	100	16.03
Questionnaire	hip pain	100	11.21
Clinical	mobility	100	10.6

Clinical	cervical axial rotate left	100	10.18
Clinical	rigid muscle	100	8.56
Clinical	sit to stand 30sec	100	8.11
Clinical	smoking pack years	100	8.06
Clinical	physical activity 150min/week	90	6.14
Back shape & function	inclination value frontal sit left	20	5.39
Clinical	hip abduction left	100	5.27
Clinical	family back pain	100	5.25
Demographic	age	100	5.09
Clinical	cervical axial rotate right	100	4.95
Back shape & function	inclination value frontal stand left	100	4.88
Back shape & function	sacral frontal sit straight	50	4.72
Back shape & function	sacral frontal stand left	80	4.57
Questionnaire	SF36 emotional role function	40	4.31
Back shape & function	inclination value frontal sit straight	10	4.31
Clinical	hip flexion right	90	4.25
Clinical	back form	80	4.24
Back shape & function	lumbar value frontal sit right	10	4.11
Clinical	hip abduction right	70	4.08
Back shape & function	lumbar value frontal stand right	10	4.08
Back shape & function	thoracic value frontal stand left	40	4.06
Clinical	hip flexion left	80	3.94
Clinical	family conflicts	30	3.89
Back shape & function	sacral frontal sit left	10	3.87
Back shape & function	thoracic value frontal sit left	30	3.84
Clinical	hip internal rotate right	20	3.83
Clinical	thoracic lumbar lat bend right	80	3.81
Clinical	shober	10	3.73
Back shape & function	inclination value sagittal stand extension	50	3.65
Back shape & function	spine length sagittal stand extension	10	3.62
Back shape & function	lumbar value sagittal stand extension	20	3.59
Back shape & function	lumbar value sagittal sit flexion	10	3.48
Back shape & function	thoracic value sagittal stand extension	10	3.37
Back shape & function	sacral sagittal stand extension	10	3.36
Questionnaire	job load 1	10	3.32
Back shape & function	inclination value sagittal sit extension	10	3.31

SF36 - Short-form 36 Health Status Questionnaire

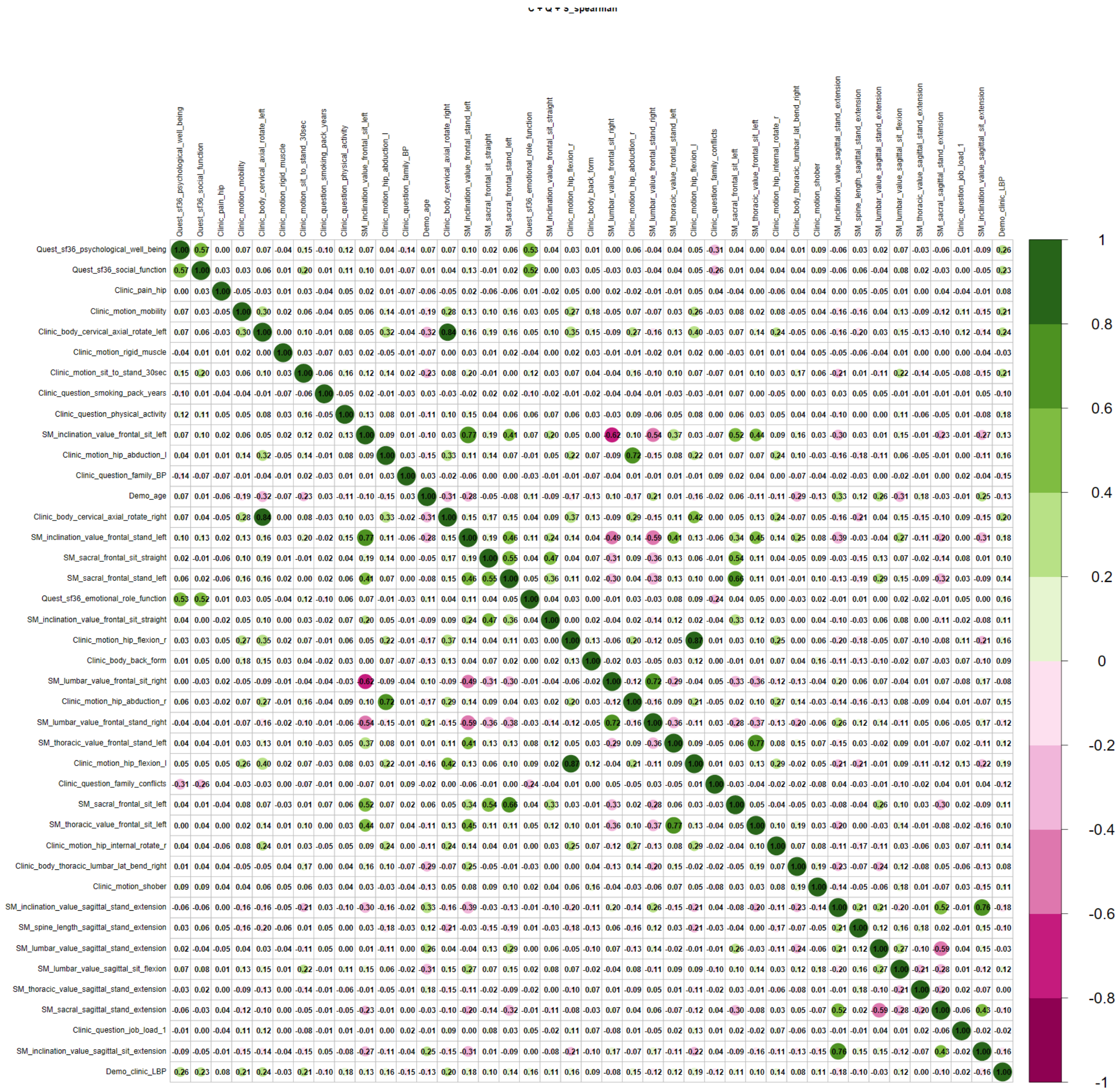

Supplementary Figure S19. Spearman correlation matrix of Boruta selected important features for questionnaire + clinical assessment + back shape and function dataset

Supplementary Table S37. Boruta selected important features for questionnaire, superficial spine morphology, and MRI dataset

Modality	Variable	Selected (%)	Importance (mean)
Questionnaire	SF36 social function	100	16.92
Questionnaire	SF36 psychological well being	100	15.13
MRI	intervertebral disc herniation L4 - L5	100	12.69
MRI	spinal canal stenosis L2	100	9.91

Questionnaire	hip pain	100	8
MRI	intervertebral disc degeneration L4 - L5	100	7.79
MRI	intervertebral disc degeneration L2 - L3	100	7.64
Back shape & function	spine length sagittal stand extension	100	6.97
MRI	intervertebral disc degeneration L5 - S1	100	6.46
MRI	spinal canal stenosis L1	100	5.92
Back shape & function	inclination value sagittal stand extension	90	5.37
MRI	facet joint L3 - L4 right	50	5.27
MRI	osteochondrosis intervertebralis L5 - S1	20	5.15
Back shape & function	lumbar value sagittal sit extension	30	5.13
MRI	intervertebral disc degeneration L3 - L4	70	5.03
Back shape & function	inclination value frontal stand left	20	4.97
Back shape & function	inclination value sagittal sit extension	50	4.95
MRI	intervertebral disc herniation L5 - S1	70	4.79
Questionnaire	physical activity 150min/week	20	4.61
Back shape & function	sacral sagittal stand extension	60	4.59
MRI	osteochondrosis intervertebralis L4 - L5	70	4.55
Back shape & function	lumbar value frontal stand right	10	4.45
MRI	spinal canal stenosis L3	10	4.44
Questionnaire	IPAQ met sum	30	4.41
Back shape & function	sacral frontal sit straight	10	4.35
Demographic	age	10	4.19
Back shape & function	sacral frontal sit left	10	4.13
Back shape & function	inclination value sagittal sit flexion	20	4.01
Back shape & function	thoracic value frontal sit left	10	3.97
Back shape & function	lumbar value frontal stand left	10	3.92
MRI	facet joint L5/S1 - L5/L6 right	10	3.66
MRI	facet joint L4 - L5 right	10	3.65
MRI	intervertebral disc degeneration L1 - L2	10	3.62
Questionnaire	SF36 emotional role function	10	3.29

SF36 - Short-form 36 Health Status Questionnaire

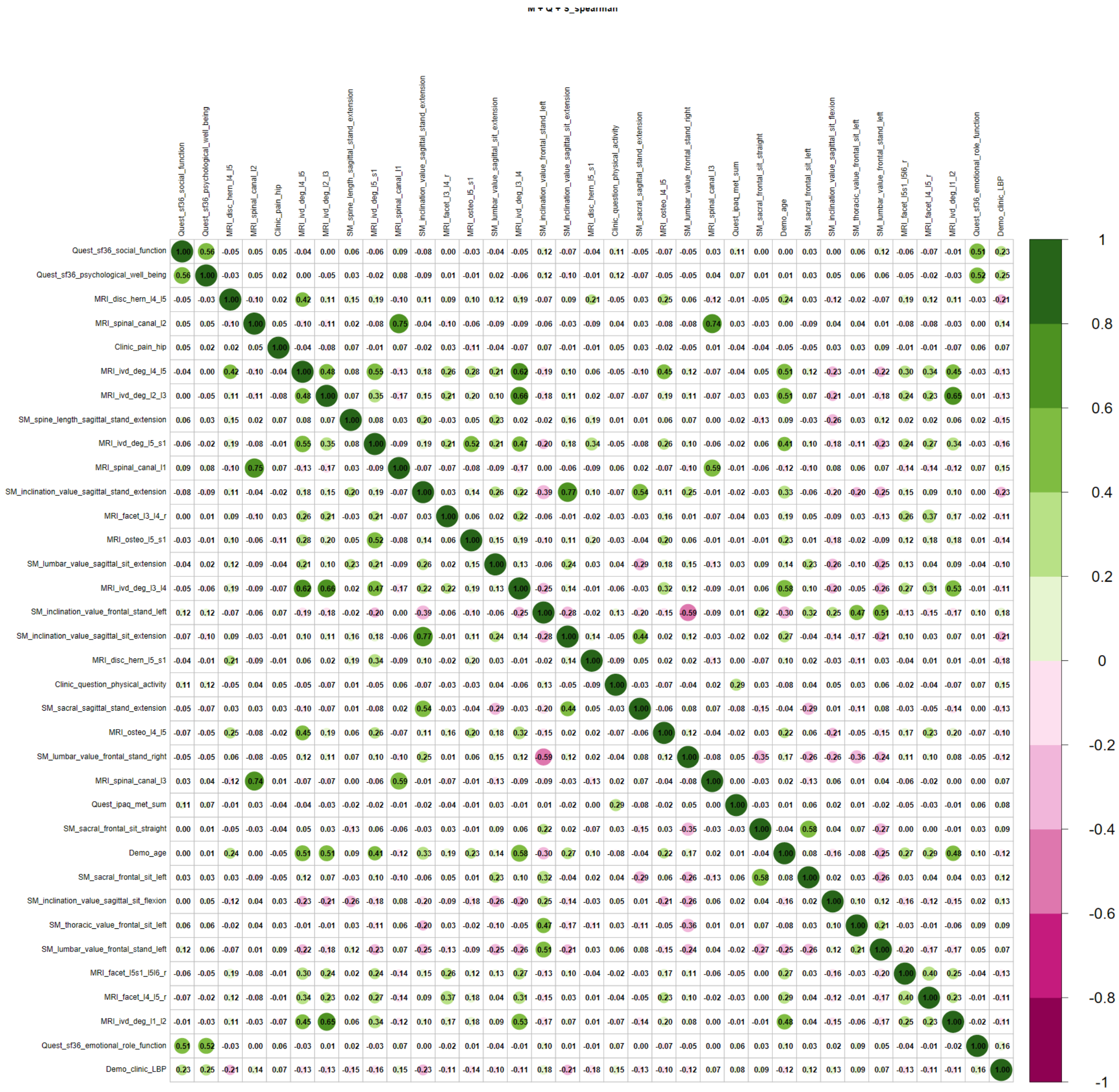

Supplementary Figure S20. Spearman correlation matrix of Boruta selected important features for MRI + questionnaire + back shape and function dataset

Supplementary Table S38. Boruta selected important features for clinical assessment, MRI, questionnaire, and superficial spine morphology dataset

Modality	Assessment	Selected (%)	Importance (mean)
Questionnaire	SF36 social function	100	13.04
Questionnaire	SF36 psychological well being	100	11.58
Questionnaire	Clinic pain hip	100	9.43
MRI	intervertebral disc herniation L4 - L5	100	8.61
Clinical	cervical axial rotate left	100	8.6

MRI	intervertebral disc degeneration L2 - L3	100	6.8
Clinical	hip flexion left	100	6.79
Clinical	general mobility	100	6.62
MRI	intervertebral disc degeneration L4 - L5	100	6.45
MRI	spinal canal stenosis L2	90	6.28
MRI	intervertebral disc degeneration L5 - S1	100	5.81
Clinical	hip flexion right	100	5.68
Clinical	sit to stand 30sec	100	5.65
Clinical	hip internal rotate right	90	5.62
MRI	osteochondrosis intervertebralis L5 - S1	90	5.09
Back shape & function	lumbar value sagittal sit extension	60	5.01
MRI	spinal canal stenosis L1	10	4.96
Clinical	hip abduction left	80	4.8
Back shape & function	spine length sagittal stand extension	70	4.75
Clinical	rigid muscle	60	4.75
Back shape & function	inclination value sagittal stand extension	100	4.73
Questionnaire	SF36 emotional role function	20	4.65
MRI	osteochondrosis intervertebralis L4 - L5	80	4.63
Clinical	cervical axial rotate right	90	4.62
Back shape & function	sacral sagittal stand extension	10	4.5
Clinical	Clinic question physical activity	60	4.47
MRI	intervertebral disc degeneration L3 - L4	80	4.43
MRI	facet joint L3 - L4 right	10	4.41
Back shape & function	inclination value sagittal sit extension	60	4.34
Clinical	hip abduction right	30	4.27
Clinical	hip internal rotate left	40	4.02
MRI	intervertebral disc herniation L5 - S1	70	3.97
Demographic	age	10	3.77
Back shape & function	inclination value sagittal sit flexion	10	3.68
Clinical	thomas handle right	10	3.41

SF36 - Short-form 36 Health Status Questionnaire, IPAQ – International Physical Activity Questionnaire

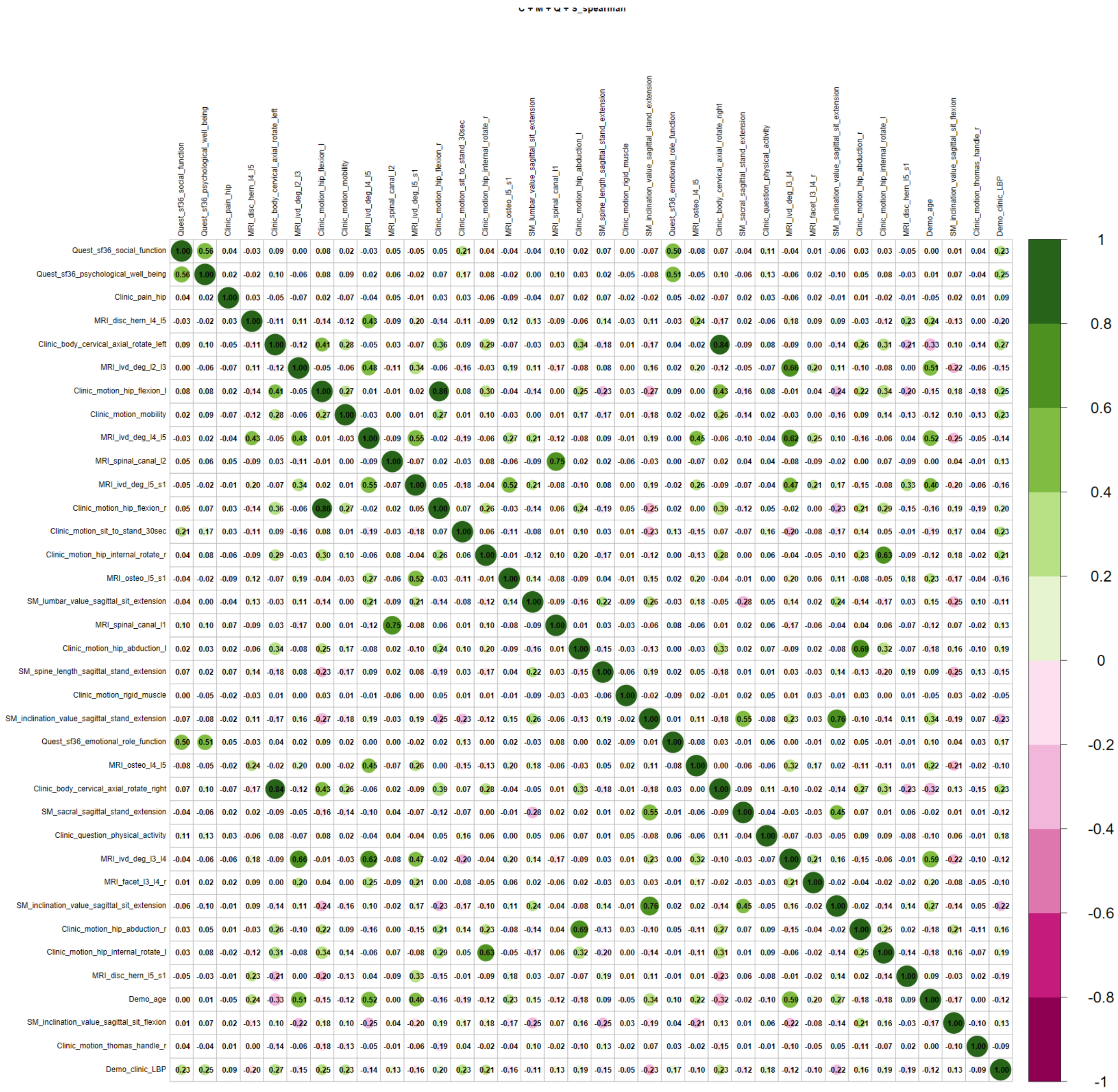

Supplementary Figure S21. Spearman correlation matrix of Boruta selected important features for questionnaire + clinical assessment + back shape and function + MRI dataset

Supplementary Table S39. Overall feature importance and robustness across all modality datasets.

Modality	Variable	selected (%)	Importance (mean)
Questionnaire	SF36 social function	100	16.54
Questionnaire	SF36 psychological well being	100	16.44
MRI	intervertebral disc herniation L4 - L5	100	13.33
Clinic	cervical axial rotate left	100	11.76
Questionnaire	Clinic pain hip	100	10.57
Clinic	mobility	100	10.03

Clinic	sit to stand 30sec	100	9.17
MRI	spinal canal stenosis L2	98.75	8.63
Clinic	rigid muscle	91.25	7.36
MRI	intervertebral disc degeneration L4 - L5	100	7.28
MRI	intervertebral disc degeneration L2 - L3	100	7.21
Clinic	hip flexion left	93.75	6.88
MRI	intervertebral disc degeneration L3 - L4	91.25	6.1
Clinic	hip flexion right	97.5	5.97
MRI	osteochondrosis intervertebralis L4 - L5	78.75	5.92
Clinic	cervical axial rotate right	98.75	5.89
MRI	spinal canal stenosis L1	77.5	5.88
MRI	intervertebral disc degeneration L5 - S1	95	5.7
MRI	intervertebral disc herniation L5 - S1	90	5.32
Questionnaire	physical activity 150min/week	58.75	5.04
Clinic	hip abduction left	77.5	5.01
MRI	facet joint L3 - L4 right	57.5	4.97
MRI	osteochondrosis intervertebralis L5 - S1	67.5	4.88
Questionnaire	smoking pack years	50	4.76
Back shape & function	inclination value frontal stand left	58.75	4.49
Clinic	hip internal rotate right	65	4.46
Back shape & function	inclination value sagittal stand extension	57.5	4.46
Clinic	hip abduction right	50	4.3
Back shape & function	inclination value sagittal sit extension	52.5	4.28
MRI	facet joint L5/S1 - L5/L6 right	48.75	4.07
Back shape & function	spine length sagittal stand extension	50	4.04
Back shape & function	lumbar value frontal stand right	37.5	3.69
MRI	intervertebral disc degeneration L1 - L2	38.75	3.64
Clinic	family back pain	51.25	3.45
Back shape & function	sacral frontal stand left	46.25	3.15
Questionnaire	SF36 emotional role function	13.75	3.09
Back shape & function	sacral frontal sit left	20	2.92
Back shape & function	lumbar value sagittal sit extension	28.75	2.76
Clinic	hip internal rotate left	35	2.71
Back shape & function	thoracic value sagittal stand extension	30	2.65
Back shape & function	inclination value frontal stand right	32.5	2.63
Clinic	back form	43.75	2.59
Clinic	thoracic lumbar inclination	15	2.59
Back shape & function	thoracic value frontal sit left	28.75	2.54
Back shape & function	inclination value frontal sit left	26.25	2.3
Clinic	chin sternum distance	31.25	2.16
Back shape & function	thoracic value frontal stand left	25	2.16
Clinic	thoracic lumbar lat bend right	33.75	2.08
Back shape & function	inclination value sagittal sit flexion	7.5	2.06

MRI	facet joint L3 - L4 left	21.25	2.05
Back shape & function	sacral frontal sit straight	18.75	2.05
Clinic	shober	11.25	2.05
Back shape & function	sacral sagittal stand extension	11.25	2.02
Back shape & function	lumbar value sagittal sit flexion	22.5	1.98
Back shape & function	lumbar value sagittal stand extension	6.25	1.88
Clinic	cervical lateral bend left	13.75	1.62
Questionnaire	living situation	7.5	1.56
MRI	facet joint L5/S1 - L5/L6 left	23.75	1.54
Questionnaire	job load 1	11.25	1.51
Back shape & function	lumbar value frontal sit right	7.5	1.49
MRI	intervertebral disc herniation L3 - L4	8.75	1.44
Clinic	asymmetrical waist triangle	7.5	1.36
MRI	spinal canal stenosis L3	3.75	1.05
Back shape & function	lumbar value frontal stand left	7.5	1.04
Questionnaire	BRSQ intrinsic motivation	6.25	1.02
Questionnaire	family conflicts	6.25	1.02
Back shape & function	inclination value frontal sit straight	3.75	1.02
MRI	spinal canal stenosis L4	8.75	1.01
MRI	intervertebral disc herniation L2 - L3	15	1
Back shape & function	inclination value frontal sit right	2.5	1
Back shape & function	lumbar value frontal stand straight	8.75	0.99
MRI	facet joint L4 - L5 right	3.75	0.98
Clinic	square shoulders	3.75	0.98
Clinic	thomas handle right	2.5	0.93
Back shape & function	thoracic value frontal stand right	2.5	0.89
MRI	facet joint L1 - L2 right	3.75	0.64
Back shape & function	inclination value sagittal stand flexion	1.25	0.64
MRI	facet joint L1 - L2 left	2.5	0.57
Questionnaire	IPAQ met sum	3.75	0.55
Clinic	cervical inclination	1.25	0.54
Back shape & function	lumbar value frontal sit straight	1.25	0.53
Clinic	hip extension right	1.25	0.52
Back shape & function	sacral sagittal sit straight	1.25	0.51
Questionnaire	BRSQ integrated regulation	2.5	0.48
Clinic	lumbar bulge	1.25	0.48
Clinic	thoracic lumbar axial rotation left	1.25	0.48
Clinic	roussoly type	1.25	0.46
Back shape & function	sacral frontal stand straight	1.25	0.46
Clinic	cervical reclination	5	0.45
Back shape & function	thoracic value sagittal sit extension	2.5	0.45
Clinic	finger floor distance	1.25	0.45
Clinic	hip adduction right	1.25	0.42
Back shape & function	sacral sagittal stand straight	2.5	0.41

Supplementary Data 4: Univariate statistics

Supplementary Table S40: Demographic Wilcoxon-Mann-Whitney test (continuous and ordinal)

Median values and corresponding IQR for patients and controls with statistical u-values, z-values, r-values and p-values of the Wilcoxon-Mann-Whitney test after FWE correction.

Feature	Patients		Controls		Statistic			
	Median	IQR	Median	IQR	u-value	z-value	r-value	p-value
Age	44.0	20.25	39.0	22.00	320583	4.08	0.120	< .001
BMI	23.3	4.03	23.5	3.86	295745	-0.30	-0.009	1

BMI – Body mass index

Supplementary Table S41: Demographic Chi2 Test (nominal)

Statistical Chi^2-values, ω-values and p-values of the Chi-Square test after FWE correction.

Feature	Statistic		
	x2-value	Cohen's ω	p-value
Sex	3.37	0.05	.205

Supplementary Table S42: Questionnaire Wilcoxon-Mann-Whitney test (continuous and ordinal)

Median values and corresponding IQR for patients and controls with statistical u-values, z-values, r-values and p-values of the Wilcoxon-Mann-Whitney test after FWE correction. As some ordinal variables (e.g. “Alcohol frequency”) were expressed as text, only statistical results are shown in the table.

Variable	Patients		Controls		Statistic			
	Median	IQR	Median	IQR	u-value	z-value	r-value	p-value
Alcohol frequency					216527	0.91	0.029	1
Alcohol glasses					211454	-0.33	-0.011	1
BRSQ external regulation	3	3	3	2	215646	0.69	0.022	1
BRSQ integrated regulation	9	4	9	4	205967	-1.53	-0.049	1
BRSQ intrinsic motivation	10	4	10	3	198539	-3.27	-0.104	.019
COVID19 activity					217410	1.44	0.046	1
IPAQ met sum	834	868	910	772	206393	-1.42	-0.045	1
Job load 1					225160	3.67	0.117	.005
Job load 1 duration	8	3	7	3	214772	0.48	0.015	1
Physical activity					194505	-4.77	-0.152	< .001
SF36 emotional role function	100	33	100	0	196952	-4.39	-0.140	< .001
SF36 psychological well-being	72	20	80	16	178507	-7.74	-0.247	< .001
SF36 social function	88	38	100	13	183751	-7.08	-0.225	< .001
Smoking pack years	0	0	0	0	221047	3.33	0.106	.016

SRBAI sum	16	8	17	7	201693	-2.49	-0.079	.206

BRSQ - Behavioural Regulation in Sport Questionnaire, IPAQ – International Physical Activity Questionnaire, SF36 - Short-form 36 Health Status Questionnaire, SRBAI - Self-Report Behavioural Automaticity Index

Supplementary Table S43: Questionnaire Chi2 Test (nominal)

Statistical Chi^2-values, ω-values and p-values of the Chi-Square test after FWE correction.

Variable	Statistic		
	x2-value	Cohen's ω	p-value
Family back pain	20.88	0.15	.004
Family conflicts	9.95	0.10	.007
Hip pain	40.17	0.20	.004
Job field	28.63	0.17	.112
Job posture	5.37	0.07	.303
Living situation	13.86	0.12	.006
Prior surgeries	2.25	0.05	.303
Psychological stress	13.37	0.12	.007

Supplementary Table S44: Clinical Physcial Assessment Wilcoxon-Mann-Whitney test (continuous and ordinal)

Median values and corresponding IQR for patients and controls with statistical u-values, z-values, r-values and p-values of the Wilcoxon-Mann-Whitney test after FWE correction.

Variable	Patients		Controls		Statistic			
	Median	IQR	Median	IQR	u-value	z-value	r-value	p-value
Cervical axial rotate left	70	15	80	20	221595	-7.98	-0.243	< .001
Cervical axial rotate right	75	20	80	20	227209	-6.86	-0.209	< .001
Cervical inclination	45	10	45	10	256777	-0.97	-0.029	1
Cervical lateral bend left	35	10	35	15	248306	-2.65	-0.081	.225
Cervical lateral bend right	35	10	40	15	245196	-3.27	-0.099	.033
Cervical reclination	45	25	50	20	253672	-1.57	-0.048	1
Chin sternum distance	2	2	1	2	280047	3.92	0.119	.003
Finger floor distance	0	16	0	18	279513	3.55	0.108	.013
Hip abduction left	50	15	50	15	237322	-4.83	-0.147	< .001
Hip abduction right	50	15	50	15	240564	-4.19	-0.128	< .001
Hip adduction left	25	10	25	10	260121	-0.30	-0.009	1
Hip adduction right	25	10	30	10	260129	-0.30	-0.009	1
Hip extension left	10	0	10	0	252058	-2.32	-0.071	.529
Hip extension right	10	0	10	0	253208	-2.02	-0.062	1
Hip external rotate left	40	11	40	15	268678	1.42	0.043	1
Hip external rotate right	40	15	40	15	262804	0.24	0.007	1
Hip flexion left	120	15	125	20	231410	-5.99	-0.182	< .001

Hip flexion right	120	10	130	20	236225	-5.03	-0.153	< .001
Hip internal rotate left	30	15	35	10	244180	-3.49	-0.106	.016
Hip internal rotate right	30	15	35	10	240555	-4.21	-0.128	< .001
OTT	3	1	3	1	253072	-1.69	-0.052	1
Roussoly type	2	0	2	1	248275	-2.99	-0.091	.080
Shober	5	1	5	2	243079	-3.68	-0.112	.008
Sit to stand 30sec	21	8	23	7	226819	-6.83	-0.208	< .001
Thoracic lumbar ar left	40	20	40	20	253276	-1.65	-0.050	1
Thoracic lumbar ar right	40	20	40	20	253955	-1.51	-0.046	1
Thoracic lumbar inclination	70	21	70	30	244361	-3.40	-0.104	.021
Thoracic lumbar lateral bend left	30	15	30	10	250461	-2.22	-0.068	.655
Thoracic lumbar lateral bend right	30	15	35	10	249415	-2.43	-0.074	.403
Thoracic lumbar reclination	30	15	30	15	258021	-0.71	-0.022	1

Supplementary Table S45: Clinical Physcial Assessment Chi2 Test (nominal)

Statistical Chi^2-values, ω-values and p-values of the Chi-Square test after FWE correction.

Variable	Statistic		
	x2-value	Cohen's ω	p-value
Asymmetrical waist triangle	7.82	0.09	.045
Back form	24.70	0.15	.006
Lumbar bulge	13.51	0.11	.018
Mobility	46.81	0.21	.006
Pelvic tilt frontal	0.52	0.02	.790
Pelvic tilt sagital	7.11	0.08	.130
Plump line	4.73	0.07	.130
Rib hump	12.17	0.11	.024
Rigid muscle	50.32	0.22	.006
Sagittal balance	2.15	0.04	.317
Square shoulders	15.64	0.12	.010
Thomas handle left	6.61	0.08	.090
Thomas handle right	5.23	0.07	.130

Supplementary Table S46: Superficial spine morphology Wilcoxon-Mann-Whitney test (continuous)

Variable	Patients		Controls		Statistic			
	Median	IQR	Median	IQR	u-value	z-value	r-value	p-value
Inclination value frontal sit left	18	11	20	11	248689	-4.45	-0.133	< .001
Inclination value frontal sit right	-24	10	-26	11	287297	2.81	0.084	0.165
Inclination value frontal sit straight	-4	3	-3	2	255324	-3.24	-0.097	0.046

Inclination value frontal stand left	20	11	24	11	240941	-5.91	-0.177	< .001
Inclination value frontal stand right	-27	10	-29	10	298681	4.95	0.148	< .001
Inclination value frontal stand straight	-3	3	-3	3	272113	-0.05	-0.001	1
Inclination value sagittal sit extension	-14	11	-17	12	299339	5.07	0.152	< .001
Inclination value sagittal sit flexion	84	14	85	14	260158	-2.3	-0.069	0.628
Inclination value sagittal sit straight	5	5	5	4	279373	1.32	0.04	1
Inclination value sagittal stand extension	-19	10	-21	11	298587	4.93	0.148	< .001
Inclination value sagittal stand flexion	116	21	118	21	266091	-1.18	-0.035	1
Inclination value sagittal stand straight	1	3	1	3	276904	0.86	0.026	1
Lumbar value frontal sit left	16	9	17	9	259322	-2.45	-0.074	0.423
Lumbar value frontal sit right	-16	11	-17	12	287942	2.93	0.088	0.12
Lumbar value frontal sit straight	1	6	1	6	282311	1.87	0.056	1
Lumbar value frontal stand left	20	11	21	10	263580	-1.65	-0.05	1
Lumbar value frontal stand right	-17	12	-20	12	296706	4.57	0.137	< .001
Lumbar value frontal stand straight	3	8	2	9	284300	2.24	0.067	0.671
Lumbar value sagittal sit extension	-33	14	-34	14	283974	2.18	0.065	0.759
Lumbar value sagittal sit flexion	23	13	25	11	253884	-3.48	-0.104	0.02
Lumbar value sagittal sit straight	-14	15	-13	14	263916	-1.59	-0.048	1
Lumbar value sagittal stand extension	-36	12	-37	14	278643	1.18	0.035	1
Lumbar value sagittal stand straight	-28	12	-27	11	264400	-1.5	-0.045	1
Sacral frontal sit left	-1	6	0	5	252795	-3.69	-0.111	0.009
Sacral frontal sit right	-4	5	-4	6	265294	-1.33	-0.04	1
Sacral frontal sit straight	-4	5	-3	4	256209	-3.05	-0.091	0.082
Sacral frontal stand left	-3	8	-1	7	247591	-4.66	-0.14	< .001
Sacral frontal stand right	-6	5	-6	7	278563	1.17	0.035	1
Sacral frontal stand straight	-5	5	-4	5	259268	-2.47	-0.074	0.418
Sacral sagittal sit extension	8	13	6	14	282534	1.91	0.057	1
Sacral sagittal sit flexion	47	15	46	17	274473	0.39	0.012	1
Sacral sagittal sit straight	8	10	7	10	281418	1.7	0.051	1
Sacral sagittal stand extension	5	17	2	15	287444	2.83	0.085	0.157
Sacral sagittal stand flexion	78	22	79	23	273788	0.27	0.008	1

Sacral sagittal stand straight	14	10	13	10	284501	2.28	0.068	0.632
Spine length sagittal stand extension	495	63	486	55	288916	3.11	0.093	0.07
Thoracic value frontal sit left	33	16	34	14	258113	-2.68	-0.08	0.235
Thoracic value frontal sit right	-37	12	-37	13	272398	0	0	1
Thoracic value frontal sit straight	-3	10	-2	9	262542	-1.85	-0.055	1
Thoracic value frontal stand left	32	16	35	14	252969	-3.65	-0.109	0.011
Thoracic value frontal stand right	-36	14	-36	13	270986	-0.26	-0.008	1
Thoracic value frontal stand straight	-3	9	-2	10	263520	-1.67	-0.05	1
Thoracic value sagittal sit extension	29	21	29	21	270485	-0.35	-0.011	1
Thoracic value sagittal sit flexion	73	12	73	13	275612	0.61	0.018	1
Thoracic value sagittal sit straight	40	14	40	13	277132	0.89	0.027	1
Thoracic value sagittal stand extension	34	18	33	20	268380	-0.75	-0.022	1
Thoracic value sagittal stand flexion	64	16	64	15	274505	0.4	0.012	1
Thoracic value sagittal stand straight	46	14	45	13	275202	0.53	0.016	1

Supplementary Table S47: Spino-pelvic MRI Wilcoxon-Mann-Whitney test (continuous and ordinal)

Median values and corresponding IQR for patients and controls with statistical u-values, z-values, r-values and p-values of the Wilcoxon-Mann-Whitney test after FWE correction.

**Variable**	**Patients**		**Controls**		**Statistic**			
	Median	IQR	Median	IQR	u-value	z-value	r-value	p-value
Intervertebral disc herniation L2-L3	1	0	1	0	142051	3.17	0.112	.018
Intervertebral disc herniation L3-L4	1	0	1	0	144077	3.26	0.115	.016
Intervertebral disc herniation L4-L5	1	1	1	1	153471	6.02	0.213	< .001
Intervertebral disc herniation L5-S1	1	1	1	1	150122	4.75	0.168	< .001
Facet L1-L2 left	1	1	1	1	143273	2.19	0.078	.114
Facet L1-L2 right	1	1	1	1	143551	2.28	0.081	.113
Facet L2-L3 left	1	1	1	1	140482	1.19	0.042	.234
Facet L2-L3 right	1	1	1	1	142246	1.86	0.066	.188
Facet L3-L4 left	1	1	1	1	144439	2.62	0.093	.061
Facet L3-L4 right	2	1	1	1	148587	3.98	0.141	.001
Facet L4-L5 left	2	1	1	1	145545	2.89	0.102	.042
Facet L4-L5 right	2	1	1	1	145101	2.72	0.096	.060
Facet L5-S1 / L5-L6 left	2	1	2	1	145096	2.69	0.095	.060

Facet L5-S1 / L5-L6 right	2	1	2	1	147498	3.49	0.124	.009
Intervertebral disc degeneration L1-L2	2	1	1	1	146699	3.26	0.115	.016
Intervertebral disc degeneration L2-L3	2	1	1	1	147168	3.38	0.120	.011
Intervertebral disc degeneration L3-L4	2	1	1	1	147661	3.51	0.124	.009
Intervertebral disc degeneration L4-L5	2	2	2	2	147691	3.43	0.121	.010
Intervertebral disc degeneration L5-S1	2	1	2	2	150652	4.34	0.154	< .001
Osteochondrosis L4-L5	1	0	1	0	143664	3.50	0.124	.009
Osteochondrosis L5-S1	1	0	1	0	144674	3.60	0.127	.006
Spinal cana stenosis l L1	17.5	1.90	18.0	1.90	123171	-4.35	-0.154	< .001
Spinal canal stenosis L2	16.9	2.25	17.3	1.90	123548	-4.23	-0.150	< .001
Spinal canal stenosis L3	16.5	2.10	16.8	2.20	128327	-2.75	-0.097	.060
Spinal canal stenosis L4	17.0	2.45	17.1	2.33	132006	-1.61	-0.057	.215
Spinal canal stenosis L5	17.4	2.95	17.9	2.90	128906	-2.57	-0.091	.061
